# Genome-Wide Investigation and Expression Analyses of WD40 Protein Family in the Model Plant Foxtail Millet (*Setaria italica* L.)

**DOI:** 10.1371/journal.pone.0086852

**Published:** 2014-01-23

**Authors:** Awdhesh Kumar Mishra, Mehanathan Muthamilarasan, Yusuf Khan, Swarup Kumar Parida, Manoj Prasad

**Affiliations:** National Institute of Plant Genome Research, Aruna Asaf Ali Marg, New Delhi; University of Delhi South Campus, India

## Abstract

WD40 proteins play a crucial role in diverse protein-protein interactions by acting as scaffolding molecules and thus assisting in the proper activity of proteins. Hence, systematic characterization and expression profiling of these *WD40* genes in foxtail millet would enable us to understand the networks of WD40 proteins and their biological processes and gene functions. In the present study, a genome-wide survey was conducted and 225 potential *WD40* genes were identified. Phylogenetic analysis categorized the WD40 proteins into 5 distinct sub-families (I–V). Gene Ontology annotation revealed the biological roles of the WD40 proteins along with its cellular components and molecular functions. *In silico* comparative mapping with sorghum, maize and rice demonstrated the orthologous relationships and chromosomal rearrangements including duplication, inversion and deletion of *WD40* genes. Estimation of synonymous and non-synonymous substitution rates revealed its evolutionary significance in terms of gene-duplication and divergence. Expression profiling against abiotic stresses provided novel insights into specific and/or overlapping expression patterns of *SiWD40* genes. Homology modeling enabled three-dimensional structure prediction was performed to understand the molecular functions of WD40 proteins. Although, recent findings had shown the importance of WD40 domains in acting as hubs for cellular networks during many biological processes, it has invited a lesser research attention unlike other common domains. Being a most promiscuous interactors, WD40 domains are versatile in mediating critical cellular functions and hence this genome-wide study especially in the model crop foxtail millet would serve as a blue-print for functional characterization of WD40s in millets and bioenergy grass species. In addition, the present analyses would also assist the research community in choosing the candidate WD40s for comprehensive studies towards crop improvement of millets and biofuel grasses.

## Introduction

Foxtail millet [*Setaria italica* (L.) P. Beauv.], the second largest cultivated millet species in the world, possesses several salient attributes such as small genome (∼515 Mb; 2n = 2x = 18), relatively lower repetitive DNA, short life-cycle, inbreeding nature and is closely-related to several bioenergy grasses [1], [2]. These features along with its potential abiotic stress tolerance have accentuated this crop as an experimental model system for examining the architectural traits, evolutionary genomics and physiological aspects of C4 panicoid grass crops [2]–[4]. Hence, considering its significance, the US Department of Energy - Joint Genome Institute and the Beijing Genomics Institute, China had sequenced the genome and the draft sequence was released in 2012 [5], [6]. Consequently, the availability of foxtail millet sequence information encouraged the scientific research community to decipher its structural and functional genomics, thus ultimately assisting in crop improvement and ensuring food security [7]. In this regard, we had also reported substantial findings in the aspects of both structural [8]–[13], and functional genomics [14]–[22] in the model crop, foxtail millet.

In our earlier study, we identified and characterized a differentially expressed transcript encoding for WD40 protein from a salinity and dehydration stress-induced subtractive cDNA library [20]. Being the first report, we showed a putative regulation of *SiWD40* expression by dehydration responsive elements (DRE) during abiotic stress [20]. WD40 proteins were identified to play a crucial role in diverse protein-protein interactions by acting as scaffolding molecules and thus assisting the proper activity of the proteins [23]. Structurally, the WD40 domain is characterized by the presence of several copies of WD40 repeats with each repeat containing 44–60 residue units. Each unit includes a glycinehistidine (GH) dipeptide about 11–24 residues from its N terminus and terminates with Trp-Asp (WD) doublet residues at the C-terminus [24], [25]. Each of the repeat folds into four-stranded anti-parallel β-sheet and is proposed to originate from intragenic duplication and recombination events and diversify during evolution [25], [26]. A subset of WD40 proteins have been named as DWD [Damaged DNA binding (DDB) WD40] based on their interaction with DDB1 and CULLIN4 (CUL4) [27]. CUL4– DDB1 ubiquitin E3 ligases use DWD proteins as molecular adaptors for substrate recognition, and modulate multiple biological processes through ubiquitin-dependent proteolysis such as DNA- repair mechanism caused by UV-damage and histone methylation (post-translational modification). These proteins contain 16 conserved amino acids within the WD40 repeats, called “DWD box” [28], [29].

Considering the importance of deciphering the molecular networks, biological processes and gene functions of WD40 proteins, genome-wide investigations have been conducted in *Arabidopsis* [30] and rice [31], but no report was available in foxtail millet till date. Hence, this is the first comprehensive report on genome-wide survey, expression profiling and evolutionary analysis of WD40 proteins in foxtail millet (internally annotated as ‘SiWD40’). We have identified about 225 *SiWD40* genes spanning the nine chromosomes of foxtail millet and classified them into five classes. Sequence comparison of *SiWD40* genes within themselves and with other grasses like sorghum, maize and rice facilitated the study on presence and distribution of paralogous and orthologous *WD40* genes between the grasses. These experimental outcomes have paved a way for further comparative genomic and phylogenetic analyses of WD40 proteins among members of grass family. Subsequently, quantitative real-time PCR (qRT-PCR)-based gene expression profiling showed the temporal and stress-specific expression pattern of candidate *SiWD40* genes. Homology modeling enabled three-dimensional structure prediction was then performed, which would facilitate studies on understanding its molecular function. Positively, this first report will serve as a solid base for functional genomic studies including further molecular characterization of WD40 genes towards various stress responses in foxtail millet.

## Results and Discussion

### Identification of Novel SiWD40 Members in *Setaria italica*

In order to identify the *SiWD40* genes in *Setaria italica*, the characteristic eukaryotes domain sequence of WD40 (GECKXVLXGHTSTVTCVAFSPDGPLLASGSRDGTIKIWD) was generated by hmmemit from HMM profile (PF00400). The BLASTP analysis was performed using this sequence as a query in PHYTOZOME, with a threshold E value of ≤10. This identified a total of 321 sequences and the removal of different transcripts of the same gene identified 225 putative *SiWD40* genes (Table S1). Further, the presence of WD40 domain was confirmed by SMART and Pfam searching. Both search outputs showed the presence of WD40 domain in all the 225 *SiWD40* genes. For convenience, the 225 *SiWD40* genes were named from SiWD001 to SiWD225 according to the order of their chromosomal locations.

Except for the presence of a conserved WD40 domain, the SiWD40 genes vary substantially in the size and sequences of their encoded proteins, and their physicochemical properties (Table S1). The location of the WD40 domain within the protein also differs. The length of SiWD40 proteins varied from 98 to 3518 amino acids. EXPASY analysis suggested that the SiWD40 protein sequences had large variations in isoelectric point (pI) values (ranging from 4.54 to 9.69) and molecular weight (ranging from10.866 kDa to 390.606 kDa; Table S1). The characteristic features of SiWD40 protein sequences were summarized in Table S1.

### Chromosomal Distribution and Structure of *SiWD40*

**Figure 1:**
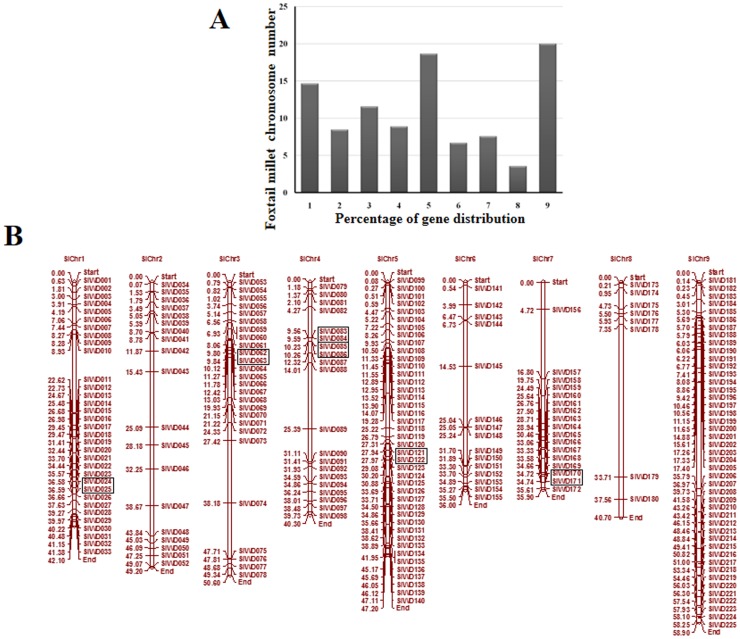
Distribution of 225 *SiWD40* genes onto nine foxtail millet chromosomes. (A) Percentage of *SiWD40* genes on each the foxtail millet chromosome to show their distribution abundance. (B) Graphical (scaled) representation of physical locations for each *SiWD40* gene on foxtail millet chromosomes (numbered 1–9). Tandem duplicated genes on a particular chromosome are depicted by black boxes. Chromosomal distances are given in Mb.

**Figure 2:**
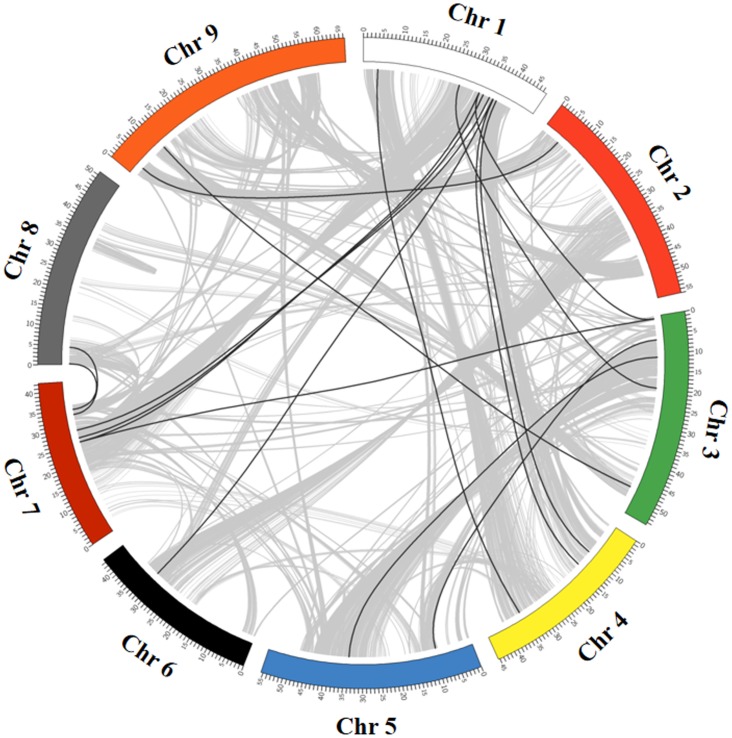
Distribution of segmentally duplicated *SiWD40* genes on foxtail millet chromosomes. Grey lines indicate collinear blocks in whole foxtail millet genome, and black lines indicate duplicated *SiWD40* gene pairs.

*In silico* mapping of *SiWD40*s on chromosomes indicated an uneven distribution of the genes on all the 9 chromosomes of foxtail millet (Figure 1). Among all, chromosome 9 contains the highest number of *SiWD40*s [45 (20%)], while lesser number genes were distributed on chromosome 8 [8 (∼3.5%)] (Figure 1). The exact position (in bp) of each *SiWD40* on foxtail millet chromosome is given in Table S1. Pattern of their distribution on individual chromosomes also revealed certain physical regions with a relatively higher accumulation of *SiWD40* gene clusters. For example, *SiWD40* genes located on chromosomes 3 and 7 appear to be congregate at the upper end and lower end of the arms, respectively (Figure 1). Recently, Zhang et al. [5] reported the occurrence of whole-genome duplication in foxtail millet similar to other grasses ∼70 million years ago (Mya). Hence, the presence of such large number of *SiWD40* genes in foxtail millet indicates the amplification of this gene family during the course of evolution. In all, 12 (∼5%) *SiWD40* genes were found to be tandem repeats with a maximum of six intervening genes separating the tandem repeats (Figure 1). The distance between these genes ranged from 6.2 kb to 32.2 kb. In the whole foxtail millet genome, 6688 (∼19%) genes are segmentally duplicated. Among the *SiWD40* genes, 32 (∼14%) were found to be segmentally duplicated (Figure 2).

Investigation of *SiWD40* gene structures revealed highly diverse distribution of intronic regions (from 0 to 29 in numbers) amid the exonic sequences, signifying considerable evolutionary changes that have occurred in the foxtail millet genome (Figure S1). The shortest *SiWD40* gene was merely 461 bp (*SiWD084*) whereas the longest one was identified as *SiWD006* with ∼ 23.5 kb genomic sequence (Table S1). This suggests that the evolution of these genes might have progressed immediately through some gene duplications or by integration into genomic region after reverse transcription [21], [32], [33].

### Phylogenetic Classification of SiWD40s and Identification of Domain Conservation

**Figure 3:**
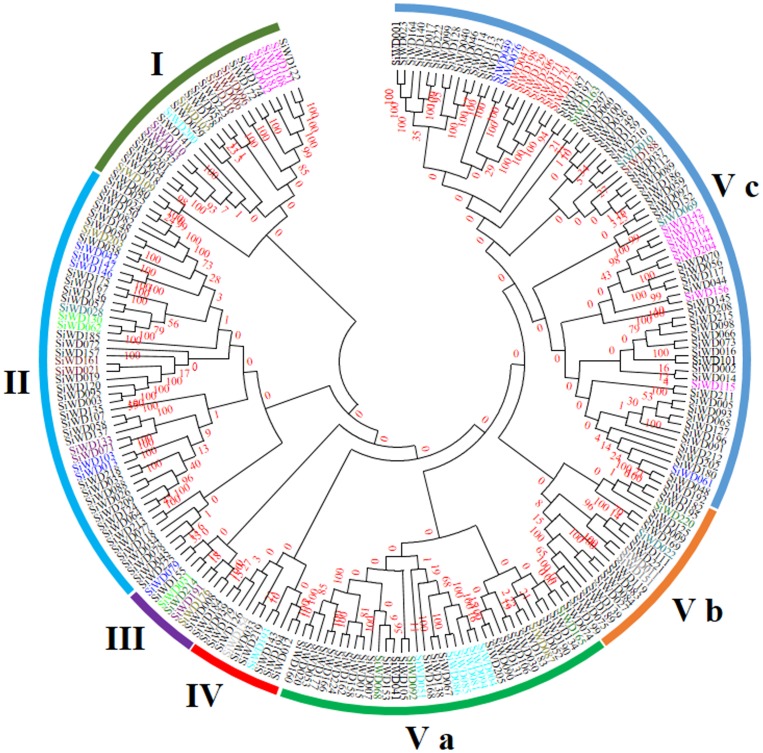
Phylogenetic relationships of foxtail millet WD40 proteins. The sequences were aligned by CLUSTALW at MEGA5 and the unrooted phylogenetic tree was deduced by neighbor-joining method with 1000 bootstrap replicates. The evolutionary distances were computed using p-distance method. The bootstrap values are shown at the nodes. The tree was divided into five phylogenetic cluster designated as I to V. The members of the SiWD40 were distinctly coloured to represent respective WD40 subfamilies.

A phylogenetic tree was constructed with 223 SiWD40 proteins by neighbour-joining (NJ) method. SiWD063 and SiWD216 being small sequences were excluded from alignment and phylogenetic tree construction. The phylogenetic analysis categorized all the SiWD40s into five discrete groups (Cluster I to V) comprising of 25, 48, 08, 11, and 131 proteins, respectively (Figure 3). Since a good number of the internal branches were observed to have high bootstrap values, it clearly shows the derivation of statistically reliable pairs of possible homologous proteins sharing similar functions from a common ancestor.

**Figure 4:**
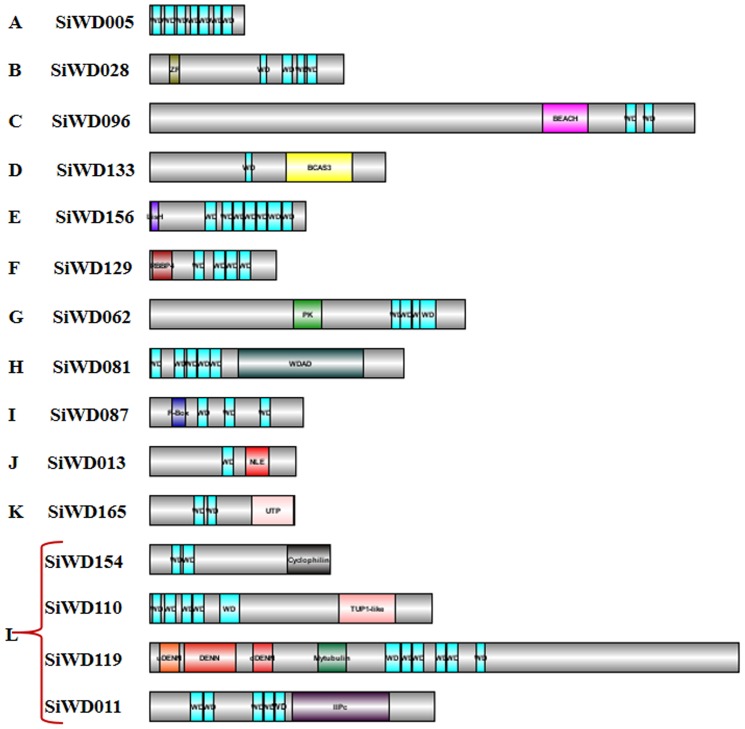
Structure of representative SiWD40 proteins from each subfamily. The protein structure is based on the presence of WD40 and other additional domains as identified by SMART and pfam. This categorizes the SiWD40 in 12 Subfamilies (A–L).

Further, the 225 SiWD40 proteins were classified into 12 subfamilies according to their domain compositions (Figure 4). About 146 members with only WD40 domain were categorized in subfamily A. Besides WD40 domain, SiWD40 proteins contained several other known functional domains and were classified into the following subfamilies. Four members containing the zinc finger domain were identified as subfamily B; Six members containing the Beige/BEACH domain were identified as subfamily C; Two members with breast carcinoma amplified sequence 3 (BCAS3) were identified as D subfamily; E subfamily (11 members) had LisH domain; F subfamily (7 members) had histone-binding protein RBBP4 or subunit C of CAF1 complex domains before WD40 repeats; G subfamily (3 members) had protein kinase domain or HEAT repeat; Eight members with the Coatomer WD associated region (WDAD) or Coatomer (COPI) alpha subunit C-terminus were identified as H subfamily; I subfamily (5 members) contained F-BOX and U-BOX; J subfamily (9 members) contained NLE (NUC) domain N terminal toWD40 repeats; Utp12, Utp13, Utp15, and Utp21; Six member of UTP containing domain were identified as subfamily K; L subfamily (21 members) contained other domains including TUP1-like,IIPc, DENN, Cyclophilin and domains with unknown function (Figure 4). The members of HBRBBP4 domain containing SiWD40 proteins are found in one cluster in the subgroup Vc (Figure 4). Interestingly, 97 out of 225 SiWD40 were identified as DWD proteins. Further, these 97 DWD proteins possess 116 DWD domains, of which 82 had one DWD domain, 11 had two domains and four had three domains. Thus, a diverse domain variation and conservations were evidenced and such conservation or variation between the proteins specifies the functional equivalence or diversification, respectively, with respect to the various aspects of biological functions [34].

### Gene Ontology Annotation

**Figure 5:**
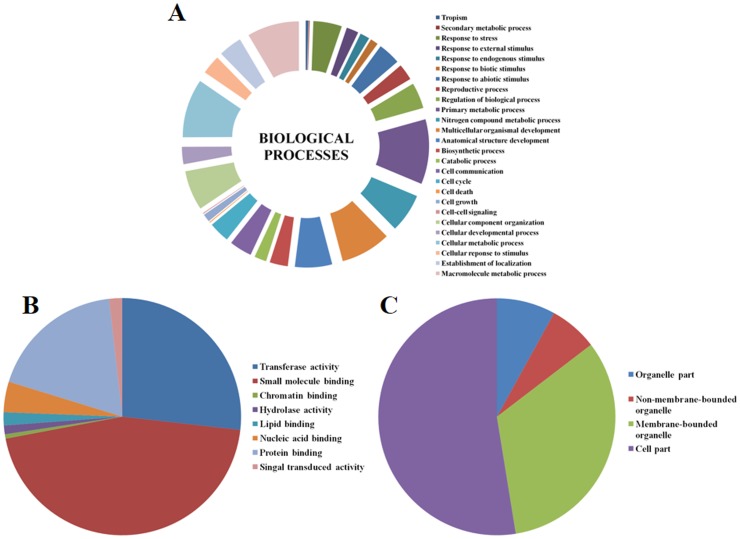
Gene Ontology (GO) distributions for the SiWD40 protein. The Blast2Go program defines the gene ontology under three categories, (A) biological processes, (B) molecular functions and (C) cellular component.

The GO slim analysis performed using Blast2GO showed the putative participation of SiWD40 proteins in diverse biological processes (Figure 5; Table S2). Out of 225 SiWD40 proteins, annotation could not be performed for 49 sequences and the results for the rest of 176 SiWD40s were defined in 26 categories of biological processes. The analysis showed that, predominant SiWD40 proteins were involved in response to primary metabolic process [75 (∼43%)], followed by cellular metabolic processes [68 (∼39%)]. Noteworthy, about 42 (∼24%) SiWD40 were evidenced to participate in response to stress stimulus. This highlights the putative association of SiWD40 proteins in stress tolerance behaviour of foxtail millet (Figure 5). In case of molecular functions, about 76 (∼43%) SiWD40 proteins were shown to participate in small molecule binding which concords with the molecular role of WD40 proteins in assisting protein-protein interactions. Cellular localization prediction showed that predominant [144 (∼82%)] SiWD40 proteins are localized in the cell part, of which 60 (∼42%) are nuclear localized (Figure 5; Table S2). This agrees with the experimental findings reported earlier [20], [35]. Further, Blast2GO was performed to draw a connection between the domain composition of the families/sub families and the functional classes, but there were no correlation observed.

### Promoter Analysis and miRNA Targets of *SiWD40* Genes

To support the functional predictions of the 42 stress-related *WD40* genes in foxtail millet, a comprehensive promoter analysis was performed. For this purpose, promoters and their regulatory elements were identified in DNA sequences (∼2 kb upstream of their putative start codons) using PlantPAN (Table S3). The analysis identified *cis*-acting regulatory elements (CARE) in the upstream DNA sequences that are involved in regulation of gene expression under stress conditions. The data might indicate a major role for the identified stress-related *WD40* genes in regulating their gene expression in response to different stresses in foxtail millet. Further, putative microRNAs (miRNA) targeting the *SiWD40* genes were also identified using psRNATarget server. It showed that about eight *SiWD40* genes were targeted by *Setaria italica* miRNAs (Table S4). These miRNAs identified in the present study would assist in deciphering the post-transcriptional control of gene regulation during physiological and stress-induced cellular responses.

### Orthologous Relationships of *WD40* Genes between Foxtail Millet and other Grass Species

To derive comparative mapping-based orthologous relationships of *SiWD40*, the physically mapped *WD40* genes were compared with those in the chromosomes of other related grass genomes namely, sorghum, maize and rice (Table 1; Figure S2). Of the identified 225 *SiWD40* protein-encoding genes in foxtail millet, the specific orthologous relationships could be derived on an average for ∼ 83.6% proteins. Maximum orthology of *SiWD40* genes annotated on the foxtail millet chromosomes was obtained with sorghum (86.2%) followed by rice (82.7%). The close evolutionary relationships would be the plausible reason for the extensive gene-level synteny shared between foxtail millet, sorghum and maize [5], [6], [21]. Interestingly, most of *SiWD40* genes revealed syntenic bias towards particular chromosomes of rice, maize and sorghum. For instance, the *SiWD40* genes on foxtail millet chromosome 1 showed 93% orthology and colinearity with sorghum chromosome 4 and rice chromosome 2 (90%) (Table 1; Figure S2). The *SiWD40* genes mapped on foxtail millet chromosome 9 showed inter-chromosomal inversions with rice chromosome 3 (72.7%) and maize chromosome 1 (65%), while colinearity with sorghum chromosome 1 (85.4%). Like-wise the *SiWD40* genes mapped on foxtail millet chromosome 5 revealed collinear relationships with rice chromosome 1 (82.5%) and sorghum chromosome 3 (92%) and inverted relationship with maize chromosome 3 (63.6%). The results indicated that the chromosomal rearrangements like duplication and inversion were predominant in shaping the distribution and organization of *WD40* genes in foxtail millet, rice, maize and sorghum genomes. The comparative mapping information provides a useful preface for understanding the evolutionary process of *WD40* genes among grasses involving the foxtail millet genome. Further, this study would be useful in selecting candidate *WD40* genes from foxtail millet and utilize them in genetic enhancement of other related grass family members.

**Table 1:** A summary of comparative mapping of foxtail millet *SiWD40* genes on sorghum, maize and rice.

*Setaria italica*	*Sorghum bicolor*	*Zea mays*	*Oryza sativa*
Chr1	Chr4 (92.85%)	Chr5 (50%), Chr4 (28.57%)	Chr2 (90%)
Chr2	Chr2 (83.33%)	Chr7 (75%)	Chr7 (58%), Chr9 (25%)
Chr3	Chr9 (50%)	Chr8 (30%), Chr6 (25%)	Chr5 (45%), Chr12 (20%)
Chr4	Chr10 (86.67%)	Chr9 (46.67%), Chr6 (26.67%)	Chr6 (80%)
Chr5	Chr3 (92%)	Chr3 (63.6%)	Chr1 (82.5%)
Chr6	Chr7 (84.61%)	Chr1 (28.57%), Chr4 (28.57%)	Chr8 (78.57%)
Chr7	Chr6 (50%), Chr8 (42.85%)	Chr10 (66.67%)	Chr4 (38.4%), Chr12 (23%)
Chr8	Chr5 (100%)	Chr3 (60%), Chr2 (40%)	Chr11 (62.5%)
Chr9	Chr1 (85.36%)	Chr1 (65%)	Chr3 (72.7%)

### Duplication and Divergence Rate of the *SiWD40* Genes

**Figure 6:**
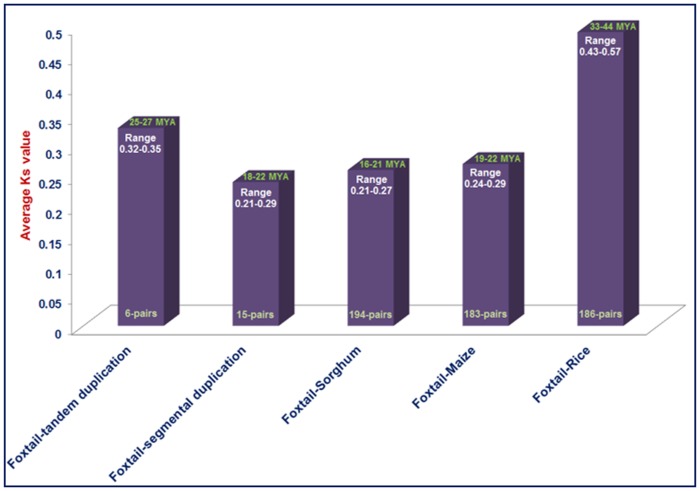
Time of duplication and divergence (MYA) based on synonymous substitution rate (Ks) estimated using duplicated *SiWD40* gene pairs of foxtail millet and orthologous *SiWD40* gene pairs between foxtail millet and rice or maize or sorghum.

Multiple copies of genes in a gene family possibly evolve due to evolutionary events like whole genome tandem and segmental duplications. Such gene duplication has been documented in several plant transcription factor (TF) gene families such as MYB, F-box as well as in NAC [21], [36], [37]. We thus explored the effect of Darwinian positive selection in duplication and divergence of *WD40* genes. To interpret this, the ratios of non-synonymous (Ka) versus synonymous (Ks) substitution rate (Ka/Ks) were estimated for six tandem and 15 segmentally duplicated gene-pairs as well as between orthologous gene-pairs of *SiWD40* with those of rice (186-pairs), maize (183) and sorghum (194). The ratios of Ka/Ks for tandemly duplicated gene-pairs ranged from 0.09 to 0.15 with an average of 0.12 (Table S5), whereas Ka/Ks for segmentally duplicated gene-pairs ranging from 0.11 to 0.20 with an average of 0.13 (Table S6). It suggested that the duplicated *SiWD40* genes are under strong purifying selection pressure since their Ka/Ks ratios estimated as <1. Additionally, the duplication event of these tandemly and segmentally duplicated genes may be estimated to have occurred around 25–27 and 18–22 Mya, respectively (Figure 6). Among the orthologous gene-pairs of *SiWD40* with those of other grass species, the average Ka/Ks value was maximum between rice and foxtail millet (0.55) and least for sorghum-foxtail millet gene-pairs (0.23; Table S7). The relatively higher rate of synonymous substitution between rice and foxtail millet *WD40* genes indicated their earlier divergence around 33–44 Mya from foxtail millet as compared to sorghum and maize *WD40* genes (Figure 6). Remarkably, the *WD40* gene-pairs between sorghum and foxtail millet (average Ka/Ks = 0.23) appear to have undergone extensive intense purifying selection in comparison to foxtail millet-maize (Ka/Ks = 0.30) and foxtail millet-rice (Ka/Ks = 0.55) *WD40* genes (Table S7). This conforms to their recent time of divergence around 16–21 Mya. The estimation of tandem and segmental duplication time (average of 22 Mya) of foxtail millet *WD40* genes in between the divergence time of foxtail millet-rice (37.7 Mya) and foxtail millet-maize (20.8 Mya) and foxtail millet-sorghum (19.2 Mya) orthologous *WD40* gene-pairs are comparable to evolutionary studies involving the protein-coding genes annotated from the recently released draft genome sequences of foxtail millet [5]. Interestingly, the *SiWD40* gene-pairs showing segmental and tandem duplication events are under similar evolutionary pressure (Ka/Ks = 0.12) of which, the segmentally duplicated genes revealed much recent duplication events (average 18.5 Mya) in contrast to tandemly duplicated gene-pairs (average 25.4 Mya) and orthologous foxtail millet-sorghum gene-pairs (19.2 Mya). It overall suggests that the segmental and tandem duplication events including the divergence events of *SiWD40* genes from other grass species have played a predominant role in evolution for shaping such gene family in foxtail millet.

### *In silico* Tissue-specific Expression Profiling of *SiWD40*

Heat map generated for examining the tissue-specific expression showed a differential transcript abundance of 225 *SiWD40* genes in 4 major tissues namely root, leaf, stem and spica (Figure S3). About 87 genes (∼39%) showed higher expression in all the four tissues and conversely, 37 (∼16%) were found to be low expressed in all the four tissues (Figure S3). Comparing the expression of all the 225 *SiWD40* showed a relatively higher expression of SiWD024 and SiWD065 in all the tissues. Some of the *SiWD40s* also showed tissue-specific expression, such as *SiWD158* expressed only in root, *SiWD063* in leaf, and *SiWD023, SiWD108* and *SiWD162* express specifically in spica. The tissue-specific expression profiling of *SiWD40s* would facilitate the combinatorial usage of *SiWD40s* in transcriptional regulation of different tissues, whereas ubiquitously expressed *SiWD40s* might regulate the transcription of a broad set of genes. This heatmap data also enables the overexpression studies of *SiWD40s* across the tissues to impart stress tolerance in both foxtail millet and related crop species.

### *SiWD40* Expression Profiles of during Abiotic Stresses and Homology Modeling

**Figure 7:**
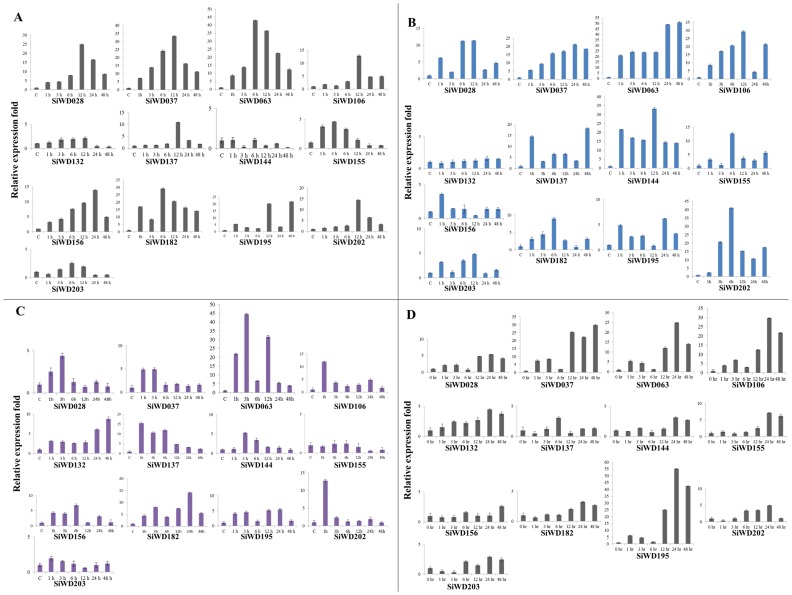
The relative expression ratio of 13 candidate *SiWD40* genes analyzed using qRT-PCR under (A) dehydration stress, (B) salinity stress, (C) ABA treatment (D) Cold stress for 0, 1, 3, 6, 12, 24 and 48 h. The relative expression ratio of each gene was calculated relative to its expression in control sample (0 h). *Act2* was used as an internal control to normalize the data. The error bars representing standard deviation were calculated based on three technical replicates for each biological duplicates.

Gene expression patterns can offer crucial indications for determining the gene function. Considering the potential abiotic stress tolerance characteristic of foxtail millet, we studied the expression pattern of *WD40* genes during dehydration, salinity, abscisic acid (ABA) and cold stress. About 13 candidate genes were chosen for quantitative expression analysis based on the GO annotation (possessing roles in abiotic stress stimuli) and representing all the sub-families. The expression pattern of the candidate genes in response to dehydration, salinity, ABA and cold stress during 0, 1, 3, 6, 12, 24 and 48 h durations of treatments was examined (Figure 7A–D). In summary, qRT-PCR analyses showed that all the candidate *SiWD40* genes have incurred variations in their expression patterns in response to one or more stresses in course of the experimentations. Higher expression of *SiWD40* genes were evidenced at 12^th^ hr during dehydration stress and at 6^th^ hr during salinity stress (Figure 7A-6B). During ABA treatment, higher number of genes was evidenced to be expressed at 3^rd^ hr (Figure 7C) while higher expressions of *SiWD40* genes was observed at 24^th^ hr during cold stress (Figure 7D). Noteworthy, *SiWD063* was found to be highly expressed in all the four stresses. Further, *SiWD028, SiWD037, SiWD063* and *SiWD182* were found to be highly expressed during dehydration stress, whereas *SiWD63, SiWD106*, *SiWD144* and *SiWD202* were upregulated during salinity stress. In ABA stress, *SiWD063* and *SiWD182* were evidenced to be highly expressed. Cold stress showed higher expression of *SiWD37, SiWD63* and *SiWD195*. This variability in gene expression patterns implies that *SiWD40*s may regulate a complex network of pathways to perform different physiological functions for acclimatizing towards multiple challenges. Since no reports were available on the study of WD40 expression patterns during stress, this comprehensive expression profile would invoke investigations on the role of WD40 in imparting stress tolerance.

**Figure 8:**
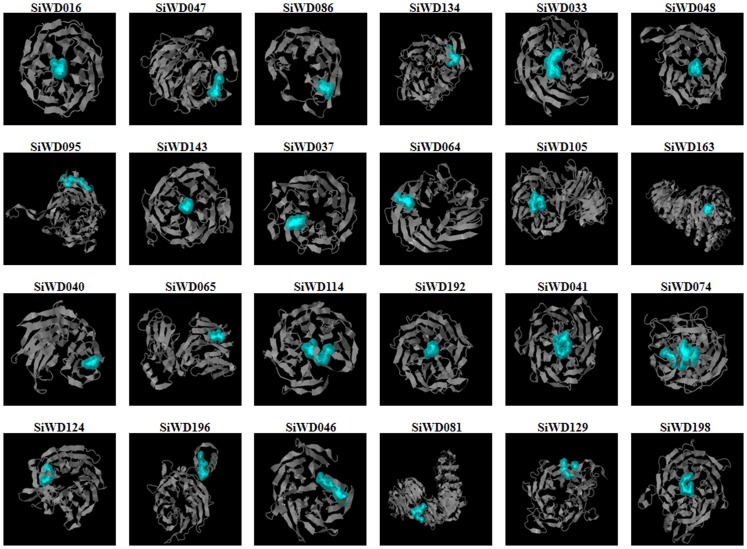
Predicated structures of SiWD40 proteins. The structure of 24 SiWD40 proteins with >90% confidence level were shown. Active sites are highlighted in blue colour.

Three dimensional protein models were constructed by sequence similarity searching the PDB database using BLASTP. Twenty four proteins having higher homology were selected and Phyre2 was used to predict the homology modeling (Figure 8). Noticeably, these 24 proteins represent diverse WD40s, in terms of repeats and domains (Table S9). Phyre2 uses the alignment of hidden Markov models via HMM-HMM search [38] to significantly improve the accuracy of alignment and detection rate. The intensive mode of Phyre 2 uses the multi-template modeling for higher accuracy. Furthermore it integrates a new *ab initio* folding simulation termed as Poing [39] to model regions of proteins with no noticeable homology to known structures. The protein structure of all the 24 SiWD40 are modelled at >90% confidence and the percentage residue varied from 81 to 100 (Figure 8, Dataset S1). The secondary structure predominantly comprised of *β* - sheets and coils, with rare occurrence of α - helices (Figure 8). Hence all the predicted protein structures are considered highly reliable and this offers a preliminary basis for understanding the molecular function of SiWD40 proteins.

## Conclusions

The WD-repeat proteins possess seven WD40-repeat motifs, with the conserved core of the repeat containing 44 to 60 residues that terminates with Trp and Asp. The repeats form a β - propeller fold, allowing formation of a highly stable structure that coordinates the interactions with several other proteins [40]. Hence, its role is deemed imperative in protein-protein interactions and our recent identification on the role of WD40 proteins in abiotic stress tolerance in foxtail millet [20] had motivated us to conduct a genome-wide survey in this model crop. In summary, a total of 225 *SiWD40* genes were found to be present in foxtail millet genome. The variations in the lengths and genomic structure of SiWD40s support the great deal of complexity that has evolved within this gene family. Noteworthy, the *SiWD40* genes shared high orthology with their counter-parts in sorghum and maize supporting their close evolutionary relationship. Further, for the first time, we had showed a preliminary expression profiling of some *SiWD40* genes influenced by several environmental stimuli, including dehydration, salinity, ABA treatment and cold stress. We have also described the structure of 24 SiWD40 proteins which would expedite the investigation of its molecular functions. Hence, this report would be useful for the millet research community in selecting candidate genes for functional studies of WD40 members in foxtail millet, and other millets and bioenergy grasses.

## Materials and Methods

### Retrieval and Identification of WD40 Genes in *Setaria italica*

The Hidden Markov Model (HMM) profile of the WD40 domain (PF00400) retrieved from Pfam v27.0 (http://Pfam.sanger.ac.uk/) was queried against the PHYTOZOME v8.0 database (www.phytozome.net/) of *Setaria italica*. All hits with expected values less than 1.0 were retrieved and redundant sequences were removed using BLASTclust v2.17 (http://toolkit.tuebingen.mpg.de/blastclust). Each non-redundant sequence was checked manually for the presence of the conserved WD40 domain by executing SMART (http://smart.embl-heidelberg.de/) [41] and Pfam searches.

### Physical Mapping, Gene Structure Prediction and Estimation of Genomic Distribution

Physical mapping of the genes encoding *SiWD40* onto the foxtail millet genome was performed by conducting BLASTP search of respective sequences against the PHYTOZOME database using default settings. Subsequently the genes were plotted onto the nine chromosomes according to their ascending order of physical position (bp), from the short arm telomere to the long arm telomere and ultimately the map was displayed using MapChart [42]. Since tandem and segmental duplication events that have occurred in the genome would plausibly result in the expansion of gene family, we investigated the mechanisms involved in the expansion of WD40 members in foxtail millet. The method of Plant Genome Duplication Database was used to identify segmental duplications [43]. Precisely, BLASTP search was performed against the complete peptide sequences of *Setaria italica* and the first 5 matches with E-value <1e-05 were identified as potential anchors. Collinear blocks were evaluated by MCScan v0.8 and alignments with an E value <1e-5 were considered as significant matches [44], [45]. The segmental duplication was finally visualized using Circos 0.55 (http://circos.ca) [46]. Tandem duplications were characterized as adjacent genes of same sub-family located within the same or neighbouring intergenic region [45]. The exon-intron positioning of the genes were determined using Gene structure display server (gsds.cbi.pku.edu.cn/) [47] by comparing the full-length cDNA or predicted coding sequence (CDS) of *SiWD40* with their corresponding genomic sequence.

### Phylogenetic Analysis and Gene Ontology (GO) Annotation

The amino acid sequences of SiWD40 were imported into MEGA5 [48] and multiple sequence alignments were performed using ClustalW with a gap open penalty of 10 and a gap extension penalty of 0.1 [49]. The alignment file was then subjected to create an unrooted phylogenetic tree based on the neighbor-joining method [50] and after bootstrap analysis for 1000 replicates, the final tree was generated. The functional annotation of SiWD40 sequences and the analysis of annotation data were performed using Blast2GO (http://www.blast2go.com) [51]. The amino acid sequences of SiWD40 were imported into Blast2GO program to execute three steps viz, (i) BLASTp against the non-redundant protein database of NCBI, (ii) mapping and retrieval of GO terms associated with the BLAST results, and (iii) annotation of GO terms associated with each query to relate the sequences to known protein function. The program provides the output defining three categories of GO classification namely biological processes, cellular components and molecular functions.

### Analysis of Promoter and miRNA Targets

The upstream sequences (∼2000 bp) of each identified *SiWD40* gene were retrieved from the PHYTOZOME (http://phytozome.net/). The upstream sequences were analyzed for the identification of regulatory cis-elements important for gene expression under stress conditions using PlantPAN [52]. Further, from our database of *Setaria italica* miRNAs (unpublished data) putative miRNAs targeting the SiWD40 genes were identified using psRNATarget [53].

### Comparative Physical Mapping of SiWD40 Proteins between *S. italica* and other Grass Species

The amino acid sequences of physically mapped SiWD40 protein-encoding genes spanning the nine foxtail millet chromosomes were BLASTP searched against peptide sequences of sorghum, maize and rice (http://gramene.org/; www.phytozome.net) to infer orthologous relationship among the chromosomes of foxtail millet and the other three grass species. Reciprocal BLAST has also been performed to ensure the unique relationship between the orthologous genes. BLAST hits with E-value ≤1e-5 and at least 80% homology were considered significant. The comparative orthologous relationships of *WD40* genes among foxtail millet, rice, sorghum and maize chromosomes were finally visualized using MapChart [42].

### Estimation of Synonymous and Non-synonymous Substitution Rates

The amino acid sequences duplicated protein-encoding WD40 genes as well as orthologous gene-pairs between foxtail millet and rice, maize and sorghum were aligned using ClustalW based multiple sequence alignment tool. The CODEML program in PAML interface tool of PAL2NAL (http://www.bork.embl.de/pal2nal/) [54], was used to estimate the synonymous (Ks) and non-synonymous (Ka) substitution rates by aligning the amino acid sequences and their respective original cDNA sequences of *SiWD40* genes. Time (million years ago, Mya) of duplication and divergence of each *SiWD40* genes were estimated using a synonymous mutation rate of λ substitutions per synonymous site per year, as T = Ks/2λ (λ = 6.5×10^−9^) [55], [56].

### Expression Profiling using RNA-seq Data

To elucidate the tissue-specific expression profile of SiWD40 genes, the *Setaria italica* Illumina RNA-HiSeq reads from 4 tissues namely spica, stem, leaf and root were retrieved from European Nucleotide Archive [SRX128226 (spica); SRX128225 (stem); SRX128224 (leaf); SRX128223 (root)] [57]. The RNA-seq data was then filtered by NGS toolkit [58] to remove low quality reads and was mapped onto the gene sequences of *Setaria italica* by CLC Genomics Workbench v.4.7.1 (http://www.clcbio.com/genomics). The number of reads mapped was normalized by RPKM (reads per kilobase per million) method. The heat map showing tissue specific expression was generated on the RPKM value for each gene in all the tissue samples using TIGR MultiExperiment Viewer (MeV4) software package [59], [60].

### Plant Materials and Stress Treatments

Seeds of foxtail millet cv. Prasad known for its abiotic stress tolerance were procured from National Bureau of Plant Genetic Resources (NBPGR), Hyderabad, India and grown in a plant growth chamber (PGC-6L; Percival Scientific Inc., USA) at 28±1°C day/23±1°C night with 70±5% relative humidity and photoperiod of 14 h. For stress treatments, 21-day-old seedlings were exposed to 250 mM NaCl (salinity), 20% PEG 6000 (dehydration), 150 µM abscisic acid (ABA) and incubation at 4°C (cold) for 1 h, 3 h, 6 h, 12 h, 24 h and 48 h. Unstressed plants were maintained as controls. After the treatments, seedlings were immediately frozen in liquid nitrogen and stored at −80°C until RNA isolation. The above experiments were repeated thrice to ensure precision and reproducibility.

### RNA Extraction and Quantitative Real-time PCR Analysis

Total RNA was isolated by following the procedure described by Longeman et al. [61] and treated with RNase-free DNase I (50 U/µl; Fermentas, USA) for removing DNA contamination. The quality and purity of the preparations were determined at OD_260_:OD_280_ nm absorption ratio (1.8–2.0) and the integrity of the preparations was determined by resolving in 1.2% agarose gel containing formaldehyde. About 1 µg total RNA was reverse transcribed to first strand cDNA using random primers by Protoscript M-MuLV RT (New England Biolabs, USA) following manufacturer’s instructions [21]. The qRT-PCR primers were designed using Primer Express 3.0 software (PE Applied Biosystems, USA) with default parameters (Table S8). qRT-PCR was carried out in three technical replicate for each biological duplicate by one step real time PCR system of Applied Biosytems (USA). The PCR mixtures and reactions were used as described previously by Kumar et al.^21^ Melting curve analysis (60 to 95°C after 40 cycles) and agarose gel electrophoresis were performed to check amplification specificity for absence of multiple amplicons or primer dimers [22]. A constitutive *Act2* gene-based primer was used as endogenous control. The amount of transcript accumulated for *SiWD40* genes normalized to the internal control *Act2* were analyzed using 2^−ΔΔCt^ method cDNA synthesis. The PCR efficiency which is dependent on the assay, performance of the master mix and quality of sample, was calculated as: Efficiency = 10 ^(−1/slope)^ − 1 by the software itself (Applied Biosystems).

### Homology Modeling of SiWD40 Proteins

All the SiWD40 proteins were searched against the Protein Data Bank (PDB) [62] by BLASTP (with the default parameters) to identify the best template having similar sequence and known three-dimensional structure (Table S9). The data was fed in Phyre2 (Protein Homology/AnalogY Recognition Engine; http://www.sbg.bio.ic.ac.uk/phyre2) for predicting the protein structure by homology modeling under ‘intensive’ mode [63]. For active site prediction, the PDB code was submitted to Q-SiteFinder [64].

## Supporting Information

Figure S1 Gene structures of 225 *SiWD40* proteins. Exons and introns are represented by green boxes and black lines, respectively.
(TIF)

Figure S2 Comparative physical mapping revealed high degree of orthologous relationships of *SiWD40* genes located on nine chromosomes of foxtail millet with (A) sorghum, (B) maize and (C) rice.
(JPG)

Figure S3 Heat map representation of *SiWD40* genes across different tissues. The Illumina RNA-seq data were re-analyzed and the heat map was generated. Bar at the top represents log_2_ transformed values, thereby values −2.0, 2.0 and 4.0 represent low, intermediate and high expression, respectively.
(TIF)

Table S1 A catalog of 225 *Setaria italica* WD40 proteins.
(XLS)

Table S2 Blast2GO annotation details of SiWD40 protein sequences.
(XLS)

Table S3 Characteristics of the promoter region of 42 stress-related *SiWD40* genes.
(DOC)

Table S4 Summary of putative miRNA targeting the *SiWD40* genes.
(DOC)

Table S5 The Ka/Ks ratios and estimated divergence time for tandemly duplicated SiWD40 proteins.
(DOC)

Table S6 The Ka/Ks ratios and estimated divergence time for segmentally duplicated SiWD40 proteins.
(DOCX)

Table S7 The Ka/Ks ratios and estimated divergence time for orthologous WD proteins between foxtail millet, rice, sorghum and maize.
(DOC)

Table S8 List of primers used in quantitative real time-PCR expression analysis of 13 SiWD40 genes.
(DOC)

Table S9 Characteristics of 24 candidate SiWD40 proteins chosen for homology modeling.
(DOC)

Dataset S1 Compilation of PDB files used for homology modeling of SiWD40 proteins.
(RAR)
