# Supplementary figures and images for: Genome-Wide Investigation and Expression Analyses of WD40 Protein Family in the Model Plant Foxtail Millet (Setaria italica L.)
Source: PLoS One. 2014 Jan 23;9(1):e86852. doi: 10.1371/journal.pone.0086852 (PMC3900672; doi:10.1371/journal.pone.0086852)

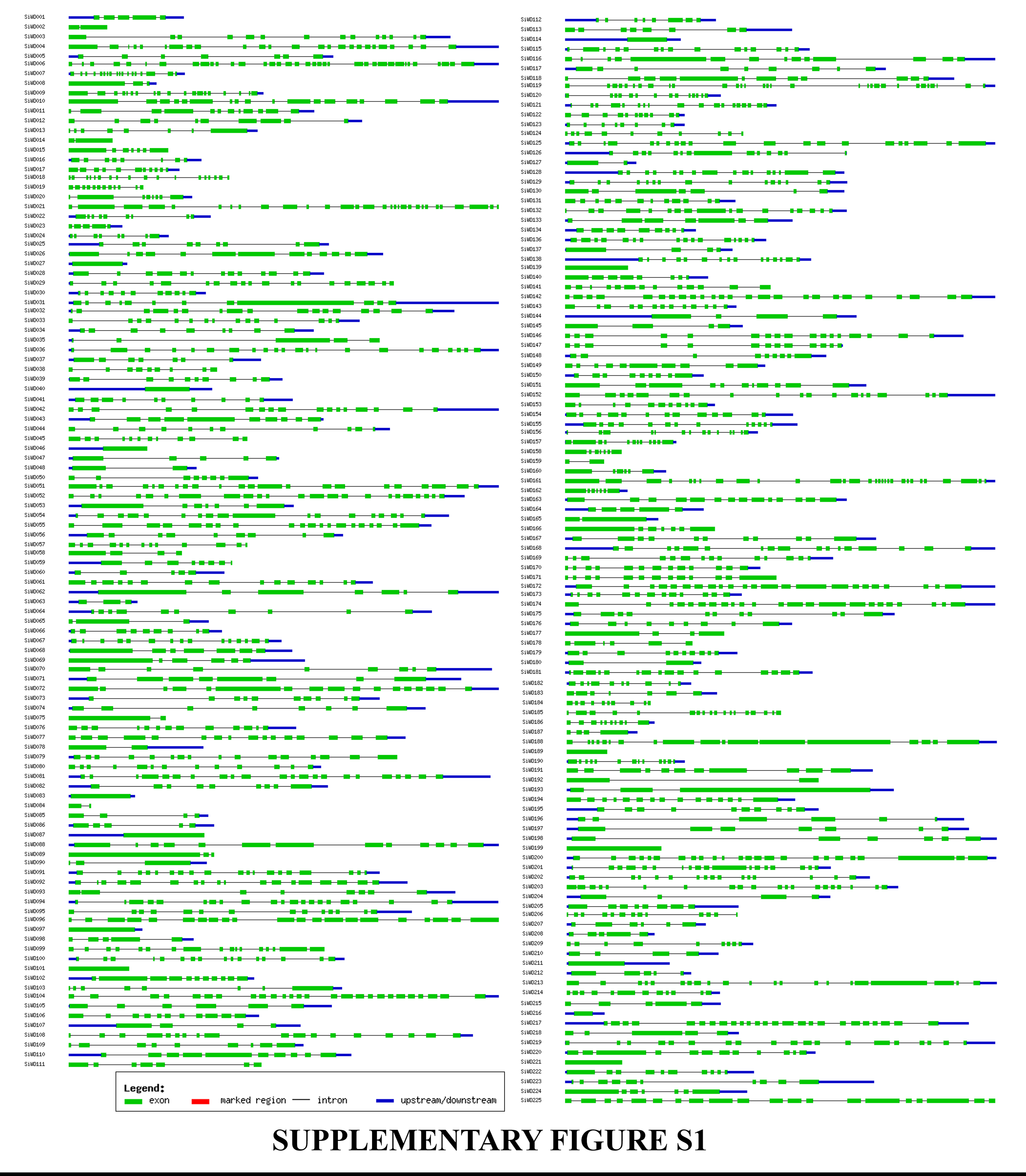

Supplement: Figure S1 — Gene structures of 225 SiWD40 proteins. Exons and introns are represented by green boxes and black lines, respectively. (TIF) [file pone.0086852.s001.tif]

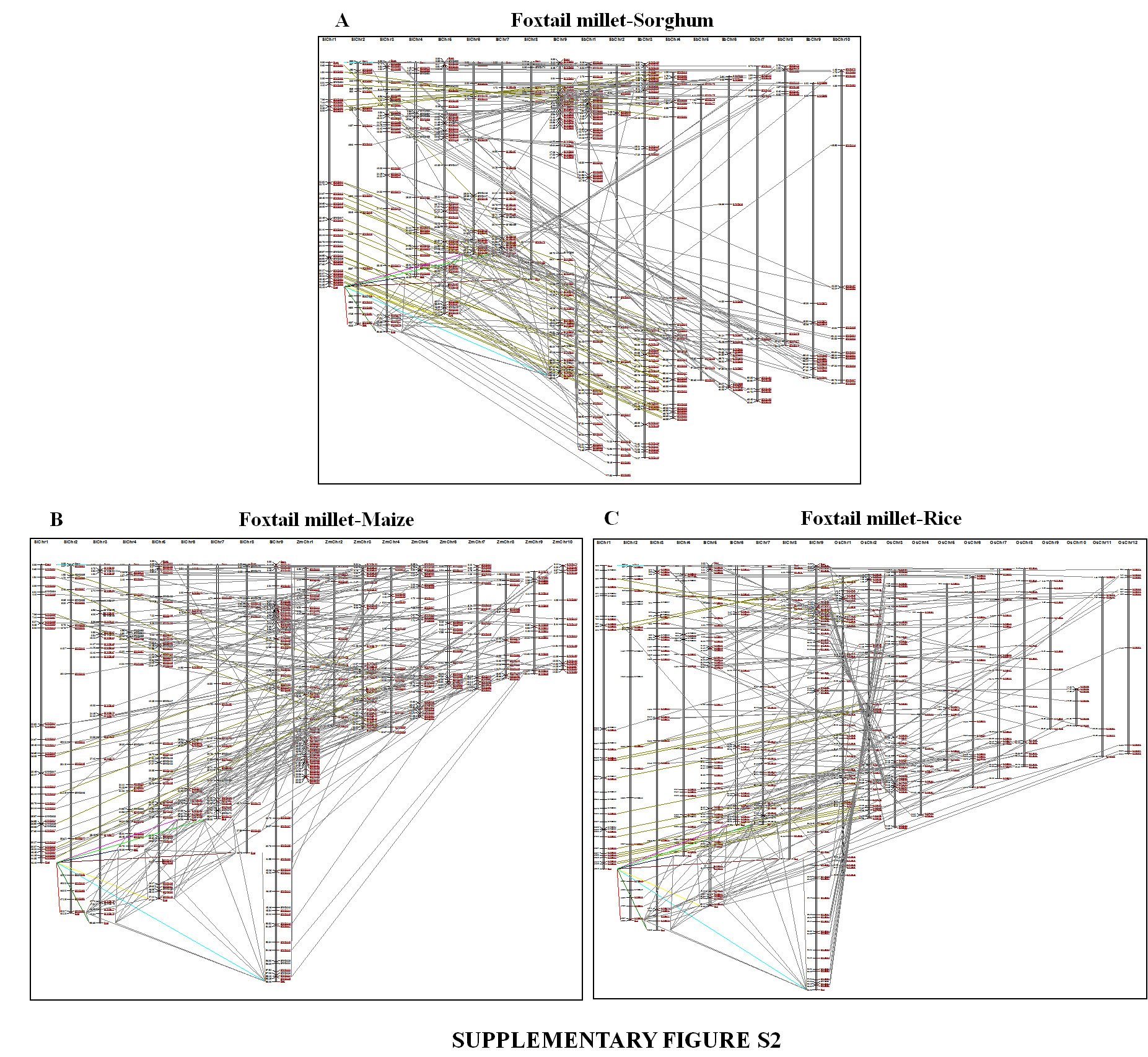

Supplement: Figure S2 — Comparative physical mapping revealed high degree of orthologous relationships of SiWD40 genes located on nine chromosomes of foxtail millet with (A) sorghum, (B) maize and (C) rice. (JPG) [file pone.0086852.s002.jpg]

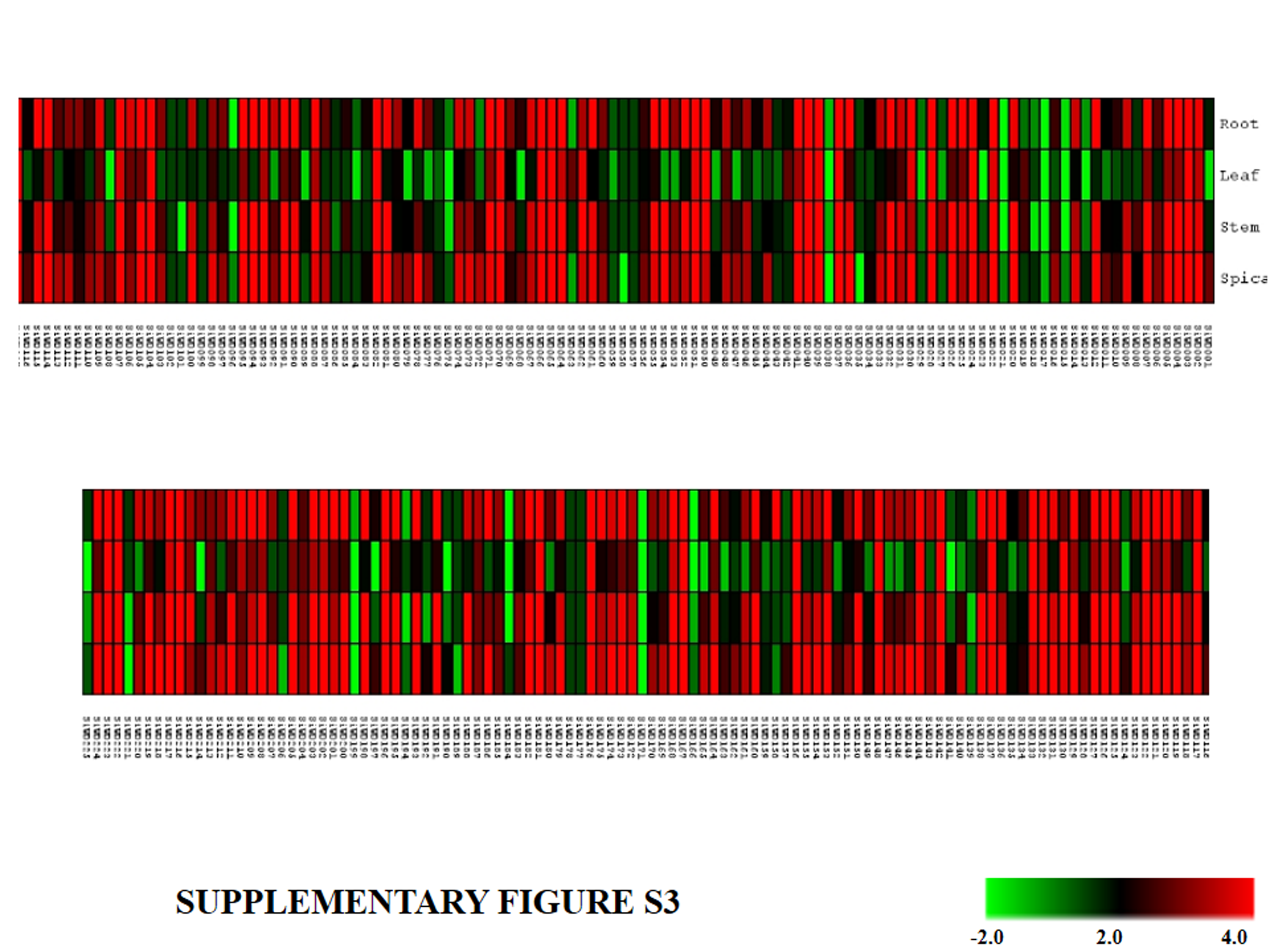

Supplement: Figure S3 — Heat map representation of SiWD40 genes across different tissues. The Illumina RNA-seq data were re-analyzed and the heat map was generated. Bar at the top represents log2 transformed values, thereby values −2.0, 2.0 and 4.0 represent low, intermediate and high expression, respectively. (TIF) [file pone.0086852.s003.tif]
