# Supplementary material for: Genome-Wide Investigation and Expression Analyses of WD40 Protein Family in the Model Plant Foxtail Millet (Setaria italica L.)
Source: PLoS One. 2014 Jan 23;9(1):e86852. doi: 10.1371/journal.pone.0086852 (PMC3900672; doi:10.1371/journal.pone.0086852)
Supplement: Table S3 — Characteristics of the promoter region of 42 stress-related SiWD40 genes. (DOC) [file pone.0086852.s006.doc]

**Table S3.** Characteristics of the promoter region of 42 stress-related *SiWD40* genes.

| **Gene name** | ***Cis*-acting distribution and position from ATG** | | | | | |
| --- | --- | --- | --- | --- | --- | --- |
|  | **DRE/CRT Core** | **CRTDRE**  **HvCBF2** | **ABRE** | **MYC**  **core** | **MYB**  **core** | **LTRE Core** |
| SiWD007 | 220 | NA | (-162) | 855,(-1465) | 1134,1437 | 205 |
| SiWD014 | (-871) | 129 | 467 | NA | NA | 249,787 |
| SiWD028 | (-742),(-859) | NA | NA | NA | NA | NA |
| SiWD037 | 1060,120,(-10043) | NA | NA | NA | NA | NA |
| SiWD038 | NA | NA | NA | NA | NA | NA |
| SiWD039 | 1773 | NA | NA | NA | NA | 487 |
| SiWD041 | 155,794 | NA | 1587, | NA | NA | NA |
| SiWD055 | 291,339,1485, (-535) | NA | (-468),(-136) | NA | NA | NA |
| SiWD058 | NA | NA | 309 | NA | NA | 128 |
| SiWD063 | NA | 1962 | NA | NA | NA | NA |
| SiWD066 | NA | 998,-1001 | NA | 1639 | (-121) | NA |
| SiWD071 | (-301) | NA | -899 | NA | (-1229) | NA |
| SiWD087 | (-457) | NA | -237 | NA | NA | NA |
| SiWD0105 | 1042,(-1787) | (-1748) | NA | NA | NA | 1167 |
| SiWD106 | NA | NA | NA | NA | NA | 339 |
| SiWD107 | 1718,-1922 | NA | 2415 | NA | NA | NA |
| SiWD123 | NA | 1997 | NA | NA | 907 | NA |
| SiWD129 | NA | 510,(-687) | 890,1422 | NA | 1476 | NA |
| SiWD130 | 1031,(-496) | NA | 1295,(-1217) | NA | 772 | 1031 |
| SiWD132 | (-1170) | 99 | 1190,(-950),1188 |  | 1261 | NA |
| SiWD134 | NA | 1155 | 366 |  | 1647 | (-1600) |
| SiWD137 | 712 | NA | 916 | NA | 1827 | NA |
| SiWD140 | NA | NA | -639,433 |  |  | NA |
| SiWD141 | 1475 | NA | (-1319) |  | 130 | NA |
| SiWD143 | NA | 1689 | 1777 | 894 | 1908 | NA |
| SiWD144 | (-1794) | NA | (-2505) | (-887) | -3250 | NA |
| SiWD151 | 1497,1787,1585, (-1524) | -1985 | NA | NA | (-1158) | 1368,(-257),(-1465) |
| SiWD154 | -1788 | 1580 | (-1444),(-1654) |  | 666,1540 | 1828 |
| SiWD155 | NA | NA |  | (-756) | (-533),593 | NA |
| SiWD156 | 147,620 | NA | 422 | NA | (-1164) | 172,620 |
| SiWD158 | (-1378),(-1797) | NA | 207 | NA | (-734) | (-1727) |
| SiWD163 | 179,935,1215,(-743) | NA | (-1213) | NA | 1130 | (-1155) |
| SiWD182 | NA | NA | 1852 | NA |  | NA |
| SiWD184 | 185,(-344),(-1190) | 962,1027 | (-712) | NA | 1979,(-992) |  |
| SiWD195 | (-2176) | NA | NA | NA |  | 1710,2446 |
| SiWD202 | 391 | 115,1682 | NA | NA | (-266) | 555 |
| SiWD203 | (-479),(-1550) | 1814 | 1914 | NA |  | 766 |
| SiWD205 | NA | NA | NA | NA | 1984,(-087) | (-397) |
| SiWD210 | 891,1495,(-761),(-785) | NA | NA | NA | NA | NA |
| SiWD212 | (-592) | (-2119) | NA | NA | NA | NA |
| SiWD215 | NA | NA | 338,1565 | NA | NA | NA |
| SiWD219 | NA | NA | NA | NA | 184,252,292 | 227,1500,1620 |

(-) indicates *cis*-elements found on the complementary DNA strand from ATG

NA= Not available
