# Supplementary material for: Genome-Wide Investigation and Expression Analyses of WD40 Protein Family in the Model Plant Foxtail Millet (Setaria italica L.)
Source: PLoS One. 2014 Jan 23;9(1):e86852. doi: 10.1371/journal.pone.0086852 (PMC3900672; doi:10.1371/journal.pone.0086852)
Supplement: Table S4 — Summary of putative miRNA targeting the SiWD40 genes. (DOC) [file pone.0086852.s007.doc]

**Table S4.** Summary of putative miRNAs targeting the *SiWD40* genes.

| **WD40 ID** | **Phytozome ID** | **Target**  **miRNA ID** | **E-Value** | **miRNA**  **start** | **miRNA**  **end** | **Target**  **start** | **Target**  **end** | **miRNA_aligned_fragment** | **Target_aligned_fragment** | **Inhibition** |
| --- | --- | --- | --- | --- | --- | --- | --- | --- | --- | --- |
| SiWD109 | Si001046m | Sit-miR1439-a | 2.5 | 1 | 20 | 23 | 42 | AUUUGGAAUGGAGGGAGUAU | UUCCUCCCUCCGUUCCAAAU | Cleavage |
| SiWD142 | Si013145m | Sit-miR5568.58 | 3 | 1 | 20 | 2231 | 2250 | UUUCUAGGUUUAUAUCUUUU | AGAAGAUGAAAACCUGGAAG | Cleavage |
| SiWD031 | Si016093m | Sit-miR5568.84 | 3 | 1 | 21 | 970 | 990 | GUUGUUUUGGGUUUUCUAAAU | GUUGAGAAGACUCAAGACAAU | Cleavage |
| SiWD017 | Si017288m | Sit-miR5568.4 | 2.5 | 1 | 20 | 655 | 674 | GUUGUUUUGAUUUUUCUAGA | UCUAAAAGAAUCAGAACAAU | Cleavage |
| SiWD054 | Si021140m | Sit-miR5568.125 | 2.5 | 1 | 21 | 2022 | 2042 | UUUCUAGAUACAUAAUUUUUG | UGAAAAUUAUGUUUCUAGAAU | Translation |
| SiWD054 | Si021140m | Sit-miR5568.138 | 3 | 1 | 21 | 2022 | 2042 | UUUCUAGGUACAUAAUUUUUG | UGAAAAUUAUGUUUCUAGAAU | Translation |
| SiWD054 | Si021140m | Sit-miR5568.85 | 2.5 | 1 | 21 | 2022 | 2042 | UUUCUAGAUACAUAAUUUUUG | UGAAAAUUAUGUUUCUAGAAU | Translation |
| SiWD181 | Si034443m | Sit-miR5169-e | 3 | 1 | 20 | 1656 | 1675 | UUUGACCAAUUUUAUAGAAA | CUUCUGGAAAGUUGGUCAAA | Cleavage |
