# Supplementary material for: Genome-Wide Investigation and Expression Analyses of WD40 Protein Family in the Model Plant Foxtail Millet (Setaria italica L.)
Source: PLoS One. 2014 Jan 23;9(1):e86852. doi: 10.1371/journal.pone.0086852 (PMC3900672; doi:10.1371/journal.pone.0086852)
Supplement: Table S5 — The Ka/Ks ratios and estimated divergence time for tandemly duplicated SiWD40 proteins. (DOC) [file pone.0086852.s008.doc]

**Table S5.** The Ka/Ks ratios and estimated divergence time for tandemly duplicated SiWD40 proteins.

| **Group** | **Gene** | **Duplicate** | **e value** | **%Homology** | **Genes intervening** | **Distance** | **Ks** | **Ka** | **Ka/Ks** | **Mya** |
| --- | --- | --- | --- | --- | --- | --- | --- | --- | --- | --- |
| 1 | SiWD024 | SiWD025 | 0 | 85% | 0 | 6.212 kb | 0.35 | 0.05 | 0.14 | 26.9 |
| 2 | SiWD062 | SiWD063 | 3.00E-125 | 97% | 6 | 32.21 kb | 0.34 | 0.06 | 0.18 | 26.2 |
| 3 | SiWD083 | SiWD084 | 5.00E-61 | 97% | 4 | 25.68 kb | 0.32 | 0.03 | 0.09 | 24.6 |
| 4 | SiWD085 | SiWD086 | 1.00E-107 | 67% | 2 | 19.8 kb | 0.33 | 0.04 | 0.12 | 25.4 |
| 5 | SiWD121 | SiWD122 | 0 | 75% | 1 | 23.57 kb | 0.34 | 0.05 | 0.15 | 26.2 |
| 6 | SiWD170 | SiWD171 | 0 | 100% | 3 | 18.11 kb | 0.35 | 0.05 | 0.14 | 26.9 |
| **Mean** | | | | | | | **0.33** | **0.04** | **0.12** | **25.4** |
