# Supplementary material for: Genome-Wide Investigation and Expression Analyses of WD40 Protein Family in the Model Plant Foxtail Millet (Setaria italica L.)
Source: PLoS One. 2014 Jan 23;9(1):e86852. doi: 10.1371/journal.pone.0086852 (PMC3900672; doi:10.1371/journal.pone.0086852)
Supplement: Table S6 — The Ka/Ks ratios and estimated divergence time for segmentally duplicated SiWD40 proteins. (DOCX) [file pone.0086852.s009.docx]

**Table S6.** The Ka/Ks ratios and estimated divergence time for segmentally duplicated SiWD40 proteins.

| **Gene 1** | **locus** | **Chr.** | **Gene 2** | **locus** | **Chr.** | **e-value** | **%Homology** | **Ks** | **Ka** | **Ka/Ks** | **Mya** |
| --- | --- | --- | --- | --- | --- | --- | --- | --- | --- | --- | --- |
| SiWD004 | 3909585-3919710 | 1 | SiWD094 | 34858918-34868939 | 4 | 0 | 88% | 0.35 | 0.05 | 0.14 | 26.92 |
| SiWD015 | 26681841-26684653 | 1 | SiWD158 | 19749591-19752393 | 3 | 0 | 53% | 0.29 | 0.03 | 0.10 | 22.31 |
| SiWD020 | 32443084-32448521 | 1 | SiWD053 | 789987-794803 | 3 | 0 | 51% | 0.27 | 0.02 | 0.07 | 20.77 |
| SiWD020 | 32443084-32448521 | 1 | SiWD160 | 25638739-25643733 | 7 | 0 | 66% | 0.32 | 0.04 | 0.13 | 24.62 |
| SiWD021 | 33701665-33720640 | 1 | SiWD161 | 26759940-26781273 | 7 | 0 | 69% | 0.32 | 0.03 | 0.09 | 24.62 |
| SiWD023 | 35570463-35572819 | 1 | SiWD164 | 28937581-28940330 | 7 | 0 | 87% | 0.36 | 0.02 | 0.06 | 27.69 |
| SiWD024 | 36583968-36588374 | 1 | SiWD150 | 31892802-31896198 | 6 | 0 | 68% | 0.30 | 0.03 | 0.10 | 23.08 |
| SiWD026 | 36658790-36666593 | 1 | SiWD088 | 14008767-14017612 | 4 | 0 | 64% | 0.31 | 0.02 | 0.06 | 23.85 |
| SiWD027 | 37632822-37634265 | 1 | SiWD083 | 9562087-9563452 | 4 | 2E-158 | 62% | 0.29 | 0.04 | 0.14 | 22.31 |
| SiWD039 | 5387934-5392273 | 2 | SiWD0184 | 3013282-3016362 | 9 | 0 | 71% | 0.31 | 0.04 | 0.13 | 23.85 |
| SiWD053 | 789987-794803 | 3 | SiWD160 | 25638739-25643733 | 7 | 0 | 50% | 0.35 | 0.01 | 0.03 | 26.92 |
| SiWD058 | 6562814-6564965 | 3 | SiWD107 | 9930062-9935044 | 5 | 3E-157 | 57% | 0.33 | 0.04 | 0.12 | 25.38 |
| SiWD065 | 11267014-11269670 | 3 | SiWD127 | 33708532-33710607 | 5 | 0 | 86% | 0.36 | 0.01 | 0.03 | 27.69 |
| SiWD075 | 47708424-47710190 | 3 | SiWD199 | 11154999-11156471 | 9 | 7E-161 | 63% | 0.31 | 0.05 | 0.16 | 23.85 |
| SiWD167 | 33332115-33338289 | 7 | SiWD175 | 4731964-4739597 | 8 | 0 | 81% | 0.35 | 0.02 | 0.06 | 26.92 |
| SiWD170 | 34720822-34724697 | 7 | SiWD173 | 210527-214622 | 8 | 0 | 98% | 0.38 | 0.02 | 0.05 | 29.23 |
| **Mean** | | | | | | | **50%** | **0.27** | **0.01** | **0.03** | **20.77** |

Protein seq

>SiWD004

MGAKVHPGHPLPSLIACPFPFPPPLPHPSVTPTHSHFFASSRRHQPPSWFPPLTSSRHPLRRGAAWRAHGLAVVQTASGEWHARAHGRRRGVATARPRRGLWPQRCAGVQAWGLPRLRHGRGGVRDTGDPGPRRPAATRRWRRPTDYATLPRRKEQGEVSGSRQRDGGERRDDDFGRRSGRRLQLPFHERPPSSFSSFSSPLPPFSSIPLPPHGPLLPLSRVGGGGWAAPEEDGEGWAMWPAFANLPTSPRAAPAPPPGPAARGHRPRSAAPRAPTQLPRSANPRSTASHGCASARRPPRVAAPAVAVAGATMPLRLDIKRKLAQRSERVKSVDLHPTEPWILSSLYSGSVCIWNYQTQTMVKSFEVTDLPVRSAKFIARKQWVVAGADDMFIRVYNYNTMDKVKVFEAHTDYIRCVAVHPTQPFVLSSSDDMLIKLWDWDKGWVCTQIFEGHSHYVMQVTFNPKDTNTFASASLDRTIKIWSLGSPDPNFTLDGHSKGVNCVDYFTGGDRPYLITGSDDQTAKVWDYQTKSCVQTLEGHAHNVSAVCFHPELPIIITGSEDGTVRMWHSTTYRLENTLNYGLERVWALGYMKGSRRVVIGYDEGTIMIKIGREVPVASMDSSGKIIWAKHNEIQTVNIKSVGADNEIADGDRLPLAVKELGSCDLYPQTLRHNPNGRFVVVCGDGEYIIYTALAWRNRSFGSALEFVWSTDGEYAVRESTSRIKIYSKNFQEKKSIRPSFSAERIFGGVLLAMCTNDFICFYDWAECRLIRRIDVNVKNLYWADSGDLVTIASDTSFYILKYNRDVVSSHLDGGGSVGEEGVEDAFELLHEINERVRTGLWVGDCFIYNNSSWRLNYCVGGEVTTLFHLDRPMYLLGYLANQSRVYLIDKQFNVVGYTLLLSLIEYKTLVMRGDFDRANDVLSSIPKEQYDSVARFLESRGMLEEALEIATDTNYRFDLAVQLGQLEIAKAIATEVQSESKWKQLGELAMSTGKLDMAEECLLHAMDLSGLLLLYSSLGDADGITKLASVAKEQGKNNVSFLCFFMLGKLEECLQLLIESNRIPEAALMARSYLPSKVPEIVALWKKDLQKVNPKAAESLADPNEYPNLFDDWQIALNVEATVAPKRGIYPPAEEYMNYAERSNESLVEAFKNMHVDEEVLPDDNEDTVHEVVEDGGVEESQEDAVEVDAEDDVVEESQDDGVEVEAEGSTDGAVLVNGNSSEEQWGTKNEEPSA

>SiWD094

MSSLYSGSVCIWNYQTQTMVKSFEVSELPVRSAKFIPRKQWVVAGADDMFIRVYNYNTMDKVKMFEAHTDYIRCVAVHPTLPYVLSSSDDMLIKLWDWDKGWVCTQIFEGHSHYVMQVTFNPKDTNTFASASLDRTVKIWSLGSPDPNFTLDGHSKGVNCVDYFTGGDRPYLITGSDDQTAKVWDYQTKSCVQTLEGHAHNVSAVCFHPEVPIIMTGSEDGTVRLWHSTTYRLENTLNYGLERVWALGCMKGSRRVVIGYDEGTIMIKIGREEPVASMDNSGKIIWAKHNEIQTVNIKTVGADAEIADGERLPLAVKELGSCDLYPQSLRHNPNGRFVVVCGDGEYIIYTALAWRNRSFGSALEIVWSTEGEYAVRESPSKIKIYSKNFQERKSIRPAFSAERIYGGVLLAMCTNDFICFYDWAECRLIRRIDVNVKNVYWADSGDLVTIASDSSFYILKYNRDLVSSHIDGGASVDEEGVEDAFELLHEINERVRTGLWVGDCFIYNNSSWRLNYCVGGEVTTMFHLDRPMYLLGYLANQSRVYLIDKEFNVVGYTLLLSLIEYKTLVMRGDLERANSVLPSIPKEQHNSVAHFLESRGMLEEALDIATDPNYRFDLAVQLGSLEVAKEIAVEARSESKWKQLGELAMSTGKLEMAEDCLLQATDLSGLLLLYSSLGDAEGITKLASKAKELGKNNVAFLCLFMLGKLEDCLQLLVDSNRIPEAALMARSYLPSKVSDIVSIWKNDLQKVNSKAAESLADPAEYPNLFEDWQIALNVEATVAPKRGVYPPAEEYMTYADRSNESLVEAFKSMNVEEEIPSENGDPTHEVIEDDGVEESQEDAVEVEPDDSVDGGVLVNGNDGEEHWVLTPDQ

>SiWD015

MSSLDFDGQEDIFFDVSDDIRSSTCSTARCSTSDQLSASWRPEYELWASEPMSVNERRHRFLIGMGLAQPIPTGIAFPQWQGDTLDDCAFRDLEERISSICSSYQSSFSQFASAPDSINCERDLDTGNRVVVHESEHDTMTGIVEEVGTDIIMNINQSEGFLSFSQLVHEFLQKGGGRTHLRGSNLTVTDKQKDPKSFCGRFTRKKGEDRICMYDTHMKSLKTSTFSRTKVHQQNKKWIDFSAVYICQEIQAHGGSIRVMKFSPSGLYLASVGEDCIARIWMIQEVESSPDLYGREAPVEYMDRNKGLKMKVAKGQRRTLAIIPKKVFNIAETPLHEFHGHTSDILDMAWSKSDFLLTSSKDKTVRMWKAGCDGCLAVFKHRDYVTCVQFNPVDERYFVSGSIDGKVRVWDVSEKRVVDWADTRRIITAVSYQPDAKGLIVGTVPGRCRFYDQSGENMEVEKELKVTKKKSARRQITSLQFSRGDPARIMIASAGSKIRVSEGAGISRKFEGRRGSKVLVPPSLTSDGRYLVSAGADSNVYIWNFDKLRGKGTKGARTVRSCEHFFSDGVTSVATWPGLLHQEICESGGGGGDLQSSDKGPTLCRDRDCCSFGTWFFTDGVGGAAATWPEEKLLPSLKYLNCSGMDERRPKVPAAWNTVVVTGSRDGVIRCFHNYGLPVKL

>SiWD158

MPRSESDSDDIFFDAFEDVRSARELSSSEDCSTSDDVSAPRKFEYEIWANEPMSVQERRQRFLKGMGFDEFVSTRMDSFQCHGEITAVESSTDMEERTVSGHSSQESSVSDNESESDGACCIRDMDSGKRYIVNNGAHNSITDMLKEVGSDKMMSLLKFENLLGLSRSVQKLLRRGYGNSLARESKGASKKDDKSLWKKFMTKRSFSGICKSDVHVKNCTKRAPIRTKVQYRKKNFLEFSAINMDQEIRAHKGSIRVMKFSPSGWYLASGGEDCVVRIWQIIEVEAPPKLYKGEDPYEKVEKVQVFKTNTGKGQNHALAVIPKKAFRISETPMHEFHGHTSDILDLTWSKSDYLLTSSKDKTVRLWKPGCDGCLAVFKHKDYVTCVQFNPIDERYFISGSLDGKVRIWDVLDRRVTDWADTRNIITAVSYQPDGKGFVVGTTAGACRFYNQSGENIKLEKELFVQGKKKSSASRINSLKFCTSDSNRIIITSADSKIRVADGDSIKKFEGPWKSKALSSPSLTSDGRYLISAGKDSNVYIWNFPNSGDAKSVHSCELFFSKDVTNAVPWPGVHQDGHTKPSCLTEKSSSAPTLRRHGEPLSPGPWPFADGTKGSATWPEEKLPSAGKPESSPQLGDCLSAISAAWSTVIVTASRDGVIRSFPNYGLPVRL

>SiWD020

MSRSVARLPFVPPSRGNGNHPGCRRARARARAWWISSNAATCCYDFPIPPSPLPRPPPPPPPHLPPSLPPSGTVSSSLSLSHNSPNTPRAPRLALPTTAAAANAGGAMSVEEEVEEQEGEEELFYESLDRILSSSGSSTSASDDDGAARPRRRRGGCDASAAALDLWTSQPAPVQERRRRLLQLLGLAGDPSLARFGAGRSASDAAVDSLPASPVSRSRSGGVALGSAAKPPLGGARLRPSLSDASDAALEAVEEDPRCLIRNLDDGSEYVVREELCLREVGTGRQLTVEELGRSPIVQELMRRQAFSTPNSNCTSNSQSGASTPIERSSSSSSNGGARSKRRSSWLRSIRCVAGSMATHSRDRRSSDEKDTSSEKGGHHSSSATDDSQDRVPRHGPSRVKVRQYGKTYKELSSLFMTQDIQAHNGSIWSIKFSPDGRYLASAGEDCVIHVWEVLEFERVGKERAVEENGVCNESSELMVSSAAPSGSHWEKKLRSKVLHSGGSVSSDRLMVPEYVFALSEKPVITFAGHSEDVLDLSWSKSQYLLSSSMDKTVRLWHMSSTYCLKTFSHSDYVTCIQFNPVDDRYFISGSLDEKVRIWSIPKREIVDWVDLHEMVTAACYTPDGKGALIGSHKGSCHLYDTSDDMLCYKKRIDLQIKKRKSSQKKITGFQFVPGSPSKVVITSADSRIRVLDGFELVNRFKGFRNTSSQISACSAGNGRHIISASEDSHVYMWRNNDDFEPSRKKGTVSVTNTHEHFHCKSVTVAVTWPFTSTTMTSFMNPRKQEELDCGLENDHVLQTKPAKAQEMPDVKYQSTTITSNNSNHTGDWTSVTWPEELTTPAKQSHRPTTCLADEGDQVPNRSAWGLVIVTAGRGGQIRTFQNFGFPVRV

>SiWD053

MPEAAARPPGPDPAGEEAEQEEEFYESLDRILSSSCSSTSASDDDADHRRRRRSHRHLQQPPPTHASAYDVWISEPTSVEERRRLLLQRLGLSSEPEPPPQQPPSPRRSPRSPSPPASPPASPPLAAEEPRSGGLGKPPLARNPSSSGGEQCRIRNLDDGTEFEVGEVHEEVVREVGTGRQLTFEEFELCVGRSPIVHELMKRTTTAASSSASDHAAPASKPRRKPGGGWLRGIRQLAGSVAYGRRSTDEGEKEKDKKEREARRLSSATDDSLDGTGSRNAAGRVRVRQYGKACKELTGMFMTQELAAHSGSVWCINFSLDGRYLATAGEDRVIHVWEVSEGERKGELLGEASVTKENGGSCSPFLAVVGNDSPEIAALSLTCADGGYVDKKRRPRKQSNRKSVGSDHLVVPECVFGFRDKPVCSLLGHAADVLDLSWSKSQYLISSSMDKTVKLWDITTSTCLKTFSHTDYVTCIQFNPVDDNFFISGSLDEKVRIWNVRDRKIEDWNDLHEMVTAACYSPDGQVALVGSHKGSCHIFDTSEKKLQYKSQIDLRIRKKKSGQKKITGFQFAPGSSSEVLITSADSRIRVVNGDELVHKFKGFRNTSSQISASVAPNGKYVVCASEDSHVYVWRHDNSSHPSRSRSTVDVTNSYEHFHCHGVTVAITWPGSEARGSFGSRSSRHSDSDGAVNSGRDVPAENTEHNSDAADNRYNESPVCEGVASRSTSKPPGDGASTSWPDEKLPSAKSSPGHCSSDLCIGAMDVQRRSAWGLVIVTAGRGGEIRVFQNFGFPVQV

>SiWD160

MSAGGGEEEEEEEEVFYESRERVLSSSGSSTSASDDDDHGLPRRRRDGTASAAAAAAAALDVWMSEPAPVQERRRRLLQMMGLAGDPALARLEMGRSASYDGPVRPAAVSPISRSRSDGAAPVKPPLGGRSRQASSGSSEATPEGEEADPRCLIRNLDDGSEFVVKEEFELREVGTGRQLTMEEFVDLCVGRSPIVQELMRRENVANAGSNNDSSTPIQRSNSDSSNGATRHRRRSSWLRSIRNIAGSMVVTSRDRRSSDEKDTCSEKGGRRSSSATDDSQDSARAVHHGPVRVKVRQYGKSYKELSGLFMNQEIQAHNGSIWSIRFSPDGRYLASAGEDCVIHVWEVSEFERKREENGVCNPFVAMVCNGSPEPTLALASLDGSNSEKKRRARFLESRRSVSSDQLMVPEHVFALSEKPIRTFVGHSEDVLDLCWSKSQYLLSSSMDKTVKLWHISSTSCLKTFSHSDYVTCIQFNPVDDRYFISGSLDEKVRIWSTQNREIVDWRDLHEMVTAACYTPDGQVLIIYTSFLDLNIFVLRSNMFPSYLKSALIGSHKGSCHIYDTSDNRLLQKKQIDLQNKKKKSSQKKITGFQFLPGNTSRVLITSADSRIRVADGLNLVHKYKGFRNTSSQIAACLAANGRYVISASEDSHVYIWRNDDNLEQGRSKGNVTVTNSYEYFHCQDVTAAVALPSAGSAMVSRTNSRKHDEQDCVSEHPLLHAVPELQDSCDFQGQSGNILSTSSNHSGDRATWPEELMTATKQSPRSSASLPSGAGQAPSRSAWGMVIVTAGRGGQIRTFQNFGFPARV

>SiWD021

RRHFFAFPARPLLLSLPPPLGARPRPCTAPPPLISREHFPKTPTSLLPFGDEAYAVNDEEVSDTEMSACSPSAPSEPSPPLRRRLAPVVASDVPEEVVRAVDAVIMGGGVEHLREMVSEENGEVSHFIVDVLMVTMGGVDGLDEGAGDGLGAATGLPPSIMSSSRAAAIAAELVPYLPCGVEPSPRTRMARGLLATLSACTRNRTMCSASGLLAVLLDVAEKLFVGMGQGSKWDGTPIVQCIQVLGGHSVSVRDLHSWLLLIKKALVTRWATPLTLALENAVASNEAKGPAVTFEFDGESSGLLGPGDNRWPFSNGFGFATWIYVESFSASLDTDTASAAVAAAAASTSGKSSPSAAAAAACTLAGEGTKHMPRLFSFLTSDSHGIETYFNGQFLVVESGAGKGKASLHFTYEFKPRCWYFVGLEHTSKQALLGKAESELRLYVDGDLHESCPFEPPRIVKPLAFCCIGTNPSPTIAGLQRRRRQCPLFAEMGPIYIFTDPIGPERMSRLASRGGDALPSFGNGAGFPWKSTSNHIREAAEDSYALDIEIGGSLHLLYHPSLLNGRFCSDASPSGSTGTHRRPAEVLGMVHVSHRVRPAESLWALAYGGPMALLPLIVSNVEMDNLEPILGDLSLSLATASLSVPIFRIISLAVQHPGNKDELCRIHGPELLSQVLHYLLDTLSKIESGKKEILSAEELVTAIVSLCQSQRNDHGQKVQLFSTLLLDLKMWSSCNYFLQKKLLSSLADMVFAESTCMRDANALQMLLDGCRRCYWVIHEADSIDTFTLTGHERPLGNVNALVDELLVVIELLIGAAPSTFASDDVRCLVGFVVDCPQPNQVARVLLLIYRLIAHPNTSRANLFAQSFISRGVVEALLVLLQREAKSGDSNIFHSSNVPQNAASWNGSSNLTNKDLELKTANGEESCKDHQIQSVQHQEPTSHETGSGHESTSKWCLLKGQFLNNLGGIDVPNISDNVQNSVYNIDGGDGVLVGIVHVLGALVASGHLTFTSSTVRPKLPSGFLTTCKGEGNTMFEDRVSLLLFALQKAFQAAPKRLMTRNVYRALISSVIDVSSPKDNLKNLHDSGCHFKHIPLLMVLLCSLPYASRAFQAHAIQDLLYLVCSHPKNRSTMTSISEWPEWILEILISNHEDVEAAIHCAEWLSMVGGSSTGDQRIRREESLPIFKRRLLGSLLDFSAQELQVQQTGGIAATAAGVAVEDLVPKETKVQAEKAANLSVALAENAIVLMMLVEDHLRSRSQHFFMSCLVDSAASPASVASLAASRSNSLSRSGSEHLEAGGSRQSLSSDAGGLPVDVLASTADTNGQLSAEVMERVTAAAAAEPYGSVRHAFVSYGSCISDLSEGWKYRTRLWYGVCIPPKSNVFGGGGSGFVSWKSVLEKDSNGNWIELPLVKKSVAMLQALLLDSGLGGCLATGVGSGPGMGVMGALNQLLDSDQPFFCMLRLILVSMREDDSGEDDIFTRNISMKNEISEGLGCQTGSMLPLDGHSSASIKKCPAALLWRVLGPILNMPVSETKRQRVLVASSILYSELWHAVSSDRKPLRKKYVGLIMPPFVAFLKRYRSILAGIHELTSPDAQNPLAVDDWASAADTSPVEVGVSMISPGWAAAFASPPVAMALAMIAAGASGTETIAPPTNKLRRRDTSLLERRSAKLHTFSSFQKPLDTTPSLPTSAPKDKAAAKAAALAAARDLERSAKIGSRRGLSAVAMATSVQRRSAGDIERAQRWNTSEAMGAAWMECLQSADSKSVSGRDFSALSYKYVALLVSSFALARNLQRVEMERRTQVEILNRSCMSIGLRAWRHLLHCLIETSRLYGPFGELLCTPDSIFWKLDLTESSLRMRRFMKRNYNWLNHLGATANYGEQKFLCDGADSNACHSEDGDSLPTNVLSTSSLITVDGGHEDIRQGETENICSSVDDQLTNSSPLDQSLTGSVDSRSSDFSGVRNLVRSTVVAPGYRPSNERIIIELPSMMIRPLKVVRGTFQVTSKRINFIVDEHTSDSYMDDITSTSGQYDQQDRDRSWLISSLHQIYSRRYLLRQSALELFMVDRSNFLFDFEDVGARTHAYRAIVHAKPPYLNDIFLATQRPEQILKQTQLMERWAKWEISNFEYLMELNTLAGRSYNDITQAFFYYMILSPGSNMFATVPYVLYLFLQYPVFPWVIADYKSKTLDLESPSTYRDLSKPIGALNPARLKKFQDHYSSFKDPIIPKFHYSSHYSSPGTVLYYLSRIEPFSTLSAQLQRAKFDHDDCMFSDVNKTWNSVLEGLDDVKELVPELFYLPEVFTNLNSSGRLGSVALPPWAENPVDFIHIHRKALESDHVSTHLHEWIDLIFGYKQRGKDAVVANNVFPHVTYDGMVDIDKITDPMQRRATQNQISNFGQTPSQLLTVPHIRRRTLTEILQVQTIFRNPNEVRSYALPSPDHCNVPASAMLISKDCIVVIDSNVPTVHVALHHWQPNTPDGLGAPFLFHHGKNAINSSGGAIFRIFKGSSGSTEDYQFPRAVAFAASAVQNSSAVVVTCDKEVITGRHADNSVKMISPDGARTVETAFGHLAPVTCLALSADSNYLVTGSRDTTVILWRIRQVGSSQEKNAPEPPPSTPTTPTSPQATGSSSNSSSSKNLETYRRRRIEGPMHILRGHLGEVTCCSVSSDLGLVSSSSVSGVLLHSLRTGRLIKKLDVPEAHSICLSSQGIVLIWNESEKRLSTFTVNGIPIATSVLSPFSGRVSCIETSADGQFAVMGTCSASNCNHKGSNATEDDYELDKPCGDEDVQESNETRLSVDAPSICLLDLYKLEVIHTLKLGEGMDVTALALNKENTTLLVSTADKQLIVFTGPAANNPMSLKVADRSVLHESDGLLKP

>SiWD161

MAEDAREVSDSSSPPAPAPPTPRPEEGSEEEQFEAVALGDEAGVEEEEPASVPGMGASTPATPVTPYEPSPRPRRPPRPPGAPADAPPEVVRAVEAAIAGGPDLLREVASQEQGELAHSVVDVLLGTMGGADEAGDSTGTGAPPSVMSNARAAVVAAELLPCLPCDDEPSPRTRMVAGLHAALRACTRNRAMCSCAGLLAALLDSAEKLFVEMDPGSSWDGAALLQCIEVLGGHSLSVKDLHSWLGLVRKALGTSWATPMTLALEKAMGSEEARGPAVTFEFDGESSGLLGPGDSRWPFLNGYGFATWIYIESFSDTLSTATAAAAIAAAAAATSGKSSAMSAAAAASALAGEGTTHMPRLFSFLSSDNQGVEAYFHGQFLVVESVGGRGKKASLHFTYAFKPQCWYFVGLEHTNKHGLLGKGESELRLYVDGSLYESRPFEFPRISKPLAFCCIGTNPPPTIAGLQRRRRQCPLFAEMGPIYIFKEPIGSDRMRRLAFRGGDTLPSFGNGAGLPWKATNDHVKSMAEESFALNNELAGGLHLLYHPSLLTGRFCPDASPSGSSGTQRRPAEVLGLVHVSSRVRPAESLWALAYGGPMALLPLTISNVQMDSLEPIPGDLSLSLATVSLSAPVFRIISLAIQHPGNNEELCWTFAPELLSRVLHYLLQALSKVESGEEALTDEELVAAVVSLCQSQRNNHELKVQLFSSLLLDLKLWSSCNYGLQKKLLSSLADMVFTESACMRDAKAMQMLLDGCRRCYWAIQEPDSIDNFAFTGTKRSLGEVNALVDELLVVVELLLGAASSTAASDDVRCLIGFIVDCPQPNQVARVLHLVYRLIVQPNISRANMFSQSFISSGGVEALLVLLQREAKAGNKNILDDSGANLSENDVHRDRSSSRKVESADTRCQVDETQSTEHHETIFHEEADEHEASNANDMLESNIGSRVPGSENGLLKNLGGISFSITSDNVRNNVYNVDKGDGIVVGIIHILGALVASGHLKFDSDAATPNIPGGSQNALNEEGNPVSEDRVSLLLFAFQKVFQAAPRRLMTANVYMALISAAINVSSADESLNLYDSGHRFEHIQFLLVLLRSLPYASRAFQARAIQMGTKKNADGVSIGEIEDLIHNFLIIMLEHSMRQKDGWKDVEATIHCAEWLSMVGGSSTGDQRIRREESLPIFKRRLLGDLLDFSARELQVQTEVIAAAAAGVAAEGLSPEEAKAQAENAAHLSVALAENAIVILMLVEDHLRSQGQHFCTSLAGDSIVSSTSVASLAASRSNSLGTAGKEPTAAGASRRSSLSSDAGGLPLDLLTSMADANGQISAAVMERLTAATAAEPYESVKHAFVSYGSCIADLGESWKYRSRLWYGVGIPPKSDIFGGGGSGWESWKSVLEKDSNGIWIEFPLVKKSVAVLQALLLDESGLGGGLGIGGGSGPGMGVMTALYQLLDSDQPFLCMLRMVLVSMREDDKGEGDAFMKDNNIKDVVSEGMGHQAGSMMPFDGNSYSSPEKPRSALLWSVLGPILNMPITESKRQRVLVASSILYSEVWHAIGRDRKPLRKQYIELILPPFVAILRRWRPLLAGIHELTSSDGQNPLIADDRALAADALPIEAALLMVSPGWAAAFASPPVAMALAMMAAGASGTETRTPPRNTVNRRDTSLPERKAASKLQTFSSFQKPIETAANKPGSTPKDKAAAKAAALAAARDLERTAKIGSRRGLSAVAMATSGQRRSSGDIERAKRWNTSEAMSAAWMECLQSADSKSVAGRDFSALSYKYVAVLVSCLALARNLQRVEMERQTLVDVLNRHRASTGLRAWRHLLHCLTEMGRLYGPFGEPLCTPVRVFWKLDFTESSSRMRRFMKRNYKGSDHLGAAADYEDRKLLSTAAQSNECNSEDANSSLANALPSSASVIMADAMSMDERNAENEQLETDTTHSSVDDDQLQHSSAADKQSVKGSVGSRSSDICADRNLVRSTVLAPSYVPSEADERIIVELPSLMVRPLKVVRGTFQVTSKRINFIIDECSSDNNIDDAASTSGQCDQQDKDRSWLISSLHQIYSRRYLLRRSALELFMVDRSNFFFDFGDIDARKNAYRAIVHTKPPNLNDIFLATQRAEQILKRTQLMERWANWEISNFEYLMELNTLAGRSYNDITQYPVFPWIVADYKSGVLNLDDPSTYRDLSKPIGALNPERLKKFQERYSTFEDPIIPKFHYGSHYSSAGTVLYYLFRVEPFTTLSIQLQGGKFDHADRMFSDLSGTWDSVLEDMSDVKELVPEMFYLPEVFTNVNGIDFGTTQLGGKLDSVELPPWAENHVDFVHKHRKALESEHVSAHLHEWIDLIFGYKQRGKEAVMANNVFFYITYEGTVDIDKITDPVERRATQDQIAYFGQTPSQLLTVPHMKRKPLAEVLQLQTIFRNPNELKSYVLPHPDRCNVPASAMLVSNDSIVVVDVNAPAARVALHHWQPNTPDGQGTPFLFHHGRNAANSTGGALMRIFKVSAGSAEDYEFPRAIAFAASAIRSSAVVAVTCDKEIITGGHIDGSLKLVSPDGAKTIETASGHLAPVTCLALSPDSNYLVTGSRDTTVILWRVHRTGSSHKKNAPEPPPTTPTTPRSPLSSNTSSVSNLSETKRRRIEGPMHIMRGHLGEVTCCSVSPDLGLVASSSNTSGVLLHSLRTGRLIRRLDVCEAHAICLSSQGIILVWNESKKTLSTFTVNGLPIATSILTPFSGQVSCIEISTDGHFALIGTSSFNNYKCDEITESGDHELGPSGKDDVSKDSEQSETEQSVHVPSACFVDLHRLKVFHTLKLAKGQDITAIALNKENTNLLVSTADKQLIVFTDPALSLKVVDQMLRLGWEGDGLLQ

>SiWD023

MDAGSLSISSEKSRAAAPRPPLQEAGSRPYMPSLSTGSRNPSAKCYGDRFIPDRSAMDMDMAHYLLTEPKKDKENAAGVVASPSKEAYRRLLAEKLLNNRTRILAFRNKPPEPENVSFADAASSNLQAKPAKQRRHIPQSAERTLDAPELVDDYYLNLLDWGSNNVLSIALGDTVYLWDASSGSTSELVTVDEDSGPITSVSWAPDGRHIAVGLNSSDVQLWDTSSNRLLRTLRGVHEGRVGSLAWNNSILTTGSMDGNIVNNDVRIRNHVVQTYEGHSQEVCGLKWSGSGQQLASGGNDNLLHIWDVSMASSMPSAGRNQWLHRLEDHMAAVKALAWCPFQSNLLATGGGGSDRCIKFWNTHTGACLNSVDTGSQVCALLWNKNERELLSSHGFTQNQLTLWKYPSMVKTAELTGHTSRVLFMAQSPDGCTVASAAADETLRFWNVFGAPEAPKPAAKASYTGMFNSFNHIR

>SiWD164

MDAGSNSISSQKCGSRRAAPPRPALQEAGSRPYMPPLSTGPRNPSAKCYGDRFIPERSAMDMDFAHYLLTEPRKGKENPAAAVSPAKEAYRKLLAEKLLNNRTRILAFRNKPPEPQNVLTDLRSDVVQAKPAKQRRHIPQSSERTLDAPELADDYYLNLLDWGSSNVLSIALGSTVYLWDASSGSTSELVTIDEDFGPVTSVSWAPDGRHIAVGLNSSDVQLFDTTSNRLLRTLRGVHELRVGSMAWNDSILTTGGMDGKIVNNDVRIRNSVVQTYHGHEQEVCGLKWSGSGQQLASGGNDNLLHIWDVSMSSSVQSAGRTQWLHRLQDHLAAVKALAWCPFQSNLLASGGGGGDRCIKFWNTHTGACLNSVDTGSQVCALLWNKNERELLSSHGFTQNQLTLWKYPSMVKMAELNGHTSRVLFMAQSPDGCTVASAAADETLRFWNVFGTPEAAPKAAAKASHTGIFNSFNHIR

>SiWD024

MQGILWERLPVTREKYRQTRLEQYKNYENVPNSGEEAIKDCNPTEKGGMYYEFRQNTRSVKSTILHFQLRNLVWATSKHDVYFTSSYSIRHWSALSGMNTELMNVEGHVAPREKCSGSLSEGFSQTQVSTLAVKDNLLIAGGFQGELICKHLDREGISFCGRTTYDDNAITNAVEIFNTSSGAVHFIASNNDSGVRDYDMEGFRLCKYFQFEWPVNHTSLSPDRKVVVIVGDDPDGLLIDANSGKTLHSIKGHRDFSFASAWSPDGRTFATGNQDKTCRIWDTRNLSKAVHVLRGNLGAIRSIRFTSDGQFMSMAEPADFVHVYDVKSDYNRRQELDFFGEISGTSFSPDTDMLFVGVWDRTYGSLLQFGRLHNYSYLDSLF

>SiWD150

MSLYDGELTGVYPEENEDHHAVQMAESDYEDDDSDQPTCKETEDTTAMDVKKGKDIQGISWETMNTTRDRHRQARLQQYANFENIPNSGRTAEKDCTPAKKGQLYYEFQHNTRSVKSTILHFQLRNLVWATTRHDVYLMSYYSVLHWSALTREKQEIIDLQGHVAPCEKHHGNFSEGFSRTQVSALAVKNNLLVTGGFHGEIICKFLDRQGISYCCKSTHDDNGITNSLEIYEKPSGSLHFLASNNDCGVRDFDMEKFQICNNFRFAWPVNHTSLSPDGKLAVMVGDSPDGLLVDANSGKAVHDLRGHLDFSFASSWNPDGRTFATGNQDKTCRVWDIRNLSKSVAVLGGNIGAIRSIRYTSDGKFMAMAEPADFIHIFDVESGYSRKQEVDFFGEIAGISFSPDTESLFVGVHDRANSSLLQFNRRRFYSYLDSVL

>SiWD026

MASPTGNPNPNPNPPFELGKLFRPPNPMPTATATAATIFPGAAGGPAGPPPPSGPYSYPPVTPPFHRGPYLHYPQDPHAMPRPVVSFPMPNPNLNPNPNANPNAAVPGPNPGVRLMQLLGNSGPTQLETAVSMPPPTSEFAQPLPAMPSAPPARMLSSTSSKVPRGRLLGGGERAVHDIDSRLPGEAQPPQLEVTPITKYTSDPGLVLGRQIAVNRTYIVYGLKLGNIRVLNINTALRSLLRGHTQRVTDMAFFAEDVHRLASASVDGRIYVWRIDEGPDDENKPQITGKIEIAIQIVGEVEAYHPRICWHSHKQEILFVGIGNCVLRIDTTRVGRGRDFAVEEPVKCHLEKLIDGVRLVGKHDGDVTDLSISQWMSTRLASGSKDGMVKIWDDRKPNPLSILKPHDGQPVYSVAFLTAPERPNHINLITAGPLNREIKIWASTNEDGWLLPSDSESWNCTQTLELVSSLEPRVEEAFFNQVAVLPQASLILLANAKKNAIYAVHVDYGPDPASTRLDYIADFTVAMPILSLTGTHESQPDGEQVVQVYCVQTMAIQQYGLELSLCSPPTADTTGFGRDPAISRVYEAPPEVAGTESSTTSFTDSYSVSASSKPPTADQSAEFDPKPSAPPLAYSEGDGSVHLPSAPPASKMELPGSGPAPGTRDIDQSAFDYTANRNMERDALKRQDTPMPIRKDILGKDELRDGHSDVAMLPNPRLMFQVGGNATHLVTPSEIISGTLSSAENNDVSKSDGGKIQDVSSRSSRIAELEPKHIDESKPDQNSGLEAVKEAQIVCEHMEKTRSLEQTVEMISERSVTTDKYSVEESQAPSDKPTLDHTGVADENVRKNSLEMPEKSDYSASREQSSSYTKEEKVLHPQTSGQPSPSVSAFNSTESHEPLSSAYPPISSFPEVAATQGMLQQLIGMQKDMEKKLDTMIPVSVAKESKKLETSLGRTMEKSIKAHFDAFWVRLQEENTKREKADRERMQQLVTLITSSINKDVPSNLEKSLKKEISSLGPVVARAITPIIEKCIASAVSDSVQKGVGDKVCNQLDKSISGKLEATLARQIQMQFHTSVKQALQDALRTSFESLLVPAFEQSCKTMFEQVDGTFQKGMSEHTVAIQQQLEAAHTPLALTLKETINSASSITQSFSSELLDGQRKLLALVASGNAKAHTPNALQPINGPMGGPQEVKVEAPLDPMKELGRLVSERKFDEAFTMALQRSDVSIVSWLCSQVDLRALLAMVPVPLNQGVLLALLQQLAVDINNETSRKVQWMTDVAMAINPADPMIAVHVRPIFDQVYSQLAHQRSLPTMSSSDGTSIRMLMHVINSVLLSYK

>SiWD088

MASPSGNPNPTPSAPFELSKLFRPPPNPNHPTTAPSPTGVFPGAPGPAAGPPLTGPYSYPPATPPFHRGPYLQYPNDPHGFHHPAAAAFANANPTANPIPNPGPGPNPGARLMQLLGNTTPTHLESAASMPPSSEFSTAPAVALPASSSAPPARMLSSKMPRGRLLGPGDRAVHDVDSRLPGEAEPPQLEVTPITKYTSDPGLVLGRQIAVNRTYIVYGLKLGNIRVLNINTALRSLLRGHTQVTDMAFFAEDVHRLASASVDGRVYVWKIDEGPDEENKSQITGKIEIAIQIVGDAETYHPRICWHSHKQEILYVAIGNCILRIDTTKVGRGRDFHTEEPLRCPLDKLIDGVNIVGKHGGDITDLSISQWMTTRLASASKDGTVKVWDDRRVAPLSVLKPHDGQAVYSVSFLTAPERPNHINLVTAGPLNREVKIWASTNEDGWLSPSDPETWKCTQTLELVSSLENRSEEAFFNQVAVLPQASLILLANAKKNAIYAVHLEYGQDPASTRLDYIADFTVAMPILSLTGTHENQPDGEQVVQVYCVQTMAIQQYGLELSLCLPPPADNIGSGRDPAISHLNERLPEMAALDSTATTPVDSSTAVSTKPSSDSQGTAPKSKMNQAGSPVVLSRDPSGSDRDVDQSSFGRKDSIGKEEPRGGHSDGMVISDPRPVLQVGGHATHLITPSEIISGGLTSAETVASGSSQNVEAEAKHVDERKSNQTVGFEAGKENQILPEKKGRPIKPSEQTVDTLSERTIVTAKYSVEDSQPMADRSVPTLLKQSSGAEDEDAVKRATGASDGTGTDGPCTSRDLPLTSAAKEGKVMHPQPQVAGQLSPSATTFNSTDSSHEPRSNENPPIDSSLQAAAIQGTLQQLIATYGNLQKQLSSIVSAPIAKEGKRIEASLSRNMEKSIKANIDAMWARFQEENVRHEKYERERMQQMATLIATSVNKDIPVMLEKSLKKEISSLGPAVARTTAPIIEKSLSSAVSDSLQKVLGDKVANQLDKSISTKLEASVARQIQTQFQTSTKQILQDAFRSSFETSVIPAFEQSCKTIFEQVDGSFQKGMSEHGAAIQQQVLTAHTPLAQTLKEAITSASSMNQGLNSELLDGQRKLLSLFASGSPTSQKTGALQPSNGPVANLPEVDAPLDPMKELGRLIAERKIDEAFTMALQRSDVSIVSWLCSQVDLQALCGAVPIPLNQGVLLALFQQLACDIANDTSRKLQWMTNVAVAIQPTDPIIAMHVRPIFDQVYGVLAHQRSLPTTNASDATNIRLIMHVITSVLISHK

>SiWD027

MNSAGEQEPSQIQHEPAGAMLSQAVSLSSQPSLPSLPSLGPRDQSVIPSLHQCLATLRAHSSYVSALAVDGDSLYSASSDGRIRVWPLDGGASGGQEQQQDDSGGSATVVAACDSSVKCLLAMGDGLVLSSHQDGKIRAWRAGSRKDGSRRLAPRAVLPTCVDRLRTFLLPWSYVQVRRHRWRTWVHHVDAATALAVSPDGALLYSASWDRSLKAWRLPGFQCAESVAAAHDDAINALAVSPDGHVYTGSADKKIKAWRRQPERRSKHVLVQTMERHRSAVNALALGVDGKVLYSGACDRSVVVWERAGDGRMEATGTLRGHKKAILCLAAAGDVVCSGSADRTVRVWRRGAENTGYTCLAILEGHGAPVKSLTLVYGRDRGSFGGGGGGSALVCSGALDGEVKIWSVLVPCLLER

>SiWD083

MSSIRQEETQAQDVSETEHTISFLSQSNRPSLQNSANPSLSSCLYQCIATLKGNSFYVSSLAIDGDSLYIASSNGHIRLWPLDMAMDVRQAEHGQSSSTVAVTNSSIKCAIATSNGLVSSHQDGKIRVWHHPARRNGSSDHHLALRAVLPTAADHLRTFLFPSNYVEVRRHRRRTWVRHADAVTALALSPDGAEMYSVSWDRSLKAWRLPGLRCAESIAAAHDDAINAVAVSADGSVYTGSADRTVKAWRRRPGRQGKLALVGTMERHKAAVNALALGVGGRVLYSGACDRSVVVWECAGGGAMCATATLRGHMKAVLCVAAAGDVVCSGSADRTVRVWRRGAAGAGYTCLAVLDGHAGAVKSLTLVKKSGGDHDGSCDGCCSCSAAHVCSGSLDSDVKIWRVNVSCL

>SiWD039

MGSGGGEEASAAGGGKVACAAWIRRRDEKAAAARVFAAHARAGAAGSLPAVEVLGFDSKECSLSPEPLARAVLGEGGAGDAPRGIAVHPAGDELVCATAKGCRLFKLIFEEFAVRIIPRDAPPLESVGPQKCLAFSTDGAKFAIGGEDGHLRIFHWPTMNVLLDEPKAHKSFRDMDISLDSEFLVSTSIDGSARIWKIDEGTPLVNLTRSSDEKIECCRFSRDGMKPFLFCTVAKGSKVVTVVWNISDWKRIGYKRLLGKPISTLSVSLDGKYLALGSHDGDFCAVDVKKMEVSHWSKKAHLGSPVTSIEFCPTERVVISTSNQWGAELTKLNVPADWKEWQVWLVLLALFLASAVLFYMFYERSDSFWKFPMGRHQPAKPWSVLKESPPVPEDQTPW

>SiWD184

MAGNGGEGAALGKVTCAAWIRRRDDDGPTGVSRLLVAFGRGATASSPPLLELLEFDVRASALASEPLARVVLGEDADDTARAIAVHPGGRELVCATAKGCRVFKLVYRDFGVHLISREASPLQSVGPQKCLTFSTDGAKFAIGGEDGHLRIFHWQSLNVILDEPKAHKSFRDMDISLDSEFLVSTSIDGSARIWKVDEGSPLVNLTRSPDEKIECCRFSRDGTKPFLFCTLVKGHNVWTMAVDISNWKRIGYKKLSAKPISTLSVSLDGKYLALGTCDGNFCVVEVQKMEVAHWSKKVHLGSPISSIEFCPTERVVISTSHQWGAEITKLDVPPEWKVWQIWLVLLTLFVSSAVLFYLFFKHARIHL

>SiWD058

MAHAAVAADDSTAVDTDDDNSFRLLSVSWNQDSSCFAAATTADFRVFSCAPFHEKLRRVHPEGGGYAVVEMLFRSNIFALVAAGEAGRHRVELWDDSQGQSVYDIPGIRSAVRAVRVSRAYLAVVLDRTVRVYRLTDPARPRWKIPTALNPRGLCCLSSHAGAPPVLACPGTARGQVRVEHLGTKEQAATSVAAHSSDIACMAMTPDGAVLATASVKGTLVRVFSTMDGTCLQEVRRGRDQADIYSIALSPNVQWLAVCSDKGTLHVFSLRVRDVKKDAGGKQSAEASSVVQTNTASNARSSLSFMKGILPDYFSSEWSFAQFRLPETTRYVAAFGEQNTVMIIGMDGSFYRCSFDPVNGKEMVRKEYFRFLKDKDSPPIRT

>SiWD107

MSSQVSTSRGLHPPGKLGAFESSHVWPLSAPTSQPGAGLGDDQDVRLLSVAWNQDCGCFAAGTSNGFRIFNCEPFKETFRRDLKSGGFGIVEMLFRCNILALVGGGSNVQYPPNKVMIWDDHQSRCIGEFAFRSDVRAVKLAKDYIVIVLERKIYVYNFTDLKLLYQIDTLSNPKGLCCLSHHSNTSVLACPGVHQGHVRVEHFGLKMTKTIPAHDSNISCMALTMDGLLLATASTKGTLIRIFNTMDGTRLQEVRRGLDKAEIYSIALSPNVQWLAVSSDKGTVHIFSLRVRVAGEDSSNEQHTLEGPRMDHQNSSSSIDPLIQTNTGSNASSSLSFMRGILPKYFSSEWSFAQFHLPEVTRYIVAFGAQNTVMMVGLDGSFYRCSFDQVNGGQMLQKEYFRFLKSDSPPFRTSAS

>SiWD065

MAKRKRKETKDIHPDDPTSYPTASTSCFRSFPLLSLSRAAAAAAATTPTLAAPTSSATMASGQESLSLVGTMRGHNGEVTAIATPIDNSPFIVSSSRDKSVLVWDLTNPVHSTPDSGAAADYGVPFRRLTGHSHFVQDVVLSSDGQFALSGSWDGELRLWDLSTGLTTRRFVGHEKDVISVAFSVDNRQIVSASRDKTIKLWNTLGECKYTIGGDLGGGEGHNGWVSCVRFSPNTFAPTIVSGSWDRTVKVWNLTNCKLRCTLDGHGGYVNAVAVSPDGSLCASGGKDGYTLLWDLTEGKRLYSLDAGSIIHSLCFSPNRYWLCAATQDSVKIWDLESKHVVQDLKPDIQISKNQILYCTSLSWSADGSTLYTGYTDGSIRVWKISGFGYAG

>SiWD127

MAGAQETLVLAGVMRGHNDVVTAIAAPIDNSPFIVSSSRDKSLLVWDLTNPVHVAGDGTTTADYGVPFRRLTGHSHFVQDVVLSSDGQFALSGSWDGELRLWDLSTGLTTRRFVGHEKDVLSVAFSIDNRQIVSASRDKTIKLWNTLGECKYTIGGDHGASEGHSGWVSCVRFSPNTIQPTIVSGSWDRSVKVWNLTNCKLRSNLQGHGGYVNAVAVSPDGSLCASGGKDNVTLLWDLTEGKRLYALDAGSIIHSLCFSPNRYWLCAATQDSIKIWDLESKHIVQDLRPEVSAGKNQILYCTSLSWSADGSTLYAGYTDGTIRIFKISGFSYSV

>SiWD075

MGNHKKLLQFLRPDPAVAAAPKPPPPSSSDDDDDDYACSTPTTPTTGATTPTTATTNSAPSSPFAMSPWTQLPGLGFGAGDGDHHGAAGDTTRTGLLGSLVKADGHVYSLAAAGDLLYTGTDSRNVRVWRDRRELGGFRSSSGLVKAIVVAADGRIYTGHQDGKVRVWRRASSPPEDPAAAAHHRRVGSLPRLRDVLTSSLLPSQYVETRRRRAALWMRHFDAVSSLCIDAAAGLIYSGSWDRTFKVWRVSDSKCLESVIAHSDAVNAVAAAGFDALVFTGSADGTVKVWRRGSRGRKGRDTWHAMERVLREGDSAVTAIAVSVEARVVYVGSSDGAVTHWQWRRGAAPGAAPRNGGALRGHKMAVLCLAVAGRVVVSGSADRTISVWRREEGADHARLAVLRGHTGPVKCVAMDEEEEEDGAAGAPRRWVVYSGSLDGSVKVWRVSESDGGAHEDAMTPARTPAAARAWKGASPSPLRAWTPYAATPEPKHMGAAWWDRDVDALACVVAIWSVTTSAARGPGGG

>SiWD199

MAATRKKLIHFLRADPAAVSAASSAMSSPRSFSSNDSSVSDDDGYDNNSSSSSSFPASASSSPSRYSPPKSPWAATTHLPGLGGDSVDPTATGLIASLVKEDGKVYSLAAAGDVLYTGTDSENVRVWRDRRELAGFRTGSGLVKAIVVAADGRIFTGHQDGKVRVWRAGAGGDPAAHRRVGSLPALGDYLVSSVNPSSYVAKGGGRRRRAVWLRHSDAVSCLSLDEAAGLLYSGSWDRTFKVWRVSDSRCLESVPAHDDAVNTVAAAGFGGLVLTGSADGTVKVWRREAAVASGDRTRHVLERVLREGDGAVTAVAACPEARAVYVGSSDGLVTCWRWGPGDGGEPRLAGVLTGHRMGVMCLAVYGRVVVSGSADRTLCVWRRDGDGVAGQHQHQYQHVRLAVLTGHTGPVKCVAVAADVADCGDYAEGERRFVVYSGSLDGSVKVWRLSEDRPLELTVPLMPLLESEAWTALPSPAQAWAPELKRVAAA

>SiWD167

MAGAPGVGPGAGAGGGGGGGGAAAGLKTYFKTPEGRYKLQYEKTHSAVLHYSHGGKTVSQLTVAYLKEKPSSQGSQPSTPSSSSGMRSAAARLLGTGNGSKALNFGGGNGASRAVAGSSRIGGGLGTSTSLGGSQGVANYDGKGTYIVFNAADTLFISDLNSQEKDPIKSIHFSNSNPLCHAFDPEAKEGHDLIIGMGSGDVYSMSLRQQLQDPGRKHVAAQHYNKGDKDGSSNGSRCTSVAWVPEREGIFVVSHTDGNLYVYDKNKDGNTDCTFPAVKDQSQFMVAHAKSSKSNPVARWHICQGSINAISFSPDGAYLATVGRDGYLRVFDFSKEQLIFGGRSYYGALLCCTWSSDGKYLLTGGEDDLVQVWSMDDRKIVAWGEGHNSWVSGVAFDSYWSPPSSDGNGENVYRFGSVGQDTQLLLWDLALDEIVVPLRHPSCASPTFSVGSPSAHWDNACPPTGVLQPSPRMRDVPKLSPLVAHRVHADPLSGLVFTNESILTICREGLIKIWARPDQSENNQQSNSSEFVLGSPVPKDRAITSSNKASGSSFKKPSSVLVT

>SiWD175

MASAGSGGGGGGGAGAGGLKTYFKTPEGRHKLQYEKTHSPAVVHYNHSGKTVSQMTVAYLKEKPAGQGSTPSTPSAGSGMRSAAARLLGTGNGSRALSFGSNGTSKAVSGSSRIGGGIGASTSASGSQGMANYDGKGTYIIFNTADTLFISDLNSHDKDPIKSIHFSSSNPLCHAFDPEAKDGHDLLVGVFSGDVYSMSLRQQLQDPGKKPVSYQHFINKDKDKDKDPSQGGAASSRCTCVAWVPEREGIFVVSHADGNLYVYDKSKDGNTDWTFPTVKDQSQLLISHAKSSKGNPIARWHICQGAINAISFSPDGAYLATVGRDGYLRVFDFAKEQLIFGGKSYYGALLCCSWSADGKYLLSGGEDDLVQVWSMDDRKIVAWGEGHNSWVSTVAFDPYWSPPNSDETEENVMYRFGSVGQDTQLLLWDLALDEIAVPLRHPSAGSPTFSSGSPSAHWDNACPPTGVLQPSPQMRDVPKLSPLVAHRVHVDPLSGLEFTSESILTICREGLIKIWARPSHSETNQQPDSSEQIVGNTTVKDKMLTSSNKAGATSSSFKQPSSVLFT

>SiWD170

MGRTIKKAKKAKSKKTKKVEASSSSNPVVASGPAKVWQPGVDALEDGEELQFDPEAYNYLRGFSIGWSCLSFDVVRDQLGLVRSEFPHTFYGVAGTQAEKAPWNYIGIFKLSNISGKKREPIPPSAVDGDTDVDSDSSSDEEDEEINEDTKPILHLKKVAHAGCVNRIRSMTQKPHICATWGDTGHVQVWDLSSFLNSLAESGAPAPKEDDIIHKHLPVKVFSGHKDEGYAIDWSPLVAGRLVSGDCNKCIHLWEPTPTNWNVDPNPFVGHSASVEDLQWSPTEADIFASCSVDGTIAIWDIRTGKKPRMSVKAHKTDVNVISWNRLASCMIASGCDDGSFSVRDLRSIQEDSLVAHFEYHKKAITSIEWSPHEASSLAVTSEDHQLTIWDLSLERDAEEEAEFRAKMKEQANAPEDLPPQLLFVHQGQRDLKELHWHPQIPSMIVSTAIDGFNVLMPSNIDTTIPGNTDATMASAEP

>SiWD173

MGRTIKKAKSKKTKKVEASSSSNPAVASGPAKVWQPGVDALEDGEELQFDPEAYNYLRGFSIGWSCLSFDVVRDQLGLVRSEFPHTFYGVAGTQAEKAPWNYIGIFKLSNISGKKREPIPASAVDGDTDVDSDSSSDEEDEEINEDTKPVLHLKKVAHAGCVNRIRSMTQKPHLCATWGDTGHVQVWDLSSFLNSLAESGAPAPKEDDIIHKHLPVKVFSGHKDEGYAIDWSPLVTGRLVSGDCNKCIHLWEPTPTNWNVDANPFVGHSASVEDLQWSPTEADIFASCSVDGTIAIWDIRTGKKPRMSVKAHKTDVNVISWNRLASCMIASGCDDGSFSVRDLRSIQEDSLVAHFEYHKKAITSIEWSPHEASSLAVTSEDHQLTIWDLSLERDAEEEAEFRAKMKEQANAPEDLPPQLLFVHQGQRDLKELHWHPQIPSMIISTAIDGFNVLMPSNIDTTIPGNTDAAMASAEP
