# Supplementary material for: Genome-Wide Investigation and Expression Analyses of WD40 Protein Family in the Model Plant Foxtail Millet (Setaria italica L.)
Source: PLoS One. 2014 Jan 23;9(1):e86852. doi: 10.1371/journal.pone.0086852 (PMC3900672; doi:10.1371/journal.pone.0086852)
Supplement: Table S7 — The Ka/Ks ratios and estimated divergence time for orthologous WD proteins between foxtail millet, rice, sorghum and maize. (DOC) [file pone.0086852.s010.doc]

**Table S7.** The Ka/Ks ratios and estimated divergence time for orthologous WD proteins between foxtail millet, rice, sorghum and maize.

| **Foxtail millet - rice** | | | | | | | | |
| --- | --- | --- | --- | --- | --- | --- | --- | --- |
| **NIPGR ID** | **Chromosome** | **Gene IDs** | **Chromosome** | **Positions** | **Ks** | **Ka** | **Ka/Ks** | **Mya** |
| SiWD002 | SiChr1 | LOC_Os02g14790 | OsChr2 | 8204968 | 0.43 | 0.24 | 0.56 | 33.1 |
| SiWD003 | SiChr1 | LOC_Os02g13110 | OsChr2 | 6963959 | 0.46 | 0.27 | 0.59 | 35.4 |
| SiWD007 | SiChr1 | LOC_Os01g72220 | OsChr1 | 41873949 | 0.44 | 0.24 | 0.55 | 33.8 |
| SiWD008 | SiChr1 | LOC_Os02g04480 | OsChr2 | 1979693 | 0.45 | 0.29 | 0.64 | 34.6 |
| SiWD009 | SiChr1 | LOC_Os02g04440 | OsChr2 | 1955268 | 0.45 | 0.3 | 0.67 | 34.6 |
| SiWD010 | SiChr1 | LOC_Os02g03220 | OsChr2 | 1283277 | 0.44 | 0.26 | 0.59 | 33.8 |
| SiWD012 | SiChr1 | LOC_Os09g09470 | OsChr9 | 5094691 | 0.46 | 0.27 | 0.59 | 35.4 |
| SiWD014 | SiChr1 | LOC_Os02g32430 | OsChr2 | 19177089 | 0.45 | 0.26 | 0.58 | 34.6 |
| SiWD015 | SiChr1 | LOC_Os02g33630 | OsChr2 | 20026639 | 0.44 | 0.26 | 0.59 | 33.8 |
| SiWD016 | SiChr1 | LOC_Os02g33860 | OsChr2 | 20182213 | 0.46 | 0.3 | 0.65 | 35.4 |
| SiWD018 | SiChr1 | LOC_Os02g37856 | OsChr2 | 22872113 | 0.46 | 0.28 | 0.61 | 35.4 |
| SiWD022 | SiChr1 | LOC_Os02g45480 | OsChr2 | 27662418 | 0.45 | 0.3 | 0.67 | 34.6 |
| SiWD026 | SiChr1 | LOC_Os02g49090 | OsChr2 | 30015996 | 0.44 | 0.26 | 0.59 | 33.8 |
| SiWD027 | SiChr1 | LOC_Os02g50740 | OsChr2 | 30986860 | 0.43 | 0.29 | 0.67 | 33.1 |
| SiWD028 | SiChr1 | LOC_Os02g53140 | OsChr2 | 32528037 | 0.45 | 0.26 | 0.58 | 34.6 |
| SiWD029 | SiChr1 | LOC_Os02g54490 | OsChr2 | 33364263 | 0.46 | 0.26 | 0.57 | 35.4 |
| SiWD030 | SiChr1 | LOC_Os02g54910 | OsChr2 | 33629327 | 0.47 | 0.27 | 0.57 | 36.2 |
| SiWD031 | SiChr1 | LOC_Os02g55340 | OsChr2 | 33885845 | 0.44 | 0.3 | 0.68 | 33.8 |
| SiWD032 | SiChr1 | LOC_Os02g56880 | OsChr2 | 34862547 | 0.46 | 0.26 | 0.57 | 35.4 |
| SiWD033 | SiChr1 | LOC_Os02g57220 | OsChr2 | 35056930 | 0.45 | 0.26 | 0.58 | 34.6 |
| SiWD034 | SiChr2 | LOC_Os07g01730 | OsChr7 | 432084 | 0.47 | 0.3 | 0.64 | 36.2 |
| SiWD036 | SiChr2 | LOC_Os07g03160 | OsChr7 | 1228871 | 0.44 | 0.28 | 0.64 | 33.8 |
| SiWD037 | SiChr2 | LOC_Os08g21660 | OsChr8 | 12906720 | 0.46 | 0.29 | 0.63 | 35.4 |
| SiWD040 | SiChr2 | LOC_Os07g14280 | OsChr7 | 8143510 | 0.47 | 0.3 | 0.64 | 36.2 |
| SiWD041 | SiChr2 | LOC_Os07g14830 | OsChr7 | 8477403 | 0.43 | 0.28 | 0.65 | 33.1 |
| SiWD044 | SiChr2 | LOC_Os09g12710 | OsChr9 | 7271228 | 0.49 | 0.27 | 0.55 | 37.7 |
| SiWD045 | SiChr2 | LOC_Os09g19900 | OsChr9 | 11909038 | 0.44 | 0.25 | 0.57 | 33.8 |
| SiWD047 | SiChr2 | LOC_Os09g36900 | OsChr9 | 21276447 | 0.52 | 0.3 | 0.58 | 40.0 |
| SiWD048 | SiChr2 | LOC_Os07g38430 | OsChr7 | 23091890 | 0.49 | 0.28 | 0.57 | 37.7 |
| SiWD050 | SiChr2 | LOC_Os03g26870 | OsChr3 | 15329520 | 0.43 | 0.28 | 0.65 | 33.1 |
| SiWD051 | SiChr2 | LOC_Os07g46370 | OsChr7 | 27655956 | 0.5 | 0.29 | 0.58 | 38.5 |
| SiWD052 | SiChr2 | LOC_Os07g49300 | OsChr7 | 29522442 | 0.5 | 0.28 | 0.56 | 38.5 |
| SiWD053 | SiChr3 | LOC_Os04g58180 | OsChr4 | 34649540 | 0.44 | 0.28 | 0.64 | 33.8 |
| SiWD054 | SiChr3 | LOC_Os04g58130 | OsChr4 | 34613664 | 0.49 | 0.28 | 0.57 | 37.7 |
| SiWD055 | SiChr3 | LOC_Os04g11880 | OsChr4 | 6511575 | 0.43 | 0.27 | 0.63 | 33.1 |
| SiWD056 | SiChr3 | LOC_Os05g05210 | OsChr5 | 2536554 | 0.5 | 0.28 | 0.56 | 38.5 |
| SiWD060 | SiChr3 | LOC_Os05g23430 | OsChr5 | 13384950 | 0.49 | 0.26 | 0.53 | 37.7 |
| SiWD061 | SiChr3 | LOC_Os11g38170 | OsChr11 | 22630975 | 0.44 | 0.26 | 0.59 | 33.8 |
| SiWD063 | SiChr3 | LOC_Os05g49590 | OsChr5 | 28444997 | 0.5 | 0.3 | 0.60 | 38.5 |
| SiWD064 | SiChr3 | LOC_Os08g07960 | OsChr8 | 4496679 | 0.45 | 0.26 | 0.58 | 34.6 |
| SiWD066 | SiChr3 | LOC_Os05g46570 | OsChr5 | 26962970 | 0.49 | 0.3 | 0.61 | 37.7 |
| SiWD068 | SiChr3 | LOC_Os05g44320 | OsChr5 | 25788753 | 0.5 | 0.26 | 0.52 | 38.5 |
| SiWD069 | SiChr3 | LOC_Os05g34880 | OsChr5 | 20721494 | 0.44 | 0.26 | 0.59 | 33.8 |
| SiWD070 | SiChr3 | LOC_Os05g33710 | OsChr5 | 19837789 | 0.54 | 0.26 | 0.48 | 41.5 |
| SiWD071 | SiChr3 | LOC_Os05g33610 | OsChr5 | 19768780 | 0.54 | 0.3 | 0.56 | 41.5 |
| SiWD072 | SiChr3 | LOC_Os05g30010 | OsChr5 | 17361511 | 0.44 | 0.31 | 0.70 | 33.8 |
| SiWD073 | SiChr3 | LOC_Os07g40030 | OsChr7 | 24020377 | 0.54 | 0.26 | 0.48 | 41.5 |
| SiWD074 | SiChr3 | LOC_Os12g19590 | OsChr12 | 11419980 | 0.54 | 0.31 | 0.57 | 41.5 |
| SiWD075 | SiChr3 | LOC_Os12g40260 | OsChr12 | 24915510 | 0.44 | 0.27 | 0.61 | 33.8 |
| SiWD076 | SiChr3 | LOC_Os07g40930 | OsChr7 | 24493702 | 0.54 | 0.31 | 0.57 | 41.5 |
| SiWD077 | SiChr3 | LOC_Os12g41620 | OsChr12 | 25752101 | 0.49 | 0.31 | 0.63 | 37.7 |
| SiWD078 | SiChr3 | LOC_Os12g42150 | OsChr12 | 26128587 | 0.54 | 0.3 | 0.56 | 41.5 |
| SiWD079 | SiChr4 | LOC_Os06g03780 | OsChr6 | 1504211 | 0.43 | 0.31 | 0.72 | 33.1 |
| SiWD080 | SiChr4 | LOC_Os06g04040 | OsChr6 | 1648595 | 0.57 | 0.29 | 0.51 | 43.8 |
| SiWD082 | SiChr4 | LOC_Os06g07540 | OsChr6 | 3632663 | 0.43 | 0.3 | 0.70 | 33.1 |
| SiWD084 | SiChr4 | LOC_Os06g13140 | OsChr6 | 7209516 | 0.46 | 0.31 | 0.67 | 35.4 |
| SiWD087 | SiChr4 | LOC_Os04g52870 | OsChr4 | 31485364 | 0.57 | 0.25 | 0.44 | 43.8 |
| SiWD088 | SiChr4 | LOC_Os06g19660 | OsChr6 | 11219921 | 0.46 | 0.27 | 0.59 | 35.4 |
| SiWD089 | SiChr4 | LOC_Os06g22550 | OsChr6 | 13101544 | 0.57 | 0.24 | 0.42 | 43.8 |
| SiWD090 | SiChr4 | LOC_Os06g44370 | OsChr6 | 26778838 | 0.49 | 0.27 | 0.55 | 37.7 |
| SiWD091 | SiChr4 | LOC_Os06g44030 | OsChr6 | 26529298 | 0.57 | 0.26 | 0.46 | 43.8 |
| SiWD092 | SiChr4 | LOC_Os06g43690 | OsChr6 | 26303837 | 0.57 | 0.28 | 0.49 | 43.8 |
| SiWD093 | SiChr4 | LOC_Os06g39760 | OsChr6 | 23613102 | 0.45 | 0.29 | 0.64 | 34.6 |
| SiWD095 | SiChr4 | LOC_Os06g36770 | OsChr6 | 21654897 | 0.57 | 0.27 | 0.47 | 43.8 |
| SiWD096 | SiChr4 | LOC_Os02g06720 | OsChr2 | 3362047 | 0.46 | 0.27 | 0.59 | 35.4 |
| SiWD097 | SiChr4 | LOC_Os07g41190 | OsChr7 | 24660133 | 0.57 | 0.24 | 0.42 | 43.8 |
| SiWD098 | SiChr4 | LOC_Os06g50880 | OsChr6 | 30776898 | 0.57 | 0.26 | 0.46 | 43.8 |
| SiWD100 | SiChr5 | LOC_Os01g13730 | OsChr1 | 7679030 | 0.43 | 0.26 | 0.60 | 33.1 |
| SiWD101 | SiChr5 | LOC_Os01g13140 | OsChr1 | 7307881 | 0.55 | 0.26 | 0.47 | 42.3 |
| SiWD102 | SiChr5 | LOC_Os01g12930 | OsChr1 | 7177355 | 0.55 | 0.3 | 0.55 | 42.3 |
| SiWD103 | SiChr5 | LOC_Os01g08770 | OsChr1 | 4397652 | 0.44 | 0.25 | 0.57 | 33.8 |
| SiWD104 | SiChr5 | LOC_Os01g15020 | OsChr1 | 8420923 | 0.55 | 0.27 | 0.49 | 42.3 |
| SiWD105 | SiChr5 | LOC_Os01g03510 | OsChr1 | 1413839 | 0.43 | 0.26 | 0.60 | 33.1 |
| SiWD106 | SiChr5 | LOC_Os01g04870 | OsChr1 | 2250820 | 0.55 | 0.3 | 0.55 | 42.3 |
| SiWD107 | SiChr5 | LOC_Os01g07400 | OsChr1 | 3505665 | 0.55 | 0.24 | 0.44 | 42.3 |
| SiWD108 | SiChr5 | LOC_Os01g08190 | OsChr1 | 3975902 | 0.45 | 0.25 | 0.56 | 34.6 |
| SiWD109 | SiChr5 | LOC_Os01g09020 | OsChr1 | 4534021 | 0.51 | 0.28 | 0.55 | 39.2 |
| SiWD110 | SiChr5 | LOC_Os09g39420 | OsChr9 | 22666306 | 0.49 | 0.25 | 0.51 | 37.7 |
| SiWD111 | SiChr5 | LOC_Os01g09252 | OsChr1 | 4688108 | 0.51 | 0.27 | 0.53 | 39.2 |
| SiWD112 | SiChr5 | LOC_Os01g10790 | OsChr1 | 5760481 | 0.44 | 0.25 | 0.57 | 33.8 |
| SiWD113 | SiChr5 | LOC_Os07g22534 | OsChr7 | 12676090 | 0.51 | 0.26 | 0.51 | 39.2 |
| SiWD114 | SiChr5 | LOC_Os02g04320 | OsChr2 | 1902990 | 0.49 | 0.25 | 0.51 | 37.7 |
| SiWD115 | SiChr5 | LOC_Os01g21940 | OsChr1 | 12303170 | 0.48 | 0.26 | 0.54 | 36.9 |
| SiWD116 | SiChr5 | LOC_Os01g22390 | OsChr1 | 12584652 | 0.48 | 0.25 | 0.52 | 36.9 |
| SiWD117 | SiChr5 | LOC_Os01g28680 | OsChr1 | 16046649 | 0.48 | 0.25 | 0.52 | 36.9 |
| SiWD118 | SiChr5 | LOC_Os01g37120 | OsChr1 | 20717034 | 0.43 | 0.25 | 0.58 | 33.1 |
| SiWD119 | SiChr5 | LOC_Os01g39380 | OsChr1 | 22163096 | 0.47 | 0.29 | 0.62 | 36.2 |
| SiWD120 | SiChr5 | LOC_Os01g41630 | OsChr1 | 23569779 | 0.47 | 0.25 | 0.53 | 36.2 |
| SiWD122 | SiChr5 | LOC_Os01g42260 | OsChr1 | 23938369 | 0.45 | 0.27 | 0.60 | 34.6 |
| SiWD123 | SiChr5 | LOC_Os01g43250 | OsChr1 | 24703771 | 0.46 | 0.27 | 0.59 | 35.4 |
| SiWD124 | SiChr5 | LOC_Os01g44394 | OsChr1 | 25466363 | 0.45 | 0.27 | 0.60 | 34.6 |
| SiWD125 | SiChr5 | LOC_Os01g46510 | OsChr1 | 26453782 | 0.44 | 0.27 | 0.61 | 33.8 |
| SiWD126 | SiChr5 | LOC_Os02g18820 | OsChr2 | 10972703 | 0.45 | 0.27 | 0.60 | 34.6 |
| SiWD127 | SiChr5 | LOC_Os01g49290 | OsChr1 | 28330800 | 0.44 | 0.26 | 0.59 | 33.8 |
| SiWD128 | SiChr5 | LOC_Os01g50690 | OsChr1 | 29109936 | 0.43 | 0.3 | 0.70 | 33.1 |
| SiWD129 | SiChr5 | LOC_Os01g51300 | OsChr1 | 29501122 | 0.48 | 0.26 | 0.54 | 36.9 |
| SiWD130 | SiChr5 | LOC_Os01g52640 | OsChr1 | 30257752 | 0.45 | 0.3 | 0.67 | 34.6 |
| SiWD131 | SiChr5 | LOC_Os01g56860 | OsChr1 | 32838523 | 0.51 | 0.3 | 0.59 | 39.2 |
| SiWD132 | SiChr5 | LOC_Os01g57210 | OsChr1 | 33050635 | 0.5 | 0.22 | 0.44 | 38.5 |
| SiWD133 | SiChr5 | LOC_Os01g57720 | OsChr1 | 33368690 | 0.5 | 0.3 | 0.60 | 38.5 |
| SiWD134 | SiChr5 | LOC_Os02g20430 | OsChr2 | 12054833 | 0.49 | 0.3 | 0.61 | 37.7 |
| SiWD135 | SiChr5 | LOC_Os07g27140 | OsChr7 | 15732601 | 0.49 | 0.22 | 0.45 | 37.7 |
| SiWD136 | SiChr5 | LOC_Os01g69970 | OsChr1 | 40463007 | 0.51 | 0.3 | 0.59 | 39.2 |
| SiWD137 | SiChr5 | LOC_Os01g70780 | OsChr1 | 40966659 | 0.48 | 0.23 | 0.48 | 36.9 |
| SiWD138 | SiChr5 | LOC_Os07g46620 | OsChr7 | 27850797 | 0.44 | 0.22 | 0.50 | 33.8 |
| SiWD139 | SiChr5 | LOC_Os01g71780 | OsChr1 | 41582224 | 0.5 | 0.27 | 0.54 | 38.5 |
| SiWD140 | SiChr5 | LOC_Os01g74146 | OsChr1 | 42949414 | 0.5 | 0.23 | 0.46 | 38.5 |
| SiWD141 | SiChr6 | LOC_Os08g01680 | OsChr8 | 395228 | 0.46 | 0.3 | 0.65 | 35.4 |
| SiWD142 | SiChr6 | LOC_Os08g06480 | OsChr8 | 3667092 | 0.48 | 0.23 | 0.48 | 36.9 |
| SiWD143 | SiChr6 | LOC_Os08g04270 | OsChr8 | 2077234 | 0.46 | 0.3 | 0.65 | 35.4 |
| SiWD144 | SiChr6 | LOC_Os02g19210 | OsChr2 | 11199425 | 0.46 | 0.23 | 0.50 | 35.4 |
| SiWD145 | SiChr6 | LOC_Os08g18150 | OsChr8 | 11126801 | 0.51 | 0.25 | 0.49 | 39.2 |
| SiWD147 | SiChr6 | LOC_Os07g25440 | OsChr7 | 14534478 | 0.46 | 0.23 | 0.50 | 35.4 |
| SiWD148 | SiChr6 | LOC_Os08g31560 | OsChr8 | 19522096 | 0.53 | 0.29 | 0.55 | 40.8 |
| SiWD149 | SiChr6 | LOC_Os08g38570 | OsChr8 | 24384370 | 0.53 | 0.3 | 0.57 | 40.8 |
| SiWD150 | SiChr6 | LOC_Os08g38880 | OsChr8 | 24575562 | 0.49 | 0.25 | 0.51 | 37.7 |
| SiWD151 | SiChr6 | LOC_Os08g41270 | OsChr8 | 26062466 | 0.56 | 0.3 | 0.54 | 43.1 |
| SiWD152 | SiChr6 | LOC_Os08g41900 | OsChr8 | 26452131 | 0.43 | 0.25 | 0.58 | 33.1 |
| SiWD153 | SiChr6 | LOC_Os03g48090 | OsChr3 | 27347078 | 0.57 | 0.27 | 0.47 | 43.8 |
| SiWD154 | SiChr6 | LOC_Os08g44330 | OsChr8 | 27893237 | 0.49 | 0.26 | 0.53 | 37.7 |
| SiWD155 | SiChr6 | LOC_Os08g44010 | OsChr8 | 27703403 | 0.54 | 0.25 | 0.46 | 41.5 |
| SiWD156 | SiChr7 | LOC_Os07g22220 | OsChr7 | 12424060 | 0.54 | 0.25 | 0.46 | 41.5 |
| SiWD157 | SiChr7 | LOC_Os09g06560 | OsChr9 | 3118920 | 0.45 | 0.25 | 0.56 | 34.6 |
| SiWD158 | SiChr7 | LOC_Os04g34080 | OsChr4 | 20640334 | 0.53 | 0.26 | 0.49 | 40.8 |
| SiWD159 | SiChr7 | LOC_Os04g42880 | OsChr4 | 25374638 | 0.51 | 0.25 | 0.49 | 39.2 |
| SiWD160 | SiChr7 | LOC_Os02g42590 | OsChr2 | 25619021 | 0.52 | 0.29 | 0.56 | 40.0 |
| SiWD161 | SiChr7 | LOC_Os04g46894 | OsChr4 | 27799654 | 0.52 | 0.3 | 0.58 | 40.0 |
| SiWD162 | SiChr7 | LOC_Os04g48010 | OsChr4 | 28563829 | 0.49 | 0.29 | 0.59 | 37.7 |
| SiWD163 | SiChr7 | LOC_Os04g50660 | OsChr4 | 29981604 | 0.47 | 0.29 | 0.62 | 36.2 |
| SiWD164 | SiChr7 | LOC_Os02g47180 | OsChr2 | 28802793 | 0.44 | 0.27 | 0.61 | 33.8 |
| SiWD165 | SiChr7 | LOC_Os07g12320 | OsChr7 | 6949934 | 0.44 | 0.27 | 0.61 | 33.8 |
| SiWD166 | SiChr7 | LOC_Os12g07874 | OsChr12 | 3982946 | 0.45 | 0.25 | 0.56 | 34.6 |
| SiWD168 | SiChr7 | LOC_Os12g06810 | OsChr12 | 3305189 | 0.46 | 0.27 | 0.59 | 35.4 |
| SiWD169 | SiChr7 | LOC_Os12g03540 | OsChr12 | 1405136 | 0.47 | 0.27 | 0.57 | 36.2 |
| SiWD173 | SiChr8 | LOC_Os11g03990 | OsChr11 | 1610886 | 0.48 | 0.25 | 0.52 | 36.9 |
| SiWD174 | SiChr8 | LOC_Os12g01922 | OsChr12 | 541268 | 0.46 | 0.28 | 0.61 | 35.4 |
| SiWD175 | SiChr8 | LOC_Os12g07450 | OsChr12 | 3672805 | 0.49 | 0.3 | 0.61 | 37.7 |
| SiWD176 | SiChr8 | LOC_Os11g07970 | OsChr11 | 4134329 | 0.5 | 0.29 | 0.58 | 38.5 |
| SiWD177 | SiChr8 | LOC_Os11g08400 | OsChr11 | 4427965 | 0.56 | 0.3 | 0.54 | 43.1 |
| SiWD178 | SiChr8 | LOC_Os02g11830 | OsChr2 | 6119039 | 0.55 | 0.29 | 0.53 | 42.3 |
| SiWD179 | SiChr8 | LOC_Os11g39650 | OsChr11 | 23615037 | 0.43 | 0.29 | 0.67 | 33.1 |
| SiWD180 | SiChr8 | LOC_Os11g43890 | OsChr11 | 26501618 | 0.51 | 0.3 | 0.59 | 39.2 |
| SiWD181 | SiChr9 | LOC_Os03g64300 | OsChr3 | 36340083 | 0.48 | 0.27 | 0.56 | 36.9 |
| SiWD182 | SiChr9 | LOC_Os03g64110 | OsChr3 | 36228699 | 0.46 | 0.27 | 0.59 | 35.4 |
| SiWD183 | SiChr9 | LOC_Os03g14615 | OsChr3 | 7938858 | 0.46 | 0.27 | 0.59 | 35.4 |
| SiWD184 | SiChr9 | LOC_Os07g09000 | OsChr7 | 4682480 | 0.51 | 0.27 | 0.53 | 39.2 |
| SiWD185 | SiChr9 | LOC_Os03g54770 | OsChr3 | 31134469 | 0.45 | 0.28 | 0.62 | 34.6 |
| SiWD186 | SiChr9 | LOC_Os03g53530 | OsChr3 | 30701238 | 0.46 | 0.28 | 0.61 | 35.4 |
| SiWD187 | SiChr9 | LOC_Os03g53510 | OsChr3 | 30689440 | 0.51 | 0.26 | 0.51 | 39.2 |
| SiWD188 | SiChr9 | LOC_Os03g53280 | OsChr3 | 30564184 | 0.48 | 0.28 | 0.58 | 36.9 |
| SiWD189 | SiChr9 | LOC_Os03g52870 | OsChr3 | 30319464 | 0.46 | 0.28 | 0.61 | 35.4 |
| SiWD190 | SiChr9 | LOC_Os03g52794 | OsChr3 | 30262476 | 0.46 | 0.26 | 0.57 | 35.4 |
| SiWD191 | SiChr9 | LOC_Os03g52470 | OsChr3 | 30109909 | 0.51 | 0.28 | 0.55 | 39.2 |
| SiWD192 | SiChr9 | LOC_Os03g51550 | OsChr3 | 29486017 | 0.55 | 0.3 | 0.55 | 42.3 |
| SiWD193 | SiChr9 | LOC_Os09g04110 | OsChr9 | 2126232 | 0.44 | 0.25 | 0.57 | 33.8 |
| SiWD194 | SiChr9 | LOC_Os03g49200 | OsChr3 | 28023221 | 0.46 | 0.25 | 0.54 | 35.4 |
| SiWD195 | SiChr9 | LOC_Os03g47780 | OsChr3 | 27120029 | 0.51 | 0.29 | 0.57 | 39.2 |
| SiWD196 | SiChr9 | LOC_Os03g46650 | OsChr3 | 26398523 | 0.46 | 0.25 | 0.54 | 35.4 |
| SiWD197 | SiChr9 | LOC_Os03g42770 | OsChr3 | 23817641 | 0.46 | 0.27 | 0.59 | 35.4 |
| SiWD198 | SiChr9 | LOC_Os03g43890 | OsChr3 | 24621292 | 0.51 | 0.25 | 0.49 | 39.2 |
| SiWD199 | SiChr9 | LOC_Os03g42710 | OsChr3 | 23781111 | 0.47 | 0.29 | 0.62 | 36.2 |
| SiWD200 | SiChr9 | LOC_Os09g24260 | OsChr9 | 14404531 | 0.47 | 0.3 | 0.64 | 36.2 |
| SiWD201 | SiChr9 | LOC_Os10g35200 | OsChr10 | 18798725 | 0.43 | 0.26 | 0.60 | 33.1 |
| SiWD202 | SiChr9 | LOC_Os03g21990 | OsChr3 | 12599467 | 0.47 | 0.22 | 0.47 | 36.2 |
| SiWD203 | SiChr9 | LOC_Os10g32880 | OsChr10 | 17203876 | 0.47 | 0.22 | 0.47 | 36.2 |
| SiWD204 | SiChr9 | LOC_Os10g32770 | OsChr10 | 17158103 | 0.47 | 0.22 | 0.47 | 36.2 |
| SiWD205 | SiChr9 | LOC_Os10g32710 | OsChr10 | 17120230 | 0.49 | 0.26 | 0.53 | 37.7 |
| SiWD206 | SiChr9 | LOC_Os12g03822 | OsChr12 | 1562101 | 0.52 | 0.29 | 0.56 | 40.0 |
| SiWD207 | SiChr9 | LOC_Os10g41370 | OsChr10 | 22236887 | 0.52 | 0.22 | 0.42 | 40.0 |
| SiWD208 | SiChr9 | LOC_Os10g39760 | OsChr10 | 21253567 | 0.53 | 0.26 | 0.49 | 40.8 |
| SiWD209 | SiChr9 | LOC_Os03g33580 | OsChr3 | 19183831 | 0.45 | 0.26 | 0.58 | 34.6 |
| SiWD210 | SiChr9 | LOC_Os02g21490 | OsChr2 | 12741603 | 0.5 | 0.26 | 0.52 | 38.5 |
| SiWD211 | SiChr9 | LOC_Os03g27970 | OsChr3 | 16065984 | 0.43 | 0.26 | 0.60 | 33.1 |
| SiWD212 | SiChr9 | LOC_Os03g23935 | OsChr3 | 13571003 | 0.47 | 0.3 | 0.64 | 36.2 |
| SiWD213 | SiChr9 | LOC_Os03g19340 | OsChr3 | 10866498 | 0.47 | 0.26 | 0.55 | 36.2 |
| SiWD214 | SiChr9 | LOC_Os03g18840 | OsChr3 | 10556232 | 0.44 | 0.27 | 0.61 | 33.8 |
| SiWD215 | SiChr9 | LOC_Os03g17780 | OsChr3 | 9893421 | 0.45 | 0.3 | 0.67 | 34.6 |
| SiWD216 | SiChr9 | LOC_Os02g48964 | OsChr2 | 29934369 | 0.46 | 0.3 | 0.65 | 35.4 |
| SiWD217 | SiChr9 | LOC_Os03g14980 | OsChr3 | 8165826 | 0.47 | 0.3 | 0.64 | 36.2 |
| SiWD218 | SiChr9 | LOC_Os03g10990 | OsChr3 | 5646147 | 0.45 | 0.3 | 0.67 | 34.6 |
| SiWD219 | SiChr9 | LOC_Os03g08830 | OsChr3 | 4561061 | 0.43 | 0.26 | 0.60 | 33.1 |
| SiWD220 | SiChr9 | LOC_Os03g05720 | OsChr3 | 2845709 | 0.43 | 0.27 | 0.63 | 33.1 |
| SiWD221 | SiChr9 | LOC_Os03g05210 | OsChr3 | 2528867 | 0.46 | 0.3 | 0.65 | 35.4 |
| SiWD222 | SiChr9 | LOC_Os03g03150 | OsChr3 | 1327397 | 0.46 | 0.3 | 0.65 | 35.4 |
| SiWD224 | SiChr9 | LOC_Os03g02440 | OsChr3 | 870491 | 0.49 | 0.29 | 0.59 | 37.7 |
| SiWD225 | SiChr9 | LOC_Os03g02110 | OsChr3 | 674687 | 0.45 | 0.3 | 0.67 | 34.6 |
| **Mean** | | | | | **0.49** | **0.27** | **0.55** | **37.7** |

| **Foxtail millet - maize** | | | | | | | | |
| --- | --- | --- | --- | --- | --- | --- | --- | --- |
| **NIPGR ID** | **Chromosome** | **Gene IDs** | **Chromosome** | **Positions** | **Ks** | **Ka** | **Ka/Ks** | **Mya** |
| SiWD001 | SiChr1 | GRMZM2G063192 | ZmChr4 | 159174834 | 0.26 | 0.07 | 0.27 | 20.0 |
| SiWD002 | SiChr1 | GRMZM2G159849 | ZmChr5 | 144888445 | 0.28 | 0.07 | 0.25 | 21.5 |
| SiWD003 | SiChr1 | GRMZM2G069631 | ZmChr4 | 229500502 | 0.29 | 0.06 | 0.21 | 22.3 |
| SiWD005 | SiChr1 | GRMZM2G022275 | ZmChr9 | 86866692 | 0.24 | 0.08 | 0.33 | 18.5 |
| SiWD006 | SiChr1 | GRMZM2G401869 | ZmChr4 | 236677619 | 0.24 | 0.09 | 0.38 | 18.5 |
| SiWD007 | SiChr1 | GRMZM5G881296 | ZmChr3 | 149228971 | 0.26 | 0.07 | 0.27 | 20.0 |
| SiWD008 | SiChr1 | GRMZM2G096972 | ZmChr5 | 77096208 | 0.26 | 0.07 | 0.27 | 20.0 |
| SiWD009 | SiChr1 | GRMZM2G399858 | ZmChr5 | 76914715 | 0.28 | 0.07 | 0.25 | 21.5 |
| SiWD010 | SiChr1 | GRMZM2G344212 | ZmChr5 | 72687409 | 0.29 | 0.06 | 0.21 | 22.3 |
| SiWD011 | SiChr1 | GRMZM2G366698 | ZmChr5 | 160709566 | 0.24 | 0.08 | 0.33 | 18.5 |
| SiWD012 | SiChr1 | GRMZM2G088261 | ZmChr2 | 161855179 | 0.24 | 0.09 | 0.38 | 18.5 |
| SiWD013 | SiChr1 | GRMZM2G096051 | ZmChr6 | 82567143 | 0.25 | 0.07 | 0.28 | 19.2 |
| SiWD014 | SiChr1 | GRMZM2G099334 | ZmChr5 | 167430904 | 0.26 | 0.07 | 0.27 | 20.0 |
| SiWD015 | SiChr1 | GRMZM2G048800 | ZmChr5 | 170813707 | 0.24 | 0.09 | 0.38 | 18.5 |
| SiWD016 | SiChr1 | GRMZM2G120784 | ZmChr4 | 113854312 | 0.24 | 0.09 | 0.38 | 18.5 |
| SiWD018 | SiChr1 | GRMZM2G113064 | ZmChr5 | 179277850 | 0.29 | 0.09 | 0.31 | 22.3 |
| SiWD019 | SiChr1 | GRMZM2G164185 | ZmChr8 | 141517149 | 0.28 | 0.07 | 0.25 | 21.5 |
| SiWD020 | SiChr1 | GRMZM2G071448 | ZmChr4 | 146531494 | 0.27 | 0.09 | 0.33 | 20.8 |
| SiWD021 | SiChr1 | GRMZM2G044143 | ZmChr2 | 18987884 | 0.27 | 0.08 | 0.30 | 20.8 |
| SiWD022 | SiChr1 | GRMZM2G037200 | ZmChr5 | 196017079 | 0.25 | 0.08 | 0.32 | 19.2 |
| SiWD024 | SiChr1 | GRMZM2G048045 | ZmChr4 | 163306755 | 0.24 | 0.08 | 0.33 | 18.5 |
| SiWD027 | SiChr1 | GRMZM2G123262 | ZmChr5 | 205942629 | 0.24 | 0.09 | 0.38 | 18.5 |
| SiWD028 | SiChr1 | GRMZM2G104920 | ZmChr5 | 210740579 | 0.26 | 0.07 | 0.27 | 20.0 |
| SiWD029 | SiChr1 | GRMZM2G053766 | ZmChr4 | 175572628 | 0.26 | 0.07 | 0.27 | 20.0 |
| SiWD030 | SiChr1 | GRMZM2G018573 | ZmChr5 | 213255642 | 0.28 | 0.07 | 0.25 | 21.5 |
| SiWD031 | SiChr1 | GRMZM2G179662 | ZmChr5 | 213757019 | 0.29 | 0.06 | 0.21 | 22.3 |
| SiWD032 | SiChr1 | GRMZM2G079013 | ZmChr5 | 215339270 | 0.24 | 0.08 | 0.33 | 18.5 |
| SiWD033 | SiChr1 | GRMZM2G178801 | ZmChr4 | 171441139 | 0.24 | 0.09 | 0.38 | 18.5 |
| SiWD034 | SiChr2 | GRMZM2G555108 | ZmChr7 | 1301454 | 0.25 | 0.07 | 0.28 | 19.2 |
| SiWD036 | SiChr2 | GRMZM2G044060 | ZmChr7 | 2997300 | 0.26 | 0.07 | 0.27 | 20.0 |
| SiWD037 | SiChr2 | GRMZM2G143330 | ZmChr7 | 7507005 | 0.24 | 0.09 | 0.38 | 18.5 |
| SiWD039 | SiChr2 | GRMZM2G158489 | ZmChr7 | 13610539 | 0.24 | 0.09 | 0.38 | 18.5 |
| SiWD040 | SiChr2 | GRMZM2G074567 | ZmChr2 | 170296225 | 0.29 | 0.09 | 0.31 | 22.3 |
| SiWD041 | SiChr2 | GRMZM2G320920 | ZmChr7 | 25505806 | 0.28 | 0.07 | 0.25 | 21.5 |
| SiWD042 | SiChr2 | GRMZM2G159330 | ZmChr10 | 101031429 | 0.27 | 0.09 | 0.33 | 20.8 |
| SiWD043 | SiChr2 | GRMZM2G023625 | ZmChr7 | 57624330 | 0.27 | 0.08 | 0.30 | 20.8 |
| SiWD044 | SiChr2 | GRMZM2G078754 | ZmChr7 | 82563213 | 0.24 | 0.08 | 0.33 | 18.5 |
| SiWD045 | SiChr2 | GRMZM2G004475 | ZmChr7 | 98407386 | 0.26 | 0.09 | 0.35 | 20.0 |
| SiWD047 | SiChr2 | GRMZM2G032711 | ZmChr7 | 175560055 | 0.26 | 0.08 | 0.31 | 20.0 |
| SiWD048 | SiChr2 | GRMZM2G019084 | ZmChr7 | 159476983 | 0.27 | 0.08 | 0.30 | 20.8 |
| SiWD049 | SiChr2 | GRMZM2G050501 | ZmChr7 | 163610898 | 0.24 | 0.09 | 0.38 | 18.5 |
| SiWD050 | SiChr2 | GRMZM2G078806 | ZmChr1 | 66012877 | 0.24 | 0.08 | 0.33 | 18.5 |
| SiWD051 | SiChr2 | GRMZM2G035985 | ZmChr2 | 214832665 | 0.26 | 0.07 | 0.27 | 20.0 |
| SiWD052 | SiChr2 | GRMZM2G434696 | ZmChr7 | 174775113 | 0.26 | 0.07 | 0.27 | 20.0 |
| SiWD053 | SiChr3 | GRMZM2G142667 | ZmChr10 | 148405004 | 0.26 | 0.07 | 0.27 | 20.0 |
| SiWD055 | SiChr3 | GRMZM2G040247 | ZmChr2 | 2040400 | 0.28 | 0.07 | 0.25 | 21.5 |
| SiWD056 | SiChr3 | GRMZM2G037350 | ZmChr8 | 128481827 | 0.29 | 0.06 | 0.21 | 22.3 |
| SiWD059 | SiChr3 | GRMZM2G158179 | ZmChr5 | 29271388 | 0.24 | 0.08 | 0.33 | 18.5 |
| SiWD061 | SiChr3 | GRMZM2G157034 | ZmChr6 | 168355070 | 0.24 | 0.09 | 0.38 | 18.5 |
| SiWD062 | SiChr3 | GRMZM2G145556 | ZmChr6 | 165393177 | 0.25 | 0.07 | 0.28 | 19.2 |
| SiWD064 | SiChr3 | GRMZM2G058498 | ZmChr10 | 49799362 | 0.26 | 0.07 | 0.27 | 20.0 |
| SiWD065 | SiChr3 | GRMZM2G040477 | ZmChr8 | 65749806 | 0.24 | 0.09 | 0.38 | 18.5 |
| SiWD066 | SiChr3 | GRMZM2G168752 | ZmChr8 | 63268755 | 0.24 | 0.09 | 0.38 | 18.5 |
| SiWD067 | SiChr3 | GRMZM2G105401 | ZmChr6 | 159588747 | 0.29 | 0.09 | 0.31 | 22.3 |
| SiWD068 | SiChr3 | GRMZM2G060817 | ZmChr6 | 160625203 | 0.28 | 0.07 | 0.25 | 21.5 |
| SiWD069 | SiChr3 | GRMZM2G309568 | ZmChr8 | 22501528 | 0.27 | 0.09 | 0.33 | 20.8 |
| SiWD070 | SiChr3 | GRMZM2G100152 | ZmChr8 | 106279610 | 0.27 | 0.08 | 0.30 | 20.8 |
| SiWD071 | SiChr3 | GRMZM2G078468 | ZmChr8 | 106938685 | 0.25 | 0.08 | 0.32 | 19.2 |
| SiWD072 | SiChr3 | GRMZM2G325804 | ZmChr6 | 138182751 | 0.29 | 0.08 | 0.28 | 22.3 |
| SiWD073 | SiChr3 | GRMZM2G032260 | ZmChr2 | 13869782 | 0.24 | 0.07 | 0.29 | 18.5 |
| SiWD074 | SiChr3 | GRMZM2G150631 | ZmChr3 | 210820925 | 0.25 | 0.07 | 0.28 | 19.2 |
| SiWD075 | SiChr3 | GRMZM2G368126 | ZmChr3 | 114790912 | 0.24 | 0.08 | 0.33 | 18.5 |
| SiWD077 | SiChr3 | GRMZM2G049201 | ZmChr1 | 172815913 | 0.26 | 0.09 | 0.35 | 20.0 |
| SiWD078 | SiChr3 | GRMZM2G327655 | ZmChr7 | 168165592 | 0.28 | 0.07 | 0.25 | 21.5 |
| SiWD079 | SiChr4 | GRMZM2G087196 | ZmChr9 | 10324769 | 0.27 | 0.09 | 0.33 | 20.8 |
| SiWD080 | SiChr4 | GRMZM2G396562 | ZmChr9 | 23535304 | 0.27 | 0.08 | 0.30 | 20.8 |
| SiWD081 | SiChr4 | GRMZM2G000645 | ZmChr9 | 20394189 | 0.25 | 0.08 | 0.32 | 19.2 |
| SiWD082 | SiChr4 | GRMZM2G161102 | ZmChr6 | 122544824 | 0.24 | 0.07 | 0.29 | 18.5 |
| SiWD086 | SiChr4 | GRMZM2G124886 | ZmChr5 | 157277567 | 0.25 | 0.07 | 0.28 | 19.2 |
| SiWD087 | SiChr4 | GRMZM2G029478 | ZmChr10 | 142821110 | 0.24 | 0.08 | 0.33 | 18.5 |
| SiWD088 | SiChr4 | GRMZM2G029186 | ZmChr5 | 204089494 | 0.26 | 0.09 | 0.35 | 20.0 |
| SiWD090 | SiChr4 | GRMZM5G829955 | ZmChr6 | 95473748 | 0.26 | 0.08 | 0.31 | 20.0 |
| SiWD091 | SiChr4 | GRMZM2G135300 | ZmChr9 | 103172885 | 0.27 | 0.08 | 0.30 | 20.8 |
| SiWD092 | SiChr4 | GRMZM2G087712 | ZmChr9 | 94004389 | 0.24 | 0.09 | 0.38 | 18.5 |
| SiWD094 | SiChr4 | GRMZM2G443953 | ZmChr9 | 87983910 | 0.28 | 0.07 | 0.25 | 21.5 |
| SiWD095 | SiChr4 | GRMZM2G165418 | ZmChr9 | 57824184 | 0.27 | 0.09 | 0.33 | 20.8 |
| SiWD096 | SiChr4 | GRMZM2G362726 | ZmChr6 | 91146137 | 0.27 | 0.08 | 0.30 | 20.8 |
| SiWD097 | SiChr4 | GRMZM2G099758 | ZmChr5 | 60520588 | 0.25 | 0.08 | 0.32 | 19.2 |
| SiWD098 | SiChr4 | GRMZM2G132644 | ZmChr6 | 83217298 | 0.28 | 0.07 | 0.25 | 21.5 |
| SiWD099 | SiChr5 | GRMZM2G458266 | ZmChr5 | 2524248 | 0.29 | 0.06 | 0.21 | 22.3 |
| SiWD100 | SiChr5 | GRMZM2G590033 | ZmChr1 | 299113982 | 0.24 | 0.08 | 0.33 | 18.5 |
| SiWD101 | SiChr5 | GRMZM2G432644 | ZmChr8 | 28590101 | 0.24 | 0.09 | 0.38 | 18.5 |
| SiWD102 | SiChr5 | GRMZM2G153127 | ZmChr3 | 2061189 | 0.25 | 0.07 | 0.28 | 19.2 |
| SiWD104 | SiChr5 | GRMZM2G316967 | ZmChr3 | 35794313 | 0.26 | 0.07 | 0.27 | 20.0 |
| SiWD105 | SiChr5 | GRMZM5G853361 | ZmChr3 | 24736562 | 0.24 | 0.09 | 0.38 | 18.5 |
| SiWD106 | SiChr5 | GRMZM2G126956 | ZmChr8 | 13615942 | 0.24 | 0.09 | 0.38 | 18.5 |
| SiWD107 | SiChr5 | GRMZM2G069177 | ZmChr3 | 13311963 | 0.29 | 0.09 | 0.31 | 22.3 |
| SiWD108 | SiChr5 | GRMZM2G361398 | ZmChr3 | 224119877 | 0.28 | 0.07 | 0.25 | 21.5 |
| SiWD109 | SiChr5 | GRMZM2G576002 | ZmChr3 | 8271085 | 0.27 | 0.09 | 0.33 | 20.8 |
| SiWD111 | SiChr5 | GRMZM2G152370 | ZmChr3 | 7607705 | 0.27 | 0.08 | 0.30 | 20.8 |
| SiWD112 | SiChr5 | GRMZM2G133314 | ZmChr3 | 5437230 | 0.25 | 0.08 | 0.32 | 19.2 |
| SiWD113 | SiChr5 | GRMZM2G128477 | ZmChr6 | 165077708 | 0.24 | 0.09 | 0.38 | 18.5 |
| SiWD114 | SiChr5 | GRMZM5G871980 | ZmChr5 | 47850984 | 0.26 | 0.09 | 0.35 | 20.0 |
| SiWD115 | SiChr5 | GRMZM2G037683 | ZmChr3 | 58293668 | 0.26 | 0.08 | 0.31 | 20.0 |
| SiWD116 | SiChr5 | GRMZM2G118641 | ZmChr3 | 59601036 | 0.27 | 0.08 | 0.30 | 20.8 |
| SiWD118 | SiChr5 | GRMZM2G464754 | ZmChr8 | 136285576 | 0.24 | 0.09 | 0.38 | 18.5 |
| SiWD119 | SiChr5 | GRMZM2G074572 | ZmChr3 | 219884844 | 0.28 | 0.07 | 0.25 | 21.5 |
| SiWD123 | SiChr5 | GRMZM2G057853 | ZmChr3 | 218004261 | 0.27 | 0.09 | 0.33 | 20.8 |
| SiWD124 | SiChr5 | GRMZM2G180815 | ZmChr3 | 216126407 | 0.27 | 0.08 | 0.30 | 20.8 |
| SiWD125 | SiChr5 | GRMZM2G000177 | ZmChr8 | 147431766 | 0.25 | 0.08 | 0.32 | 19.2 |
| SiWD126 | SiChr5 | GRMZM2G353548 | ZmChr2 | 89696000 | 0.28 | 0.07 | 0.25 | 21.5 |
| SiWD127 | SiChr5 | GRMZM2G038032 | ZmChr6 | 162882094 | 0.29 | 0.06 | 0.21 | 22.3 |
| SiWD129 | SiChr5 | GRMZM2G137965 | ZmChr3 | 204071388 | 0.24 | 0.08 | 0.33 | 18.5 |
| SiWD130 | SiChr5 | GRMZM2G061602 | ZmChr8 | 155895389 | 0.24 | 0.09 | 0.38 | 18.5 |
| SiWD131 | SiChr5 | GRMZM2G093239 | ZmChr3 | 191768789 | 0.25 | 0.07 | 0.28 | 19.2 |
| SiWD133 | SiChr5 | GRMZM2G116700 | ZmChr3 | 189896515 | 0.26 | 0.07 | 0.27 | 20.0 |
| SiWD134 | SiChr5 | GRMZM2G096802 | ZmChr3 | 177146570 | 0.24 | 0.09 | 0.38 | 18.5 |
| SiWD135 | SiChr5 | GRMZM2G396231 | ZmChr3 | 177123763 | 0.24 | 0.09 | 0.38 | 18.5 |
| SiWD136 | SiChr5 | GRMZM2G081013 | ZmChr3 | 158509335 | 0.29 | 0.09 | 0.31 | 22.3 |
| SiWD137 | SiChr5 | GRMZM2G122607 | ZmChr8 | 159703050 | 0.28 | 0.07 | 0.25 | 21.5 |
| SiWD139 | SiChr5 | GRMZM2G076034 | ZmChr3 | 151200898 | 0.27 | 0.09 | 0.33 | 20.8 |
| SiWD140 | SiChr5 | GRMZM5G821639 | ZmChr3 | 144686962 | 0.27 | 0.08 | 0.30 | 20.8 |
| SiWD141 | SiChr6 | GRMZM2G009627 | ZmChr6 | 2038613 | 0.25 | 0.08 | 0.32 | 19.2 |
| SiWD142 | SiChr6 | GRMZM2G042992 | ZmChr10 | 76636684 | 0.24 | 0.09 | 0.38 | 18.5 |
| SiWD143 | SiChr6 | GRMZM2G148924 | ZmChr10 | 84092833 | 0.27 | 0.08 | 0.30 | 20.8 |
| SiWD144 | SiChr6 | GRMZM2G061186 | ZmChr6 | 8380046 | 0.28 | 0.09 | 0.32 | 21.5 |
| SiWD145 | SiChr6 | GRMZM5G817651 | ZmChr1 | 144231224 | 0.29 | 0.08 | 0.28 | 22.3 |
| SiWD147 | SiChr6 | GRMZM2G094959 | ZmChr2 | 228080020 | 0.24 | 0.07 | 0.29 | 18.5 |
| SiWD148 | SiChr6 | GRMZM2G144547 | ZmChr1 | 217520817 | 0.25 | 0.07 | 0.28 | 19.2 |
| SiWD149 | SiChr6 | GRMZM2G042371 | ZmChr1 | 183447693 | 0.24 | 0.08 | 0.33 | 18.5 |
| SiWD150 | SiChr6 | GRMZM2G113726 | ZmChr1 | 183801615 | 0.27 | 0.08 | 0.30 | 20.8 |
| SiWD151 | SiChr6 | GRMZM2G065225 | ZmChr4 | 42811395 | 0.28 | 0.09 | 0.32 | 21.5 |
| SiWD152 | SiChr6 | GRMZM2G014612 | ZmChr4 | 44177884 | 0.29 | 0.08 | 0.28 | 22.3 |
| SiWD153 | SiChr6 | GRMZM2G168428 | ZmChr8 | 156616216 | 0.24 | 0.07 | 0.29 | 18.5 |
| SiWD154 | SiChr6 | GRMZM2G049525 | ZmChr4 | 53440616 | 0.25 | 0.07 | 0.28 | 19.2 |
| SiWD155 | SiChr6 | GRMZM2G081538 | ZmChr4 | 90840893 | 0.24 | 0.08 | 0.33 | 18.5 |
| SiWD156 | SiChr7 | GRMZM2G149708 | ZmChr2 | 104729978 | 0.27 | 0.08 | 0.30 | 20.8 |
| SiWD157 | SiChr7 | GRMZM2G179424 | ZmChr7 | 62623716 | 0.28 | 0.09 | 0.32 | 21.5 |
| SiWD158 | SiChr7 | GRMZM2G055437 | ZmChr10 | 115146360 | 0.29 | 0.08 | 0.28 | 22.3 |
| SiWD159 | SiChr7 | GRMZM2G077458 | ZmChr10 | 129511443 | 0.27 | 0.08 | 0.30 | 20.8 |
| SiWD160 | SiChr7 | GRMZM2G027049 | ZmChr2 | 23707011 | 0.28 | 0.09 | 0.32 | 21.5 |
| SiWD162 | SiChr7 | GRMZM2G125656 | ZmChr10 | 139316025 | 0.29 | 0.08 | 0.28 | 22.3 |
| SiWD163 | SiChr7 | GRMZM2G128092 | ZmChr10 | 139995082 | 0.29 | 0.08 | 0.28 | 22.3 |
| SiWD166 | SiChr7 | GRMZM2G319435 | ZmChr10 | 10666472 | 0.24 | 0.07 | 0.29 | 18.5 |
| SiWD167 | SiChr7 | GRMZM2G096070 | ZmChr3 | 140725960 | 0.25 | 0.07 | 0.28 | 19.2 |
| SiWD168 | SiChr7 | GRMZM2G167999 | ZmChr10 | 8704585 | 0.24 | 0.08 | 0.33 | 18.5 |
| SiWD169 | SiChr7 | GRMZM2G180205 | ZmChr10 | 2043909 | 0.26 | 0.09 | 0.35 | 20.0 |
| SiWD172 | SiChr7 | GRMZM2G048067 | ZmChr10 | 1411449 | 0.26 | 0.08 | 0.31 | 20.0 |
| SiWD176 | SiChr8 | GRMZM2G087105 | ZmChr2 | 130950189 | 0.27 | 0.08 | 0.30 | 20.8 |
| SiWD177 | SiChr8 | GRMZM2G375856 | ZmChr4 | 208809013 | 0.28 | 0.09 | 0.32 | 21.5 |
| SiWD178 | SiChr8 | GRMZM2G122038 | ZmChr2 | 141161022 | 0.29 | 0.08 | 0.28 | 22.3 |
| SiWD179 | SiChr8 | GRMZM2G011364 | ZmChr4 | 5702974 | 0.24 | 0.07 | 0.29 | 18.5 |
| SiWD180 | SiChr8 | GRMZM2G126552 | ZmChr4 | 2764644 | 0.25 | 0.07 | 0.28 | 19.2 |
| SiWD181 | SiChr9 | GRMZM2G036169 | ZmChr1 | 300271918 | 0.24 | 0.08 | 0.33 | 18.5 |
| SiWD182 | SiChr9 | GRMZM2G056916 | ZmChr1 | 299882442 | 0.26 | 0.09 | 0.35 | 20.0 |
| SiWD183 | SiChr9 | GRMZM2G058511 | ZmChr1 | 298579689 | 0.26 | 0.08 | 0.31 | 20.0 |
| SiWD184 | SiChr9 | GRMZM2G312091 | ZmChr1 | 288862553 | 0.29 | 0.08 | 0.28 | 22.3 |
| SiWD185 | SiChr9 | GRMZM2G024051 | ZmChr1 | 277584479 | 0.29 | 0.09 | 0.31 | 22.3 |
| SiWD186 | SiChr9 | GRMZM2G131998 | ZmChr5 | 7523923 | 0.27 | 0.09 | 0.33 | 20.8 |
| SiWD187 | SiChr9 | GRMZM2G078252 | ZmChr1 | 275688123 | 0.26 | 0.07 | 0.27 | 20.0 |
| SiWD188 | SiChr9 | GRMZM2G056645 | ZmChr1 | 275248687 | 0.26 | 0.07 | 0.27 | 20.0 |
| SiWD189 | SiChr9 | GRMZM2G317614 | ZmChr5 | 7982533 | 0.28 | 0.07 | 0.25 | 21.5 |
| SiWD190 | SiChr9 | GRMZM2G133749 | ZmChr1 | 274064762 | 0.29 | 0.06 | 0.21 | 22.3 |
| SiWD191 | SiChr9 | GRMZM2G065822 | ZmChr5 | 8426059 | 0.24 | 0.08 | 0.33 | 18.5 |
| SiWD192 | SiChr9 | GRMZM2G088162 | ZmChr1 | 271027511 | 0.24 | 0.09 | 0.38 | 18.5 |
| SiWD193 | SiChr9 | GRMZM2G010054 | ZmChr1 | 267609498 | 0.24 | 0.07 | 0.29 | 18.5 |
| SiWD194 | SiChr9 | GRMZM2G300945 | ZmChr5 | 12397184 | 0.25 | 0.07 | 0.28 | 19.2 |
| SiWD196 | SiChr9 | GRMZM2G045314 | ZmChr1 | 257998950 | 0.24 | 0.08 | 0.33 | 18.5 |
| SiWD197 | SiChr9 | GRMZM2G015005 | ZmChr1 | 251611877 | 0.27 | 0.08 | 0.30 | 20.8 |
| SiWD198 | SiChr9 | GRMZM2G090217 | ZmChr1 | 251812479 | 0.28 | 0.09 | 0.32 | 21.5 |
| SiWD199 | SiChr9 | GRMZM2G099023 | ZmChr1 | 253911424 | 0.29 | 0.08 | 0.28 | 22.3 |
| SiWD200 | SiChr9 | GRMZM2G150772 | ZmChr1 | 256071969 | 0.24 | 0.07 | 0.29 | 18.5 |
| SiWD201 | SiChr9 | GRMZM2G406553 | ZmChr10 | 148293596 | 0.25 | 0.07 | 0.28 | 19.2 |
| SiWD202 | SiChr9 | GRMZM2G017089 | ZmChr1 | 254094619 | 0.24 | 0.07 | 0.29 | 18.5 |
| SiWD203 | SiChr9 | GRMZM2G037698 | ZmChr1 | 233997474 | 0.25 | 0.07 | 0.28 | 19.2 |
| SiWD204 | SiChr9 | GRMZM2G097640 | ZmChr1 | 233823192 | 0.25 | 0.08 | 0.32 | 19.2 |
| SiWD205 | SiChr9 | GRMZM2G058345 | ZmChr5 | 30947253 | 0.24 | 0.09 | 0.38 | 18.5 |
| SiWD206 | SiChr9 | GRMZM2G458888 | ZmChr3 | 229900075 | 0.26 | 0.09 | 0.35 | 20.0 |
| SiWD207 | SiChr9 | GRMZM2G374969 | ZmChr7 | 160216902 | 0.26 | 0.08 | 0.31 | 20.0 |
| SiWD208 | SiChr9 | GRMZM2G123709 | ZmChr9 | 126194974 | 0.24 | 0.07 | 0.29 | 18.5 |
| SiWD209 | SiChr9 | GRMZM5G899300 | ZmChr9 | 20597214 | 0.25 | 0.07 | 0.28 | 19.2 |
| SiWD210 | SiChr9 | GRMZM2G136014 | ZmChr3 | 231710665 | 0.27 | 0.08 | 0.30 | 20.8 |
| SiWD211 | SiChr9 | GRMZM2G085825 | ZmChr1 | 69086582 | 0.28 | 0.09 | 0.32 | 21.5 |
| SiWD212 | SiChr9 | GRMZM2G101689 | ZmChr1 | 60861638 | 0.29 | 0.08 | 0.28 | 22.3 |
| SiWD214 | SiChr9 | GRMZM2G095043 | ZmChr1 | 47130522 | 0.24 | 0.07 | 0.29 | 18.5 |
| SiWD215 | SiChr9 | GRMZM2G179411 | ZmChr1 | 43971021 | 0.25 | 0.07 | 0.28 | 19.2 |
| SiWD216 | SiChr9 | GRMZM5G803381 | ZmChr9 | 41068404 | 0.24 | 0.08 | 0.33 | 18.5 |
| SiWD217 | SiChr9 | GRMZM2G030422 | ZmChr1 | 35239930 | 0.26 | 0.09 | 0.35 | 20.0 |
| SiWD218 | SiChr9 | GRMZM5G896834 | ZmChr1 | 21377569 | 0.25 | 0.08 | 0.32 | 19.2 |
| SiWD219 | SiChr9 | GRMZM2G063603 | ZmChr9 | 149806803 | 0.24 | 0.09 | 0.38 | 18.5 |
| SiWD222 | SiChr9 | GRMZM2G159427 | ZmChr1 | 4997170 | 0.26 | 0.09 | 0.35 | 20.0 |
| SiWD224 | SiChr9 | GRMZM2G426802 | ZmChr1 | 3364324 | 0.26 | 0.08 | 0.31 | 20.0 |
| SiWD225 | SiChr9 | GRMZM2G092723 | ZmChr9 | 154427090 | 0.26 | 0.08 | 0.31 | 20.0 |
| **Mean** | | | | | **0.27** | **0.08** | **0.30** | **20.8** |

| **Foxtail millet - Sorghum** | | | | | | | | |
| --- | --- | --- | --- | --- | --- | --- | --- | --- |
| **NIPGR ID** | **Chromosome** | **Gene IDs** | **Chromosome** | **Positions** | **Ks** | **Ka** | **Ka/Ks** | **Mya** |
| SiWD001 | SiChr1 | Sobic.004G270800 | SbChr04 | 60803546 | 0.21 | 0.04 | 0.19 | 16.2 |
| SiWD002 | SiChr1 | Sobic.004G106700 | SbChr04 | 10206701 | 0.25 | 0.07 | 0.28 | 19.2 |
| SiWD003 | SiChr1 | Sobic.004G096400 | SbChr04 | 8550225 | 0.24 | 0.06 | 0.25 | 18.5 |
| SiWD005 | SiChr1 | Sobic.004G086700 | SbChr04 | 7309607 | 0.26 | 0.06 | 0.23 | 20.0 |
| SiWD006 | SiChr1 | Sobic.004G051200 | SbChr04 | 4139995 | 0.21 | 0.04 | 0.19 | 16.2 |
| SiWD007 | SiChr1 | Sobic.003G427100 | SbChr03 | 73075737 | 0.21 | 0.04 | 0.19 | 16.2 |
| SiWD008 | SiChr1 | Sobic.004G034700 | SbChr04 | 2810434 | 0.21 | 0.04 | 0.19 | 16.2 |
| SiWD009 | SiChr1 | Sobic.004G034300 | SbChr04 | 2783050 | 0.25 | 0.07 | 0.28 | 19.2 |
| SiWD010 | SiChr1 | Sobic.004G023100 | SbChr04 | 1899380 | 0.24 | 0.06 | 0.25 | 18.5 |
| SiWD011 | SiChr1 | Sobic.004G145500 | SbChr04 | 44207421 | 0.26 | 0.06 | 0.23 | 20.0 |
| SiWD012 | SiChr1 | Sobic.002G150800 | SbChr02 | 44045669 | 0.21 | 0.04 | 0.19 | 16.2 |
| SiWD013 | SiChr1 | Sobic.004G157400 | SbChr04 | 49207615 | 0.22 | 0.04 | 0.18 | 16.9 |
| SiWD014 | SiChr1 | Sobic.004G161600 | SbChr04 | 50344713 | 0.21 | 0.04 | 0.19 | 16.2 |
| SiWD015 | SiChr1 | Sobic.004G169600 | SbChr04 | 51366154 | 0.25 | 0.07 | 0.28 | 19.2 |
| SiWD016 | SiChr1 | Sobic.004G171200 | SbChr04 | 51650879 | 0.24 | 0.06 | 0.25 | 18.5 |
| SiWD018 | SiChr1 | Sobic.004G198300 | SbChr04 | 54211117 | 0.26 | 0.06 | 0.23 | 20.0 |
| SiWD019 | SiChr1 | Sobic.004G215800 | SbChr04 | 55845941 | 0.21 | 0.04 | 0.19 | 16.2 |
| SiWD020 | SiChr1 | Sobic.004G227500 | SbChr04 | 56999534 | 0.22 | 0.05 | 0.23 | 16.9 |
| SiWD022 | SiChr1 | Sobic.004G283000 | SbChr04 | 61790288 | 0.21 | 0.04 | 0.19 | 16.2 |
| SiWD024 | SiChr1 | Sobic.004G257400 | SbChr04 | 59645973 | 0.21 | 0.04 | 0.19 | 16.2 |
| SiWD026 | SiChr1 | Sobic.004G256500 | SbChr04 | 59553433 | 0.25 | 0.07 | 0.28 | 19.2 |
| SiWD027 | SiChr1 | Sobic.004G242900 | SbChr04 | 58407095 | 0.24 | 0.06 | 0.25 | 18.5 |
| SiWD028 | SiChr1 | Sobic.004G312600 | SbChr04 | 64265699 | 0.26 | 0.06 | 0.23 | 20.0 |
| SiWD029 | SiChr1 | Sobic.004G322300 | SbChr04 | 65027963 | 0.21 | 0.04 | 0.19 | 16.2 |
| SiWD030 | SiChr1 | Sobic.004G327100 | SbChr04 | 65502744 | 0.22 | 0.04 | 0.18 | 16.9 |
| SiWD031 | SiChr1 | Sobic.004G331100 | SbChr04 | 65754655 | 0.21 | 0.04 | 0.19 | 16.2 |
| SiWD032 | SiChr1 | Sobic.004G341700 | SbChr04 | 66604550 | 0.25 | 0.07 | 0.28 | 19.2 |
| SiWD033 | SiChr1 | Sobic.004G345400 | SbChr04 | 66852471 | 0.24 | 0.06 | 0.25 | 18.5 |
| SiWD034 | SiChr2 | Sobic.002G006400 | SbChr02 | 616052 | 0.26 | 0.06 | 0.23 | 20.0 |
| SiWD035 | SiChr2 | Sobic.001G462600 | SbChr01 | 66516723 | 0.21 | 0.04 | 0.19 | 16.2 |
| SiWD036 | SiChr2 | Sobic.002G020700 | SbChr02 | 1949298 | 0.23 | 0.04 | 0.17 | 17.7 |
| SiWD037 | SiChr2 | Sobic.002G044000 | SbChr02 | 4184974 | 0.25 | 0.06 | 0.24 | 19.2 |
| SiWD039 | SiChr2 | Sobic.002G060900 | SbChr02 | 5855339 | 0.23 | 0.06 | 0.26 | 17.7 |
| SiWD040 | SiChr2 | Sobic.002G090700 | SbChr02 | 9480604 | 0.21 | 0.05 | 0.24 | 16.2 |
| SiWD041 | SiChr2 | Sobic.002G092300 | SbChr02 | 9606827 | 0.21 | 0.04 | 0.19 | 16.2 |
| SiWD042 | SiChr2 | Sobic.003G156500 | SbChr03 | 17292248 | 0.25 | 0.07 | 0.28 | 19.2 |
| SiWD043 | SiChr2 | Sobic.001G071400 | SbChr01 | 5424209 | 0.24 | 0.06 | 0.25 | 18.5 |
| SiWD044 | SiChr2 | Sobic.002G160700 | SbChr02 | 49665075 | 0.26 | 0.06 | 0.23 | 20.0 |
| SiWD045 | SiChr2 | Sobic.002G181400 | SbChr02 | 56225783 | 0.21 | 0.04 | 0.19 | 16.2 |
| SiWD046 | SiChr2 | Sobic.002G217500 | SbChr02 | 60948859 | 0.22 | 0.04 | 0.18 | 16.9 |
| SiWD047 | SiChr2 | Sobic.002G280700 | SbChr02 | 66165351 | 0.21 | 0.05 | 0.24 | 16.2 |
| SiWD048 | SiChr2 | Sobic.002G347000 | SbChr02 | 71231658 | 0.25 | 0.06 | 0.24 | 19.2 |
| SiWD049 | SiChr2 | Sobic.002G365900 | SbChr02 | 72558552 | 0.24 | 0.05 | 0.21 | 18.5 |
| SiWD050 | SiChr2 | Sobic.002G381700 | SbChr02 | 73771973 | 0.26 | 0.05 | 0.19 | 20.0 |
| SiWD051 | SiChr2 | Sobic.002G401500 | SbChr02 | 75149825 | 0.21 | 0.06 | 0.29 | 16.2 |
| SiWD052 | SiChr2 | Sobic.002G430000 | SbChr02 | 77526377 | 0.23 | 0.04 | 0.17 | 17.7 |
| SiWD053 | SiChr3 | Sobic.006G267800 | SbChr06 | 61027050 | 0.24 | 0.04 | 0.17 | 18.5 |
| SiWD054 | SiChr3 | Sobic.006G267500 | SbChr06 | 60995695 | 0.25 | 0.04 | 0.16 | 19.2 |
| SiWD055 | SiChr3 | Sobic.006G264000 | SbChr06 | 60773548 | 0.22 | 0.06 | 0.27 | 16.9 |
| SiWD056 | SiChr3 | Sobic.009G040400 | SbChr09 | 3923117 | 0.24 | 0.07 | 0.29 | 18.5 |
| SiWD058 | SiChr3 | Sobic.009G060000 | SbChr09 | 6304640 | 0.24 | 0.07 | 0.29 | 18.5 |
| SiWD059 | SiChr3 | Sobic.001G222000 | SbChr01 | 21075268 | 0.23 | 0.06 | 0.26 | 17.7 |
| SiWD061 | SiChr3 | Sobic.009G258500 | SbChr09 | 59229183 | 0.25 | 0.04 | 0.16 | 19.2 |
| SiWD062 | SiChr3 | Sobic.009G238000 | SbChr09 | 57607389 | 0.21 | 0.06 | 0.29 | 16.2 |
| SiWD064 | SiChr3 | Sobic.007G061000 | SbChr07 | 6363293 | 0.23 | 0.04 | 0.17 | 17.7 |
| SiWD065 | SiChr3 | Sobic.009G223300 | SbChr09 | 56598166 | 0.24 | 0.04 | 0.17 | 18.5 |
| SiWD066 | SiChr3 | Sobic.009G216700 | SbChr09 | 56105853 | 0.25 | 0.04 | 0.16 | 19.2 |
| SiWD067 | SiChr3 | Sobic.009G208200 | SbChr09 | 55554920 | 0.22 | 0.06 | 0.27 | 16.9 |
| SiWD068 | SiChr3 | Sobic.009G201200 | SbChr09 | 55096427 | 0.24 | 0.07 | 0.29 | 18.5 |
| SiWD069 | SiChr3 | Sobic.003G029500 | SbChr03 | 2595736 | 0.24 | 0.07 | 0.29 | 18.5 |
| SiWD070 | SiChr3 | Sobic.009G134500 | SbChr09 | 48959256 | 0.23 | 0.06 | 0.26 | 17.7 |
| SiWD071 | SiChr3 | Sobic.009G133500 | SbChr09 | 48816513 | 0.25 | 0.04 | 0.16 | 19.2 |
| SiWD072 | SiChr3 | Sobic.009G112800 | SbChr09 | 45297225 | 0.21 | 0.04 | 0.19 | 16.2 |
| SiWD073 | SiChr3 | Sobic.006G195700 | SbChr06 | 55793598 | 0.25 | 0.07 | 0.28 | 19.2 |
| SiWD074 | SiChr3 | Sobic.003G248500 | SbChr03 | 58763595 | 0.24 | 0.06 | 0.25 | 18.5 |
| SiWD075 | SiChr3 | Sobic.008G150400 | SbChr08 | 50950836 | 0.27 | 0.09 | 0.33 | 20.8 |
| SiWD077 | SiChr3 | Sobic.008G163500 | SbChr08 | 52340464 | 0.21 | 0.06 | 0.29 | 16.2 |
| SiWD078 | SiChr3 | Sobic.008G171800 | SbChr08 | 53214921 | 0.21 | 0.06 | 0.29 | 16.2 |
| SiWD079 | SiChr4 | Sobic.010G017900 | SbChr10 | 1482105 | 0.23 | 0.04 | 0.17 | 17.7 |
| SiWD080 | SiChr4 | Sobic.010G021500 | SbChr10 | 1725975 | 0.24 | 0.04 | 0.17 | 18.5 |
| SiWD082 | SiChr4 | Sobic.010G056200 | SbChr10 | 4379247 | 0.25 | 0.04 | 0.16 | 19.2 |
| SiWD086 | SiChr4 | Sobic.004G088500 | SbChr04 | 7501381 | 0.22 | 0.06 | 0.27 | 16.9 |
| SiWD087 | SiChr4 | Sobic.006G219000 | SbChr06 | 57537030 | 0.24 | 0.07 | 0.29 | 18.5 |
| SiWD089 | SiChr4 | Sobic.010G147900 | SbChr10 | 42235963 | 0.24 | 0.07 | 0.29 | 18.5 |
| SiWD090 | SiChr4 | Sobic.010G213200 | SbChr10 | 55359432 | 0.23 | 0.06 | 0.26 | 17.7 |
| SiWD091 | SiChr4 | Sobic.010G209400 | SbChr10 | 55038614 | 0.25 | 0.04 | 0.16 | 19.2 |
| SiWD092 | SiChr4 | Sobic.010G205900 | SbChr10 | 54640289 | 0.22 | 0.04 | 0.18 | 16.9 |
| SiWD093 | SiChr4 | Sobic.010G181600 | SbChr10 | 51831702 | 0.22 | 0.04 | 0.18 | 16.9 |
| SiWD094 | SiChr4 | Sobic.010G178600 | SbChr10 | 51411995 | 0.21 | 0.05 | 0.24 | 16.2 |
| SiWD095 | SiChr4 | Sobic.010G169900 | SbChr10 | 49837256 | 0.25 | 0.06 | 0.24 | 19.2 |
| SiWD096 | SiChr4 | Sobic.010G229700 | SbChr10 | 57024987 | 0.24 | 0.05 | 0.21 | 18.5 |
| SiWD097 | SiChr4 | Sobic.010G262800 | SbChr10 | 59732139 | 0.26 | 0.05 | 0.19 | 20.0 |
| SiWD098 | SiChr4 | Sobic.010G269500 | SbChr10 | 60214519 | 0.26 | 0.05 | 0.19 | 20.0 |
| SiWD100 | SiChr5 | Sobic.003G002700 | SbChr03 | 310510 | 0.22 | 0.07 | 0.32 | 16.9 |
| SiWD101 | SiChr5 | Sobic.003G006500 | SbChr03 | 565865 | 0.21 | 0.06 | 0.29 | 16.2 |
| SiWD102 | SiChr5 | Sobic.003G008100 | SbChr03 | 709752 | 0.23 | 0.06 | 0.26 | 17.7 |
| SiWD104 | SiChr5 | Sobic.003G115900 | SbChr03 | 10459461 | 0.25 | 0.04 | 0.16 | 19.2 |
| SiWD105 | SiChr5 | Sobic.003G089100 | SbChr03 | 7758246 | 0.22 | 0.04 | 0.18 | 16.9 |
| SiWD106 | SiChr5 | Sobic.003G077600 | SbChr03 | 6631153 | 0.25 | 0.06 | 0.24 | 19.2 |
| SiWD107 | SiChr5 | Sobic.003G053800 | SbChr03 | 4875019 | 0.24 | 0.05 | 0.21 | 18.5 |
| SiWD108 | SiChr5 | Sobic.003G046400 | SbChr03 | 4239415 | 0.23 | 0.06 | 0.26 | 17.7 |
| SiWD109 | SiChr5 | Sobic.003G037700 | SbChr03 | 3546550 | 0.25 | 0.04 | 0.16 | 19.2 |
| SiWD111 | SiChr5 | Sobic.003G036800 | SbChr03 | 3385500 | 0.22 | 0.04 | 0.18 | 16.9 |
| SiWD112 | SiChr5 | Sobic.003G057700 | SbChr03 | 5108214 | 0.22 | 0.04 | 0.18 | 16.9 |
| SiWD113 | SiChr5 | Sobic.009G235000 | SbChr09 | 57428432 | 0.21 | 0.05 | 0.24 | 16.2 |
| SiWD114 | SiChr5 | Sobic.003G021200 | SbChr03 | 1812014 | 0.25 | 0.06 | 0.24 | 19.2 |
| SiWD115 | SiChr5 | Sobic.003G151200 | SbChr03 | 15911395 | 0.24 | 0.05 | 0.21 | 18.5 |
| SiWD116 | SiChr5 | Sobic.003G152400 | SbChr03 | 16186558 | 0.23 | 0.06 | 0.26 | 17.7 |
| SiWD118 | SiChr5 | Sobic.003G186000 | SbChr03 | 50318472 | 0.25 | 0.04 | 0.16 | 19.2 |
| SiWD119 | SiChr5 | Sobic.003G195900 | SbChr03 | 52021654 | 0.22 | 0.04 | 0.18 | 16.9 |
| SiWD120 | SiChr5 | Sobic.003G209200 | SbChr03 | 54134554 | 0.26 | 0.05 | 0.19 | 20.0 |
| SiWD121 | SiChr5 | Sobic.003G214600 | SbChr03 | 54884703 | 0.21 | 0.07 | 0.33 | 16.2 |
| SiWD122 | SiChr5 | Sobic.003G214700 | SbChr03 | 54902617 | 0.22 | 0.04 | 0.18 | 16.9 |
| SiWD123 | SiChr5 | Sobic.003G223500 | SbChr03 | 55937680 | 0.22 | 0.04 | 0.18 | 16.9 |
| SiWD124 | SiChr5 | Sobic.003G231900 | SbChr03 | 57158606 | 0.21 | 0.05 | 0.24 | 16.2 |
| SiWD125 | SiChr5 | Sobic.003G240700 | SbChr03 | 57989127 | 0.25 | 0.06 | 0.24 | 19.2 |
| SiWD126 | SiChr5 | Sobic.006G041600 | SbChr06 | 26656272 | 0.24 | 0.05 | 0.21 | 18.5 |
| SiWD127 | SiChr5 | Sobic.003G261100 | SbChr03 | 59910277 | 0.26 | 0.05 | 0.19 | 20.0 |
| SiWD128 | SiChr5 | Sobic.001G036100 | SbChr01 | 2697169 | 0.26 | 0.05 | 0.19 | 20.0 |
| SiWD129 | SiChr5 | Sobic.003G274400 | SbChr03 | 61069940 | 0.22 | 0.07 | 0.32 | 16.9 |
| SiWD130 | SiChr5 | Sobic.003G282000 | SbChr03 | 61702216 | 0.21 | 0.06 | 0.29 | 16.2 |
| SiWD131 | SiChr5 | Sobic.003G314300 | SbChr03 | 64266956 | 0.23 | 0.06 | 0.26 | 17.7 |
| SiWD132 | SiChr5 | Sobic.003G316700 | SbChr03 | 64475624 | 0.25 | 0.04 | 0.16 | 19.2 |
| SiWD133 | SiChr5 | Sobic.003G320700 | SbChr03 | 64855410 | 0.22 | 0.04 | 0.18 | 16.9 |
| SiWD134 | SiChr5 | Sobic.003G362500 | SbChr03 | 68004330 | 0.26 | 0.06 | 0.23 | 20.0 |
| SiWD135 | SiChr5 | Sobic.003G362600 | SbChr03 | 68008739 | 0.21 | 0.04 | 0.19 | 16.2 |
| SiWD136 | SiChr5 | Sobic.003G408300 | SbChr03 | 71607589 | 0.22 | 0.04 | 0.18 | 16.9 |
| SiWD137 | SiChr5 | Sobic.003G416400 | SbChr03 | 72273806 | 0.24 | 0.04 | 0.17 | 18.5 |
| SiWD139 | SiChr5 | Sobic.003G423000 | SbChr03 | 72795188 | 0.26 | 0.06 | 0.23 | 20.0 |
| SiWD140 | SiChr5 | Sobic.003G444100 | SbChr03 | 74274313 | 0.21 | 0.07 | 0.33 | 16.2 |
| SiWD141 | SiChr6 | Sobic.007G008300 | SbChr07 | 741172 | 0.22 | 0.04 | 0.18 | 16.9 |
| SiWD142 | SiChr6 | Sobic.007G051700 | SbChr07 | 5245534 | 0.22 | 0.04 | 0.18 | 16.9 |
| SiWD143 | SiChr6 | Sobic.007G032400 | SbChr07 | 2867565 | 0.21 | 0.05 | 0.24 | 16.2 |
| SiWD144 | SiChr6 | Sobic.007G028100 | SbChr07 | 2539833 | 0.24 | 0.04 | 0.17 | 18.5 |
| SiWD145 | SiChr6 | Sobic.003G201000 | SbChr03 | 52995227 | 0.26 | 0.06 | 0.23 | 20.0 |
| SiWD148 | SiChr6 | Sobic.007G123900 | SbChr07 | 51846034 | 0.21 | 0.07 | 0.33 | 16.2 |
| SiWD149 | SiChr6 | Sobic.007G220300 | SbChr07 | 63614182 | 0.21 | 0.04 | 0.19 | 16.2 |
| SiWD150 | SiChr6 | Sobic.007G219000 | SbChr07 | 63477351 | 0.22 | 0.04 | 0.18 | 16.9 |
| SiWD151 | SiChr6 | Sobic.007G198300 | SbChr07 | 61728041 | 0.24 | 0.04 | 0.17 | 18.5 |
| SiWD152 | SiChr6 | Sobic.007G193900 | SbChr07 | 61412615 | 0.26 | 0.06 | 0.23 | 20.0 |
| SiWD153 | SiChr6 | Sobic.006G182200 | SbChr06 | 54630281 | 0.21 | 0.07 | 0.33 | 16.2 |
| SiWD154 | SiChr6 | Sobic.007G171100 | SbChr07 | 59382939 | 0.22 | 0.04 | 0.18 | 16.9 |
| SiWD155 | SiChr6 | Sobic.007G173700 | SbChr07 | 59592548 | 0.22 | 0.04 | 0.18 | 16.9 |
| SiWD156 | SiChr7 | Sobic.006G019300 | SbChr06 | 3277578 | 0.21 | 0.05 | 0.24 | 16.2 |
| SiWD157 | SiChr7 | Sobic.010G147700 | SbChr10 | 42198151 | 0.22 | 0.04 | 0.18 | 16.9 |
| SiWD158 | SiChr7 | Sobic.006G075600 | SbChr06 | 44887244 | 0.22 | 0.04 | 0.18 | 16.9 |
| SiWD159 | SiChr7 | Sobic.006G141000 | SbChr06 | 51240582 | 0.21 | 0.05 | 0.24 | 16.2 |
| SiWD160 | SiChr7 | Sobic.006G156300 | SbChr06 | 52455787 | 0.25 | 0.06 | 0.24 | 19.2 |
| SiWD161 | SiChr7 | Sobic.006G173200 | SbChr06 | 53839694 | 0.24 | 0.05 | 0.21 | 18.5 |
| SiWD162 | SiChr7 | Sobic.006G182500 | SbChr06 | 54690890 | 0.26 | 0.05 | 0.19 | 20.0 |
| SiWD163 | SiChr7 | Sobic.006G198600 | SbChr06 | 56018479 | 0.26 | 0.05 | 0.19 | 20.0 |
| SiWD166 | SiChr7 | Sobic.008G057600 | SbChr08 | 5975780 | 0.22 | 0.07 | 0.32 | 16.9 |
| SiWD167 | SiChr7 | Sobic.008G054500 | SbChr08 | 5484721 | 0.21 | 0.06 | 0.29 | 16.2 |
| SiWD168 | SiChr7 | Sobic.008G047800 | SbChr08 | 4666143 | 0.21 | 0.06 | 0.29 | 16.2 |
| SiWD169 | SiChr7 | Sobic.008G017200 | SbChr08 | 1411538 | 0.23 | 0.04 | 0.17 | 17.7 |
| SiWD170 | SiChr7 | Sobic.008G016100 | SbChr08 | 1331417 | 0.24 | 0.04 | 0.17 | 18.5 |
| SiWD172 | SiChr7 | Sobic.008G009700 | SbChr08 | 776103 | 0.25 | 0.04 | 0.16 | 19.2 |
| SiWD175 | SiChr8 | Sobic.005G059600 | SbChr05 | 6220170 | 0.22 | 0.06 | 0.27 | 16.9 |
| SiWD176 | SiChr8 | Sobic.005G063700 | SbChr05 | 7038413 | 0.24 | 0.07 | 0.29 | 18.5 |
| SiWD177 | SiChr8 | Sobic.005G067200 | SbChr05 | 7704501 | 0.24 | 0.07 | 0.29 | 18.5 |
| SiWD178 | SiChr8 | Sobic.005G043600 | SbChr05 | 4143627 | 0.24 | 0.04 | 0.17 | 18.5 |
| SiWD179 | SiChr8 | Sobic.005G184200 | SbChr05 | 57328541 | 0.26 | 0.06 | 0.23 | 20.0 |
| SiWD180 | SiChr8 | Sobic.005G206700 | SbChr05 | 59687911 | 0.21 | 0.07 | 0.33 | 16.2 |
| SiWD181 | SiChr9 | Sobic.001G001900 | SbChr01 | 155260 | 0.21 | 0.06 | 0.29 | 16.2 |
| SiWD182 | SiChr9 | Sobic.001G004100 | SbChr01 | 355515 | 0.21 | 0.06 | 0.29 | 16.2 |
| SiWD183 | SiChr9 | Sobic.001G010300 | SbChr01 | 919606 | 0.21 | 0.05 | 0.24 | 16.2 |
| SiWD184 | SiChr9 | Sobic.001G054000 | SbChr01 | 4038118 | 0.25 | 0.06 | 0.24 | 19.2 |
| SiWD185 | SiChr9 | Sobic.001G085800 | SbChr01 | 6633021 | 0.24 | 0.05 | 0.21 | 18.5 |
| SiWD186 | SiChr9 | Sobic.001G091900 | SbChr01 | 7096696 | 0.26 | 0.05 | 0.19 | 20.0 |
| SiWD187 | SiChr9 | Sobic.001G092100 | SbChr01 | 7109275 | 0.26 | 0.05 | 0.19 | 20.0 |
| SiWD188 | SiChr9 | Sobic.001G093800 | SbChr01 | 7187522 | 0.22 | 0.07 | 0.32 | 16.9 |
| SiWD189 | SiChr9 | Sobic.001G097000 | SbChr01 | 7447770 | 0.22 | 0.07 | 0.32 | 16.9 |
| SiWD190 | SiChr9 | Sobic.001G097400 | SbChr01 | 7486977 | 0.21 | 0.06 | 0.29 | 16.2 |
| SiWD191 | SiChr9 | Sobic.001G099900 | SbChr01 | 7632214 | 0.21 | 0.06 | 0.29 | 16.2 |
| SiWD192 | SiChr9 | Sobic.001G107300 | SbChr01 | 8272402 | 0.23 | 0.04 | 0.17 | 17.7 |
| SiWD193 | SiChr9 | Sobic.001G117800 | SbChr01 | 9153351 | 0.24 | 0.04 | 0.17 | 18.5 |
| SiWD194 | SiChr9 | Sobic.001G126800 | SbChr01 | 9931269 | 0.25 | 0.04 | 0.16 | 19.2 |
| SiWD195 | SiChr9 | Sobic.005G172500 | SbChr05 | 55620474 | 0.22 | 0.06 | 0.27 | 16.9 |
| SiWD196 | SiChr9 | Sobic.001G142100 | SbChr01 | 11375505 | 0.21 | 0.05 | 0.24 | 16.2 |
| SiWD197 | SiChr9 | Sobic.001G157300 | SbChr01 | 12685267 | 0.25 | 0.06 | 0.24 | 19.2 |
| SiWD198 | SiChr9 | Sobic.001G157600 | SbChr01 | 12740306 | 0.24 | 0.05 | 0.21 | 18.5 |
| SiWD199 | SiChr9 | Sobic.001G163600 | SbChr01 | 13442492 | 0.26 | 0.05 | 0.19 | 20.0 |
| SiWD200 | SiChr9 | Sobic.001G170100 | SbChr01 | 14159821 | 0.26 | 0.05 | 0.19 | 20.0 |
| SiWD201 | SiChr9 | Sobic.001G207400 | SbChr01 | 18891147 | 0.22 | 0.07 | 0.32 | 16.9 |
| SiWD202 | SiChr9 | Sobic.006G017000 | SbChr06 | 2639939 | 0.21 | 0.05 | 0.24 | 16.2 |
| SiWD203 | SiChr9 | Sobic.001G226000 | SbChr01 | 21563812 | 0.23 | 0.04 | 0.17 | 17.7 |
| SiWD204 | SiChr9 | Sobic.001G226700 | SbChr01 | 21663854 | 0.21 | 0.04 | 0.19 | 16.2 |
| SiWD205 | SiChr9 | Sobic.001G227400 | SbChr01 | 21753429 | 0.23 | 0.04 | 0.17 | 17.7 |
| SiWD207 | SiChr9 | Sobic.001G296800 | SbChr01 | 50494549 | 0.21 | 0.04 | 0.19 | 16.2 |
| SiWD208 | SiChr9 | Sobic.001G310100 | SbChr01 | 52396064 | 0.26 | 0.05 | 0.19 | 20.0 |
| SiWD209 | SiChr9 | Sobic.010G032400 | SbChr10 | 2587831 | 0.21 | 0.05 | 0.24 | 16.2 |
| SiWD210 | SiChr9 | Sobic.008G040100 | SbChr08 | 3854396 | 0.23 | 0.04 | 0.17 | 17.7 |
| SiWD211 | SiChr9 | Sobic.001G346300 | SbChr01 | 56379783 | 0.21 | 0.04 | 0.19 | 16.2 |
| SiWD212 | SiChr9 | Sobic.001G370300 | SbChr01 | 58668296 | 0.26 | 0.05 | 0.19 | 20.0 |
| SiWD213 | SiChr9 | Sobic.003G059700 | SbChr03 | 5261082 | 0.26 | 0.05 | 0.19 | 20.0 |
| SiWD214 | SiChr9 | Sobic.001G401400 | SbChr01 | 61501223 | 0.22 | 0.07 | 0.32 | 16.9 |
| SiWD215 | SiChr9 | Sobic.001G409500 | SbChr01 | 62116037 | 0.21 | 0.06 | 0.29 | 16.2 |
| SiWD216 | SiChr9 | Sobic.010G126000 | SbChr10 | 15578449 | 0.21 | 0.06 | 0.29 | 16.2 |
| SiWD217 | SiChr9 | Sobic.001G433800 | SbChr01 | 64035363 | 0.23 | 0.04 | 0.17 | 17.7 |
| SiWD219 | SiChr9 | Sobic.001G477700 | SbChr01 | 67814123 | 0.24 | 0.04 | 0.17 | 18.5 |
| SiWD220 | SiChr9 | Sobic.001G501400 | SbChr01 | 69861557 | 0.25 | 0.04 | 0.16 | 19.2 |
| SiWD222 | SiChr9 | Sobic.001G526300 | SbChr01 | 71844510 | 0.22 | 0.06 | 0.27 | 16.9 |
| SiWD224 | SiChr9 | Sobic.001G532700 | SbChr01 | 72410863 | 0.22 | 0.07 | 0.32 | 16.9 |
| SiWD225 | SiChr9 | Sobic.001G535100 | SbChr01 | 72675217 | 0.21 | 0.06 | 0.29 | 16.2 |
| **Mean** | | | | | **0.26** | **0.06** | **0.23** | **19.2** |
