# Supplementary material for: Genome-Wide Investigation and Expression Analyses of WD40 Protein Family in the Model Plant Foxtail Millet (Setaria italica L.)
Source: PLoS One. 2014 Jan 23;9(1):e86852. doi: 10.1371/journal.pone.0086852 (PMC3900672; doi:10.1371/journal.pone.0086852)
Supplement: Table S8 — List of primers used in quantitative real time-PCR expression analysis of 13 SiWD40 genes. (DOC) [file pone.0086852.s011.doc]

**Table S8.** List of primers used in quantitative real time-PCR expression analysis of 13 *SiWD40* genes.

| **NAME** | **FORWARD PRIMERS (5′- 3′)** | **REVERSE PRIMERS (5′- 3′)** |
| --- | --- | --- |
| SiWD028 | CGTGCCATTATATTCCCAAAGTG | TCAAAACTTCGTGCCCTAAACC |
| SiWD037 | GGACAGTATTGAGTTCCCAGCATT | AAAGGAATTTGCGTGCAACAG |
| SiWD063 | CATACATTTTCCTTGGCATTCTGA | ACAACTAGACAGACAGAACTCAATTCCT |
| SiWD106 | AGGTTTCTGTTGCTTTTGGATTCT | TGAACCGTTAAAAGCCAACATTG |
| SiWD132 | TGTAATTTTGCCATAGCTTTGCA | TGCCAGCCCAAGTCAAAAC |
| SiWD137 | CAAAGTATGCTCTGTGGAGGGTTA | TCATAAAATAAAGATGCATGCTGACA |
| SiWD144 | ATGGTTGACTGGCACCTGTAAA | CGGCCGCGATTCTTCTT |
| SiWD155 | TCCGCCCTCTTGGGTGAT | GAAGCTACATCGGACTTTTTTGG |
| SiWD156 | TGCATCTCAGGCCAAAAACA | GCCGCCCTTCCCACAT |
| SiWD182 | GTGATGGAGGACTGAATGATGGT | TTTCCGTGCCTTTGGTTAGG |
| SiWD195 | TCTTGAGTAGCCGAATGAATGTTT | TCTCGCGGTGGCATCAG |
| SiWD202 | TTGCTGTGGCTGTATGAATGG | CAGCTGCCGCATATCTACTTGT |
| SiWD203 | CGATGCTCCCAATGTCATTTG | TCCCTCTTGTGTTGATGTATCCA |
| Actin2 | CGCATATGTGGCTCTTGACT | GGGCACCTAAATCTCTCTGC |
