# Supplementary material for: Genome-Wide Investigation and Expression Analyses of WD40 Protein Family in the Model Plant Foxtail Millet (Setaria italica L.)
Source: PLoS One. 2014 Jan 23;9(1):e86852. doi: 10.1371/journal.pone.0086852 (PMC3900672; doi:10.1371/journal.pone.0086852)
Supplement: Table S9 — Characteristics of 24 candidate SiWD40 proteins chosen for homology modeling. (DOC) [file pone.0086852.s012.doc]

**Table S9.** Characteristics of 24 candidate SiWD40 proteins chosen for homology modeling

| **Name of WD40 Protein** | **% of residues modelled** | **Characteristic feature** |
| --- | --- | --- |
| SiWD016 | 296 (98%) | 5 repeats |
| SiWD033 | 374 (99%) | 5 repeats |
| SiWD037 | 319 (98%) | 5 repeats |
| SiWD040 | 294 (98%) | 5 repeats |
| SiWD041 | 326 (93%) | 7 repeats |
| SiWD046 | 289 (95%) | 6 repeats |
| SiWD047 | 354 (88%) | HBRBBP4 domain |
| SiWD048 | 311 (95%) | 6 repeats |
| SiWD064 | 345 (100%) | 4 repeats |
| SiWD065 | 372 (95%) | 7 repeats |
| SiWD074 | 394 (96%) | 2 repeats |
| SiWD081 | 782 (86%) | Coatomer |
| SiWD086 | 249 (98%) | Coatomer |
| SiWD095 | 419 (82%) | 1 repeat |
| SiWD105 | 603 (99%) | 8 repeats |
| SiWD114 | 286 (94%) | 6 repeats |
| SiWD124 | 334 (97%) | 4 repeats |
| SiWD129 | 366 (81%) | HBRBBP4 domain |
| SiWD134 | 334 (97%) | 5 repeats |
| SiWD143 | 348 (92%) | 2 repeats |
| SiWD163 | 821 (93%) | UTP13 domain |
| SiWD192 | 312 (98%) | 1 repeat |
| SiWD196 | 380 (100%) | 7 repeats |
| SiWD198 | 379 (88%) | HBRBBP4 domain |
